# Special Issue: ASEAN Integration and its Health Implications

**DOI:** 10.3402/gha.v8.27368

**Published:** 2015-01-27

The World Health Organization (WHO) proposes the concept of Universal Health Coverage (UHC) as a ‘single overarching health goal’ for the next iteration of the Millennium Development Goals (MDGs) (1). UHC is defined as a situation where all people who need health services (prevention, promotion, treatment, rehabilitation, and palliative) receive them, without undue financial hardship (2). UHC includes three key aspects: the beneficiary – who is covered (population coverage or breadth coverage), the scope – which service is covered (service coverage or depth coverage), and the coverage – what is the level of financial contribution (financial coverage or height coverage) (2).

UHC is a critical component of sustainable development and poverty reduction, and a key element of any effort to reduce social inequities. UHC has a direct impact on a population's health and welfare. Financial risk protection prevents sick individuals and their families from being pushed into poverty when they have to pay for health services out of their own pockets. UHC is the hallmark of a government's commitment to improve the wellbeing of all its citizens. UHC requires health systems to be functional and effective, offering services that are widely available and of good quality (3).

Progress toward UHC is uneven in all countries. Globally, over 3 billion people – many of them in the poorest half of the world's population – must pay out of pocket (OOP) for health services. In 33 mostly lower-income countries, including many of the world's most populous nations, direct OOP payments account for more than 50% of total health expenditures. Worldwide, about 150 million people suffer financial catastrophe annually while 100 million are pushed below the poverty line as a result of catastrophic health spending. In some countries, up to 11% of the population suffers severe financial hardship each year as a result of catastrophic health spending and up to 5% is forced into poverty (2).

The Association of Southeast Asian Nations (ASEAN), consisting of 10 countries – Brunei, Cambodia, Indonesia, Lao PDR, Malaysia, Myanmar (Burma), the Philippines, Singapore, Thailand, and Vietnam – has been the most significant multilateral group in Asia for the past 45 years. Since its inception in 1967, ASEAN has accomplished several notable achievements in the economic and non-proliferation realms (4, 5). ASEAN is characterized by much diversity in terms of demographics, geography, society, economic development, political systems, and health outcomes (Table 1). These factors have not only contributed to the differences in health status of the region's diverse populations but also to the diverse nature of its health systems, which are at varying stages of evolution (6). Consequently, UHC progress in these countries varies.

**Table 1:** Selected socio-demographic and health indicators in the ASEAN countries

	Total population (000s), 2012^a^	Median age of population (years), 2012^a^	Population aged>60 years (%), 2012^a^	Population living in urban areas (%), 2012^a^	Crude birth rate (per 1,000 population), 2012^a^	Crude death rate (per 1,000 population), 2012^a^	NCDs age-standardized mortality rate (per 100,000 population) both sexes, 2012^a^	Literacy rate among adults aged ≥15 years (%), latest year^b^	Gross national income per capita (PPP int. $), 2012^a^
Brunei	412	30.1	7.0	76	15.9	3.5	475.3	95 (2012)^c^	No data
Cambodia	14,865	24.1	7.7	20	25.9	5.7	394	74 (2009)	2,330
Indonesia	246,864	27.5	7.9	51	19.2	5.3	680.1	93 (2011)	4,730
Lao PDR	6,646	21.0	5.8	35	27.3	7.0	680.0	73 (2005)	2,690
Malaysia	29,240	27.0	8.2	73	17.6	5.0	563.2	93 (2010)	16,270
Myanmar	52,797	28.6	8.2	33	17.4	8.3	708.7	93 (2012)^c^	No data
Philippines	96,707	22.7	6.2	49	24.6	5.9	720.0	95 (2008)	4,380
Singapore	5,303	37.9	15.1	100	9.9	4.4	264.8	96 (2012)^c^	60,110
Thailand	66,785	36.4	14.0	34	10.5	7.5	449.1	96 (2010)	9,280
Vietnam	90,796	29.4	9.3	32	15.9	5.7	435.4	94 (2009)	3,620

a World Health Statistics 2014

b UNESCO Institute for Statistics 2014

c UIC estimation.

The increasing multilateral collaboration between countries in the ASEAN region has led to the ambition to create the ASEAN Economic Community (AEC) by 2015. This regional economic integration aims to achieve a single market and production base, which is competitive, equitable, and integrated into global economy. The integration can potentially bring both positive and negative effects to country's effort in achieving UHC. This paper aims to describe the progress toward UHC in the ASEAN countries and discuss how regional integration could influence UHC.

## Methods

Data reported in this paper were obtained from published literature, reports, and gray literature available in the ASEAN countries. We used both online and manual search methods to gather the information and ‘snowball’ further data. The sources of online data include international and national journal articles and studies from multiple electronic bibliographic databases, including Ovid MEDLINE, PubMed and EMBASE, and web-based statistics such as World Health Statistics (http://www.who.int/gho/publications/world_health_statistics/en/); Global Health Observatory (GHO) (http://www.who.int/gho/en/); the Asian Development Bank Institute (http://www.adbi.org/); ASEAN (http://www.asean.org/), and the World Bank (http://data.worldbank.org/). The following main key search terms were used: UHC, health system, ASEAN integration, ASEAN countries, health insurance, health financing. In addition, search engines such as Google and Google Scholar were also used. The research team members conducted manual searches to collate government documents, reports, publications related to demographic, health system, and UHC in ASEAN member states.

## Results

### Progress of UHC in the ASEAN countries

In general, the ASEAN countries have made good progress toward UHC. Healthcare services, both preventive and curative care services, have been more and more available in many ASEAN countries. In some countries such as Cambodia, Lao PDR, and Vietnam, most preventive care services are separately provided under vertical national programs.

**Fig. 1:**
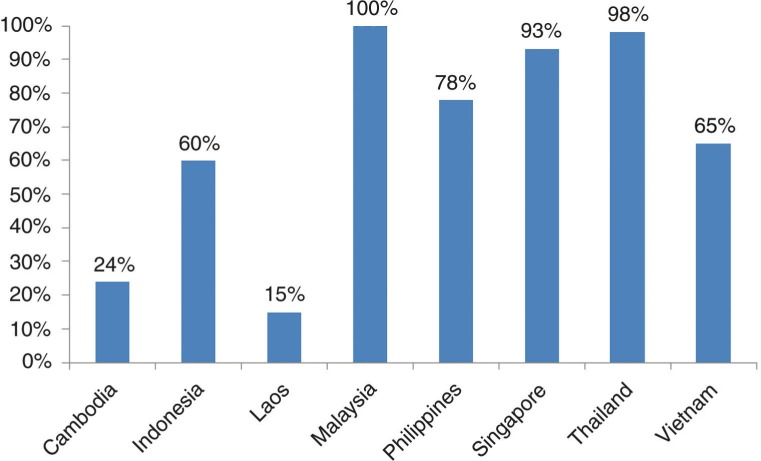
Coverage of health insurance in ASEAN countries 2012.

In the ASEAN countries, social health insurance (SHI) has been considered as an instrument for achieving the breadth of UHC. Significant progress has been made in expanding the coverage of health insurance, despite the existing gaps of insurance coverage across these countries (Fig. 1). As of 2012, Thailand's entire population is covered by SHI. In Malaysia, technically the entire population can use public health services funded via general taxation and low user charges whilst in Singapore, 93% of the population is covered by MediShield, the compulsory government organized health insurance scheme (7). In Indonesia, about 60% of the population is covered by health insurance. The Indonesian government rolled out the *Badan Penyelenggara Jaminan Sosial* (BPJS) *Kesehatan* on January 1, 2014, with an ambition to achieve national coverage of UHC by January 2019. This initiative is coordinated by the *BPJS* – the Social Security Administration, a national body under the auspices of the President of the Republic of Indonesia (8). The coverage of health insurance is however, still low in Lao PDR (15%) and Cambodia (24%). In Lao PDR, the government is now considering the creation of a national health insurance authority through the integration of the four different social health protection schemes. The expectation is that a unified institutional arrangement will lead to universal coverage by 2020. In Cambodia, good progress has been made in using health equity funds to cover the poor. However, civil servants and private sector employees are not covered at all by insurance, while certain vulnerable groups such as the elderly and disabled are excluded from the user fee exemption scheme.

The levels of selected essential health services coverage in ASEAN countries are presented in Table 2. Most of the interventions related to the health MDGs (e.g. vaccination, antenatal care, births attended by skilled health personnel) were available in the ASEAN countries. The coverage of diphtheria tetanus toxoid and pertussis (DTP3) vaccination among 1-year-old children was over 90% in the region, except in Indonesia, Lao PDR, Myanmar, and the Philippines. The coverage of antenatal care for pregnant mothers was also quite high in the region (over 90%), except in Lao PDR, Malaysia, and Myanmar. The proportion of births attended by skilled health personnel was quite low in some countries, such as Lao PDR, the Philippines, and Myanmar. There was wide variation in antiretroviral therapy (ART) coverage among people with HIV eligible for ART, ranging from 17% in Indonesia to 84% in Cambodia. Despite their importance to public health in the region, data on the coverage of services related to non-communicable diseases (NCDs), mental health problems, and injuries are, however, not available. This is a key data gap given the growing burden of NCDs and mental health problems in all countries.

**Table 2:** The coverage of selected essential health services in ASEAN countries

	Diphtheria tetanus toxoid and pertussis (DTP3) coverage among 1 year old (%), 2013^a^	Antenatal care coverage, at least 1 visit (%), latest year^b^	Births attended by skilled health personnel (%), latest year^b^	Children aged < 5 years with Acute Respiratory Infection (ARI) symptoms taken to a health facility (%), latest year^a^	ART coverage among people with HIV eligible for ART according to 2010 guidelines (%), latest year^b^
Brunei	90	100.0 (2011)	100.0 (2011)	No data	No data
Cambodia	92	89.1 (2010)	71.0 (2010)	64.2 (2010)	84 (49–95)
Indonesia	85	93.3 (2007)	79.8 (2010)	65.9 (2007)	17 (12–25)
Lao PDR	87	71.0 (2010)	37.0 (2010)	32.3 (2006)	51 (44–58)
Malaysia	97	83.4 (2010)	98.6 (2010)	No data	42 (33–53)
Myanmar	75	83.1 (2010)	70.6 (2010)	69.3 (2010)	48 (44–54)
Philippines	94	91.1 (2008)	62.2 (2008)	49.8 (2008)	73 (52–94)
Singapore	97	100.0 (2006)	99.7 (2010)	No data	No data
Thailand	99	99.1 (2009)	99.4 (2009)	84.0 (2006)	76 (72–80)
Vietnam	59	93.7 (2010)	91.9 (2011)	73.0 (2011)	58 (32–95)

a World Health Statistics 2014

b WHO Global Health Observatory.

### Political commitments to UHC in ASEAN countries

The political commitments to endorse UHC have at face value been strong in the ASEAN countries. In these countries, some to a greater extent than others, many policies and strategies have been established and implemented to facilitate progress toward UHC. For example, in Thailand, since 2002, the political commitment to universal access to healthcare was emphasized in the National Health Security Act that states that ‘Thai population shall be entitled to a health service with such standards and efficiency’. In Indonesia, in 2004, the Presidential Bill No. 40/2004 on National Social Security System to protect Indonesian citizens from catastrophic household expenditure due to illness and death was enacted. In Cambodia, in 2005, a Master Plan for SHI was adopted, signifying an essential first step toward establishing a unified health protection system. In Vietnam, in 2012, the Prime Minister approved the Master Plan on UHC with a roadmap to achieve universal health insurance (UHI) coverage levels of 70% by 2015 and 80% by 2020, and to reduce OOP payment to 40% by 2020. In Myanmar, in 2012, the Government has endorsed the goal of achieving UHC by 2030 with aims to improve the health status of the poor and vulnerable, especially women and children. In the Philippines, in 2013, the president amended the National Health Insurance Act of 1995 by signing Republic Act 10606 which mandates the government to shoulder the premiums for the insurance of the indigent and informal sectors thus benefiting many Filipinos. Singapore recently announced the expansion of MediShield, a health insurance scheme designed to avert catastrophic OOP expenditure, which currently covers 93% of the population. The expanded program would be named MediShield Life. It will be mandatory with 100% population coverage and a stated aim of reducing co-insurance levels from the current 10–20% to 3–10% (9). In Malaysia, the shape of UHC continues to be debated, with discussions currently centered on whether the country should transition to a SHI model, 1Care, which would allow the insured to access private facilities. Civil society and trade unions have expressed concerns that 1Care will subsidize private providers at the expense of the public, and discussions have since stalled (10, 11). Furthermore, during the 11th ASEAN Health Ministers Meeting hosted by the Thailand Ministry of Public Health in 2012, a joint statement emphasizing five main health topics, including Building UHC, was signed (12). Whilst there does appear to be a political commitment expressed for UHC, in reality it is difficult for policymakers to balance competing interests of the growing for-profit private sector (in most countries) and the moral imperative to ensure equal access to healthcare.

### Major barriers to achieving UHC in ASEAN countries

All the countries in ASEAN are facing several common barriers to achieving UHC, namely 1) financial constraints; 2) supply side constraints; and 3) the ongoing epidemiological transition at different stages, characterized by increasing burdens of NCDs, persisting infectious diseases, and reemerging potentially pandemic infectious diseases.

**Fig. 2:**
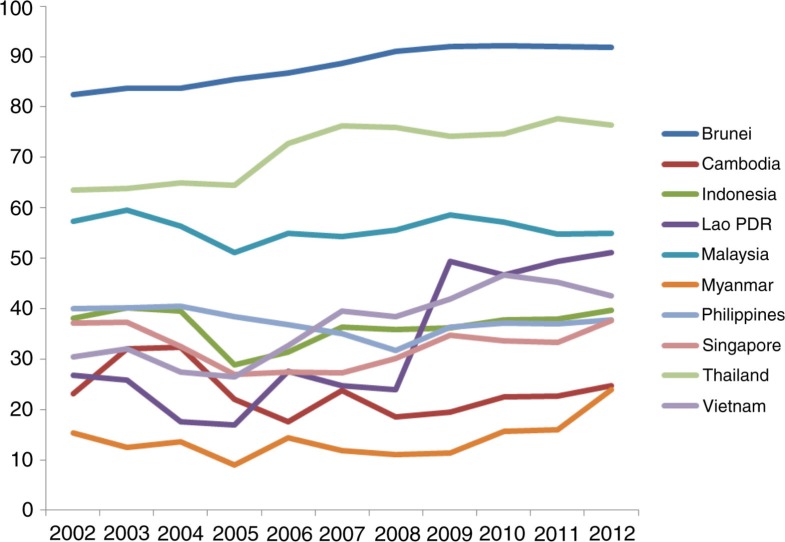
Trends in general government expenditure on health as % of total expenditure on health, 2002–2012.

The key financial constraints are low levels of government spending and overall spending on health. Most countries in the ASEAN region allocated less than 5% of the gross domestic product (GDP) as expenditure on health in 2012, with the exception of Cambodia (5.4%) and Vietnam (6.6%). Government expenditure on health as a percentage of total expenditure of health ranged from 23.9% in Myanmar to 91.8.1% in Brunei. The World Health Organization argues that it is very difficult to achieve UHC if OOP as a percentage of total health spending is equal or greater than 30%, and that the target for UHC could be set at 100% protection from both impoverishing and catastrophic health payments for the population as a whole (2). Government spending on health as a percentage of total government spending varies, from a low of 1.5% in Myanmar to 14.2% in Thailand. Overall, there are higher levels of private spending than public spending on health, with the exception of Brunei and Thailand (see Table 3). Government spending on health as a percentage of total health spending appears to be increasing moderately over time for most countries, except Malaysia, the Philippines, Indonesia and, to some extent, Cambodia (Fig. 2). To ensure UHC, particularly given economic liberalization on the path to AEC, governments should safeguard health budgets and prioritize not only achievement but also maintenance of UHC. This is especially important among ASEAN's middle-income countries, which have arguably been underperforming in terms of social progress relative to countries at similar income levels in other regions (13).

**Table 3:** Financial coverage of UHC in ASEAN countries

	Total expenditure on health as % of GDP, 2012	General government expenditure on health as % of total expenditure on health, 2012	General government expenditure on health as % of total government expenditure, 2012	Social security expenditure on health as % of general government expenditure on health, 2012	OPP as % total expenditure on health, 2012	Incidence of catastrophic medical expenditures (>10% of household spending), 2011
Brunei	2.3	91.8	6.0	No data	8.1	No data
Cambodia	5.4	24.7	6.7	No data	61.7	17.0
Indonesia	3.0	39.6	6.9	17.6	45.3	5.0
Lao PDR	2.9	51.2	6.1	4.9	38.2	9.0
Malaysia	4.0	55.0	5.8	0.9	35.6	2.0
Myanmar	1.8	23.9	1.5	3.0	71.3	No data
Philippines	4.6	37.7	10.3	28.3	52.0	5.0
Singapore	4.7	37.6	11.4	12.7	58.6	No data
Thailand	3.9	76.4	14.2	10.1	13.1	3.5
Vietnam	6.6	42.6	9.5	37.0	48.8	15.1

World Health Statistics 2014.

The share of OOP as a percentage of total health spending in almost all the ASEAN countries, except Brunei and Thailand, was greater than 30% in 2012. As a consequence, the incidence of catastrophic medical expenditures based on the World Bank's methodology (using the cutoff point of 10% of total household spending)^1^ was also high in these countries, especially in Vietnam and Cambodia (Table 3). It should be noted that Singapore through Medisave has a compulsory health savings scheme with correspondingly higher OOP levels since these savings are considered private monies.

Recent analyses based on catastrophic health expenditure and impoverishment revealed that financial coverage in some countries in ASEAN was still modest. The WHO defines households with catastrophic health expenditure as a household with a total OOP health payments equal to or exceeding 40% of a household's capacity to pay. A non-poor household is impoverished by health payments when it becomes poor below the poverty line after paying for health services (14, 15). In Vietnam in 2010, the proportion of households with catastrophic expenditure was 3.9% and the rate of households who were pushed into poverty because of OOPs was 2.5% (16). In Cambodia in 2007, the rates of catastrophic health expenditure and impoverishment were 4.3 and 2.5%, respectively (17). In Lao PDR in 2008, the rates of catastrophic health expenditure and impoverishment were 1.7 and 1.1%, respectively (18). In the Philippines in 2009 the rates of catastrophic health expenditure and impoverishment were 1.2 and 1.0% (19).

The OOP payments as a percentage of total health spending are high (ranging from only 8.1% in Brunei to 71.3% in Myanmar as shown in Table 3) resulting in limited financial protection of vulnerable groups. Government subsidies for health are not sufficiently protecting the poor while reversed subsidies benefit the rich, exacerbating existing inequalities. Across ASEAN countries, funding has been inadequate for investing in infrastructure and installing medical equipment in disadvantaged provincial and district health facilities (6, 20).

For supply side constraints, insufficient healthcare providers and unequal distribution of health professionals have remained significant problems in the ASEAN countries (Table 4). The ratio of doctors to population ranged from two doctors per 10,000 population in Cambodia, Indonesia, and Lao PDR to 14 and 19 doctors per 10,000 population in Brunei and Singapore, respectively. In all the ASEAN countries, there were more nurses and midwives than doctors in the population, except in Vietnam where there were 12 doctors and only 10 nurses/midwives per 10,000 population. In general, there were only less than four pharmacists per 10,000 population in the ASEAN countries, except in Singapore and the Philippines. Recent research showed that all countries in Southeast Asia face problems of mal-distribution of health workers, where rural and remote areas are often understaffed. There is weak coordination between production of health workers and capacity for employment in most countries (21).

**Table 4:** Health workforce in ASEAN countries

	Doctors per 1,000 population, latest year	Nurses and midwives per 1,000 population, latest year	Pharmacists per 1,000 population, latest year
Brunei	1.4 (2010)	7.0 (2010)	0.1 (2010)
Cambodia	0.2 (2008)	0.8 (2008)	0.04 (2008)
Indonesia	0.2 (2012)	1.4 (2012)	0.1 (2012)
Lao PDR	0.2 (2009)	0.8 (2009)	No data
Malaysia	1.2 (2010)	3.3 (2010)	0.4 (2010)
Myanmar	0.5 (2010)	0.9 (2010)	No data
Philippines	1.2 (2004)	6.0 (2004)	0.9 (2011)
Singapore	1.9 (2010)	6.4 (2010)	0.4 (2011)
Thailand	0.3 (2004)	1.5 (2004)	0.1 (2004)
Vietnam	1.2 (2008)	1.0 (2008)	0.3 (2008)

World Health Statistics 2014.

**Fig. 3:**
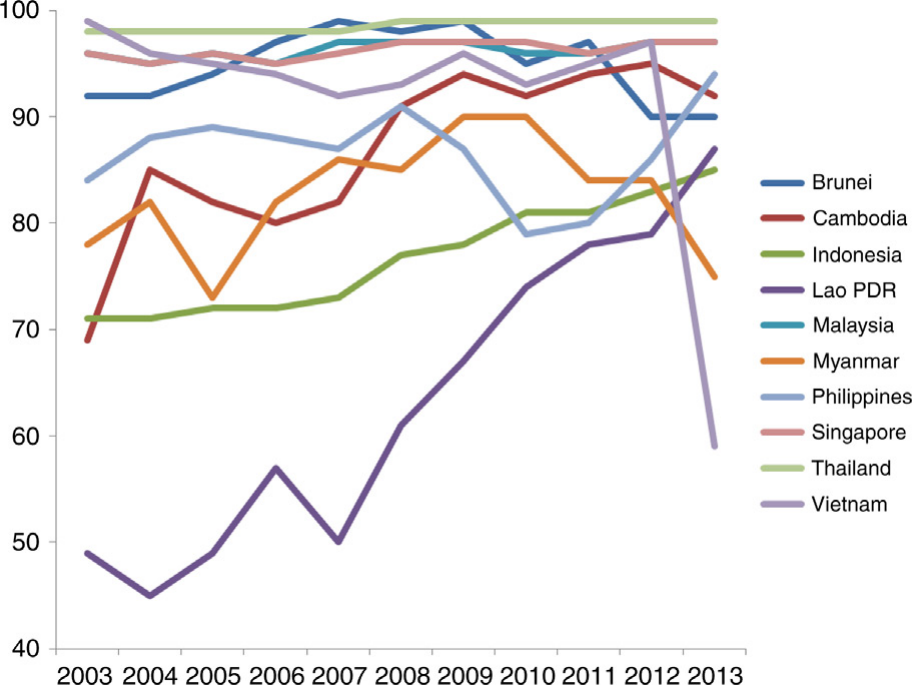
Trends in Diphtheria tetanus toxoid and pertussis (DTP3) immunization coverage among 1-year-olds (%), 2003–2013.

Supply side constraints affect essential health service coverage for UHC, a key indicator of which is immunization rates. As Fig. 3 shows, DTP3 immunization coverage among 1-year-olds has sharply increased in Lao PDR and steadily increased in Indonesia and Cambodia in the past decade. Although rates have fluctuated and declined in the most recent years in Cambodia, along with Myanmar, Brunei, with a drastic drop in DTP3 vaccinations observed in Vietnam last year (from 97% in 2012 to 59% in 2013). Thailand consistently has the highest vaccination rates of 98% and above during this period, followed by Singapore and Malaysia (95% or above), the three countries with the highest health insurance rates in ASEAN.

In terms of epidemiological transition, ASEAN is a hotspot for emerging infectious diseases, including those with pandemic potential. Emerging infectious diseases have exacted heavy public health and economic tolls. Severe acute respiratory syndrome (SARS) rapidly decimated the region's tourist industry. Influenza A (H5N1) has had a profound effect on the poultry industry. The reason why Southeast Asia is at risk from emerging infectious diseases is quite complex. The region is home to dynamic systems in which biological, social, ecological, and technological processes interconnect in ways that enable microbes to exploit new ecological niches (22). At the same time, the ASEAN countries are facing an epidemiological transition with increased morbidity and mortality from NCDs. NCDs are now responsible for 60% of deaths in the region. The problem stems from the ageing of the population, life-style behaviors (tobacco use, alcohol use, unhealthy diet, and inadequate physical activity) and environmental factors. The triple burdens of diseases – persistent and emerging infectious diseases, NCDs, and injuries – pose significant threats to the populations in this region. Disadvantaged populations (such as the poor, people living in rural or remote areas, etc.) are the hardest hit – NCDs account for a high proportion of deaths in ASEAN and particularly as a proportion of deaths in wealthier countries, but they also kill more people in absolute numbers in the less developed countries of ASEAN, with the apparent exception of Cambodia (23). As Table 1 shows, NCD age standardized mortality rates ranged highs of between 680 per 100,000 population in Indonesia and Lao PDR to 708.7 in Myanmar and 720 in the Philippines, compared to much lower rates observed in Singapore (264.8), Vietnam (435.4) and Thailand (449.1). Also important to note are that total mortality rates are relatively low in ASEAN. Deaths from infectious diseases have steadily declined, and currently there are relatively small proportions of older people (between 6 and 15% of those aged 60 or over among ASEAN countries, see Table 1) who die mostly from NCDs. This means that whilst NCDs account for most deaths in ASEAN, age standardized death rates are not too different from other world regions. For example, ASEAN had similar age standardized mortality rates (537.1 per 100,000 population) from NCDs in 2012 as the WHO Europe region (523.9 per 100,000 population) (24). In ASEAN however, a significant proportion of NCD mortality happens prematurely – in 2012, 50.9% of deaths among those aged 70 or younger were caused by NCDs, compared to 31.2% in the WHO Europe region (24). The WHO at the 65th World Health Assembly in 2012 agreed to adopt a global target of 25% reduction in premature mortality from NCDs by 2025 (25), a target that we hope will be vigorously pursued in ASEAN. We refer to NCD data with caution, as few countries in ASEAN have complete causes of death information systems – among them, Singapore is the only country with reliable cause of death certification and coding (6).

ASEAN also faces a demographic transition to a greater share of the elderly as a proportion of total population. In 2015, the percentage of those aged 65 and over is estimated to be 7.1% among ASEAN countries, with the highest proportion of elderly in Singapore (13.7%) and Thailand (12.0%). By 2030, the share of elderly is expected to almost double to a regional average of 12.3% of total population (26). With increasing life expectancies and share of the elderly without commensurate increases in birth rates, population aging has implications for financing UHC and how benefits packages will evolve in the next 20 years, given that healthcare consumption increases with age.

The major challenges and barriers toward UHC can also be contextualized in each of the ASEAN countries. In Cambodia, having a responsive health financing system for both formal and informal sectors is the single biggest barrier to achieving UHC. There is no financial scheme for public servants due to low government salaries and low government spending on health. Furthermore, the concept of health insurance is rather new, with the non-existence of SHI financed by pay roll tax (27). It is estimated that OOP for health was 61.7% in Cambodia in 2012 (27). Women spent more than 10% of their total expenses on health, with the poorest spending 18% and the highest quintiles 14% (28). In Indonesia, insufficient infrastructure (human resources, facilities, and equipment) has hindered progress toward universal coverage for the population, which policymakers aim to achieve by 2019. The ratio of doctors to population in Indonesia is amongst the lowest in Asia (only two for every 10,000 population in 2010, compared to an average of 5.5 per 10,000 population for countries in the WHO South East Asian region). Moreover, the ratio of hospital beds to the population is very low (six beds per 10,000 population against an average of 11 beds per 10,000 population in the WHO South East Asian region) (29). With a large geographical archipelagic area, another huge challenge is to provide equal access to healthcare, including for populations in remote areas and islands of Indonesia. A national health information system (HIS) with unique individual identifiers is currently lacking in Indonesia. A complete and reliable HIS is essential for planning UHC; such a HIS should consider population movement, relevant to ease of obtaining access to healthcare outside of the person's residential area, and it should be possible to link health usage databases from different healthcare providers. In Lao PDR, the level of public expenditure on health, despite efforts to increase it, is still too low, and is currently insufficient to meet the health needs of the population. Geographically scattered and limited population coverage by social protection schemes are both major barriers to accessing care, resulting in a high level of OOP payments and impoverishment; a further government subsidy could help to ease the high burden of OOP payments. There is low utilization of health services because of geographically remote mountainous areas and poverty in Lao PDR. Despite prepayment schemes for four targeted population groups, there are still challenges to implementing these and expanding coverage (the ongoing health finance reform is now addressing this issue). The low quality of care at the health centers and district levels and the constraints of providing a full range of services at the primary care need to be addressed to gain people's confidence and increase utilization of services.

In Myanmar, insufficient and inconsistent investments in health, lack of health workforce and catastrophic health payments are amongst the major barriers to achieving UHC. Though the government has quadrupled its total expenditure on health in recent years, this was merely 2% as a percentage of GDP in 2011 (30). The OOP payments, which decreased from 100% in 2000 to 71.3% in 2011, continue to account for almost all healthcare expenditure (31). In the Philippines, the biggest barrier to achieving UHC is the increase in the coverage of insurance of PhilHealth without commensurate funding increases. In addition to under-funding, the devolution of health services by virtue of the Local Government Code of 1991 resulted in inefficient referral services. Richer Local Government Units tend to better support and maintain their facilities and services thus worsening health inequities between regions.

In Vietnam, almost two thirds of the population is covered by health insurance. However, the coverage of health insurance is still quite low among informal sector workers. Vietnam needs a stronger enforcement mechanism for the formal sector as well as effective measures and support to enroll the informal sector in the scheme. During the past few years, provider payment methods for healthcare costs of national health insurance have changed but fee-for-service payments still dominate the system. In Vietnam, OOP payments as a share of total health expenditure have been always high, ranging from 50% to 70% (32). OOP payments are high and persistent, resulting in limited financial protection for the poor. Meanwhile, government subsidies for health are not sufficiently reaching the poor. Hospital subsidies, in particular, tend to favor the rich, exacerbating existing inequalities (33). Funding is inadequate for investing in infrastructure and installing medical equipment in disadvantaged provincial and district health facilities (34).

High and upper-middle income countries also face barriers in achieving UHC. In Thailand, access to healthcare is limited by the availability of service delivery, particularly health workforce. Despite having extensive networks of healthcare providers, challenges still exist in terms of healthcare provision in remote rural areas where it is difficult to attract and retain qualified health workers. The country has a low doctor-per-population ratio – lower than other countries with a similar economic development level- due to an extended period of limited training capacity. Whilst the ratio of nurses to doctors is high, there is still a large discrepancy in the distribution of doctors and nurses across geographical regions, which is a major challenge for the government. In Singapore, the biggest hurdles are not financial or technical but ideological. The fears of moral hazard leading to over-consumption and over-servicing, as well as eventual financial unsustainability are the main reasons why the government is unprepared to embrace UHC in the spirit of other developed countries. Furthermore, there is a sincere belief that wealth and financial success must translate into better quality of living including healthcare – ‘Work for reward, Reward for work’ is a common mantra espoused by government officials (35). In Malaysia, there are supply side constraints, with significant shortages of health professionals (36). The MOH reported that they were able to fill just 64% of doctors’ posts in 2009, 60% for dentists, and 77% for pharmacists. At the primary healthcare level, only 55% of family medicine specialist posts were filled, as well as 40% of doctors and 85% of nurses. Production capacity has been expanding in public and private medical schools, and the government continues to send medical students abroad on scholarships to receive their training to meet HR needs (36). In Malaysia, a dual healthcare system has emerged, with private services for those who can afford them and public services for the rest, with quality perceived to be higher in the private than in the public sector (37). This results in sicker and poorer patients using public services (36). A barrier to achieving UHC will be to ensure that public sector service quality improves, and service capacity expands (especially in urban areas), to keep up with increased demand. Similar concerns have been voiced out about the emergence of a dual healthcare system in Thailand, where increased demand from the wealthy urban Thai population and to a lesser extent medical tourists for private health services may drive public health workers to the private sector (38).

## Discussion

### ASEAN integration and UHC

The AEC was identified as the goal of regional economic integration by 2015 (39). ASEAN leaders have identified healthcare as a priority sector for region-wide integration. In November 2004, the ASEAN Trade Ministers adopted a roadmap to promote trade in healthcare goods, such as pharmaceuticals and medical equipment. In addition, two service sub-sectors in the healthcare industry have been specifically targeted for progressive liberalization, namely 1) health services, covering hospital services (including psychiatric hospitals), and the services of medical laboratories, ambulances, and residential healthcare other than hospitals; and 2) the services of medical professionals, including medical and dental professionals, midwives, nurses, physiotherapists, and paramedical personnel (40). The opening of healthcare markets promises substantial economic gains but intensifies existing challenges to promote equitable access to healthcare within countries (6). In terms of UHC explicitly, the inaugural ASEAN plus 3 (China, Japan and South Korea) UHC network (convened by ASEAN Health Ministers) meeting in April 2014 indicates that discussions about UHC and ASEAN integration have only recently begun in earnest.

The services sector integration goals of the AEC present the biggest challenges and also the biggest opportunities for the region. Some ASEAN countries such as Singapore and Thailand have already become significant exporters of modern services in sectors such as professional services and information and communication technology (ICT), including business processing outsourcing (BPO), higher education, and health tourism (5). The medical tourism industry in Asia is being catalyzed by the Medical Tourism Association (MTA), a US based non-profit organization that is aiming to set global standards for this industry. Health services tourism has become a substantial industry in Singapore, Thailand, and Malaysia, combining health services for wealthy foreigners with recreational packages to boost consumption of such healthcare services (41). However, each country has adopted different approaches toward medical tourism. In Malaysia, it is an explicit MOH policy to expand high-end private hospital care to cater to medical tourists (36) with the Malaysian Healthcare Travel Council established in 2009 as a promotional arm and subsidiary of the ministry. Of the 35 participating hospitals in 2010, some are corporatized public entities (e.g. National Heart Institute). Doctors in public hospitals with private wards can retain part of the fee for treating private patients, as they can in Singapore's corporatized public system. In Singapore, there is less explicit promotion to attract foreign patients by the MOH, as there has been in Thailand, where medical tourism is delivered and driven mainly by private hospitals (42).

Countries face other challenges related to the opening of healthcare markets. For example, despite the golden opportunity to tap into the large market of the Indonesian population, multinational healthcare companies had shown lukewarm responses to invest in Indonesia. The lack of enthusiasm is mainly due to the restrictions and regulations on foreign investments in the country, such as in its pharmaceutical industry, which was regulated by the Presidential Decree (Perpres) Number 36 in 2010 (43). Multinational healthcare companies are also required to establish local manufacturing facilities to promote knowledge transfer. Amendments to the negative investment list have been signed though by the President of the Republic of Indonesia through Presidential Decree Number 39 enacted on 23 April 2014 (44). This amendment was intended to increase foreign investment in Indonesia in preparation for the AEC. To illustrate some changes in the economic climate, the highest level of capital ownership of multinational pharmaceutical companies has increased from 75 to 85% (45).

Progressive liberalization of services of health professionals poses risks to health equity within and between countries. According to the Mutual Recognition Arrangement (MRA) of the AEC, physicians, nurses, and dentists are among seven selected professional groups that are free to work across member countries (46). Although the financial returns from this strategy seem substantial, issues of equity within UHC have become a concern due to the possibility of health worker flight from poorer regions already struggling to ensure UHC. There is a real risk of undesirable outcomes whereby only the better-off will receive benefits from the liberalization of trade policy in health, either via regional outmigration of health workers or intra-country health worker movement toward private hospitals, which tend to be located in urban areas (6).

Another challenge posed by regional integration to UHC policies is the larger number of migrant workers whose movement will be less restricted following liberalization. Migrant workers are unlikely to be automatically enrolled in national health insurance schemes and thus may not have adequate health service access or benefits (7). Each country must have a clear policy – perhaps an ASEAN-wide policy – that defines adequate healthcare coverage and benefit packages for migrant workers.

### How can UHC be fully achieved in ASEAN countries?

Research and country experiences demonstrate that adopting UHC is primarily a political, rather than a technical issue, with incremental progress achieved over long time periods (47). There is a large role governments can play, although this can take many forms, with the route to UHC being contingent on effective leaders, social movements, salient moral claims about appropriate levels of coverage, as well as economic cycles and policy development in other sectors (48). UHC can be achieved – even among low and middle-income countries – by strengthening the health system, securing sustainable and equitable financing, selecting the right benefit package, and reorganizing domestic health expenditures to be used more efficiently (2, 49–51). There must be explicit political commitment to expanding healthcare coverage and ensuring affordability for healthcare users, as can be observed in policy reforms in Indonesia and Singapore.

There is potential to organize select health services regionally to improve further efficiency. For example, member countries in ASEAN could ‘share’ clinical services intended for rare diseases or conditions such as glycogen storage diseases. In practice, this already happens to some extent – Singapore maintains a sophisticated burns unit which *de facto* serves the region. Singapore is also establishing a proton beam therapy facility which should be affordably priced for appropriate ASEAN patients, perhaps through special government arrangements so that this resource can be well-utilized and made available to a much wider pool of patients. Expanding coverage of good-quality services and ensuring adequate human resources are also important to achieve UHC. As health-financing reform is complex, institutional capacity to generate evidence and inform policy is essential and should be strengthened (20). This aligns with the call of WHO for countries to continue to invest in local research in order to develop a system of UHC tailored to each individual country's situation (3).

For ASEAN countries, UHC should ideally be considered in efforts toward regional economic integration by 2015. Regional cooperation in health systems operations toward UHC must be strengthened in the coming time, especially considering increased population movement between countries. At the same time, regional collaboration in priority issues in global health, such as emerging infectious disease epidemics, disaster preparedness, NCDs and migration, capacity building, and building of health work force across the region is needed. Lessons and experiences in prevention and control of NDC should be shared and replicated among these countries. In face of ASEAN liberalization and in the midst of overall expansion of private health providers and transnational healthcare companies, it is more important than ever that UHC is given explicit priority to safeguard access to health systems particularly among disadvantaged groups.

We regret, given the shortage of data, that we could not provide a complete picture on the situation of UHC in each country as well as across ASEAN. We also did not have sufficient longitudinal data to discuss time trends beyond selected indicators pertaining to UHC and associated factors.

## Conclusions

Immense challenges are facing ASEAN countries in ensuring UHC. The OOP payments are alarmingly high in most ASEAN countries, and countries have been unable to ensure sufficient human resources for health (HRH) and health facilities and their distribution among more disadvantaged provincial and district areas. The triple disease burden and increasing inter and intra country migration implies that flexibility and adaptation by the region's health systems is needed. Despite apparent political commitments to UHC in most countries, actual implementation and action have been understandably slow or delayed, given the enormity of some of these challenges (e.g. integrating SHI schemes and stepwise recruitment to a unified UHC scheme in Indonesia).

In the short-term, we believe that capacity building and technical sharing of expertise on UHC experiences, health systems strengthening (HSS) and health services is both feasible and desirable. In the medium term, mobility of HRH can be leveraged in two ways. First, medical missions of HRH to lower income countries could be expanded to build capacity in that health system – via technical expertise sharing, such as training on medical equipment or new technologies or health service delivery methods. HRH going to higher income countries (on short term training, but also migration) could also share knowledge on delivering health services in less well-resourced settings.

In the medium term, policymakers should consider a policy for free or low-cost emergency health services for short-term ASEAN travelers resulting from accidents or illness accrued in the destination country, and a basic package of health services for labor migrants. If they have not already done so, country MOHs could agree on an Essential Health Package (EHP) of public health interventions and health services that each person should avail of in their home country, as recommended by the WHO. Such EHPs can help promote dialogue on health priorities within countries, as well as improve accountability by monitoring progress toward EHP goals (52). Similarly, MOHs along with relevant ministries, should consider outlining basic safety standards for services and products, such as food and drugs (e.g. permitted additives/ingredients) in ASEAN-wide standards/agreements. Disease surveillance by each country, with timely information sharing during outbreaks, will also contribute to better health in ASEAN.

In the long term, we envisage that social protection could be designed in various ASEAN wide packages – including health insurance and elderly care, making health coverage regional. A regional health fund, into which ASEAN countries contribute based on national income levels, could be used to contribute to disease outbreaks and surveillance. Countries could apply to this fund for proposed UHC or HSS initiatives/structural improvements.

We recognize that some of these proposed actions are occurring within bilateral MOUs and ASEAN MRAs (such as HRH migration), or on an informal basis between countries. However, we believe that ASEAN has potential to formalize some of these actions within an ASEAN-wide framework – these could first be designed as multilateral ASEAN-wide MRAs, before consideration of whether to implement legal frameworks, for example, for a basic package of emergency health services that countries are obliged to provide for short-term ASEAN travelers. We also recognize that implementation capacity differs widely among countries, as well as the ability to enforce policies (e.g. food safety standards). However, with political will and increased investment in public health systems, we believe that ASEAN has significant potential to become a force for better health in the region. Ultimately, we hope that all ASEAN citizens can enjoy higher health and safety standards, comprehensive social protection, and improved health status.
