# Supplementary material for: Special Issue: ASEAN Integration and its Health Implications
Source: Glob Health Action. 2015 Jan 27;8:10.3402/gha.v8.27368. doi: 10.3402/gha.v8.27368 (PMC4309835; doi:10.3402/gha.v8.27368)
Supplement: Related texts

# Sub article: Progress toward universal health coverage in ASEAN

**DOI:** 10.3402/gha.v7.25856

**Published:** 2014-12-03

**Authors:** Hoang Van Minh, Nicola Suyin Pocock, Nathorn Chaiyakunapruk, Chhea Chhorvann, Ha Anh Duc, Piya Hanvoravongchai, Jeremy Lim, Don Eliseo Lucero-Prisno, Nawi Ng, Natalie Phaholyothin, Alay Phonvisay, Kyaw Min Soe, Vanphanom Sychareun

**Affiliations:** 1 Department of Health Economics, Hanoi Medical University, Hanoi, Vietnam; 2 London School of Hygiene and Tropical Medicine, London, UK; 3 School of Pharmacy, Monash University Malaysia, Selangor, Malaysia; 4 Center of Pharmaceutical Outcomes Research, Department of Pharmacy Practice, Faculty of Pharmaceutical Sciences, Naresuan University, Phitsanulok, Thailand; 5 School of Population Health, University of Queensland, Queensland, Australia; 6 National Institute of Public Health, Phnom Penh, Cambodia; 7 Ministry of Health, Hanoi, Vietnam; 8 Faculty of Medicine, Chulalongkorn University, Bangkok, Thailand; 9 Health and Life Sciences Practice, Oliver Wyman, New York, NY, USA; 10 Department of Public Health, Xi'an Jiaotong-Liverpool University, Suzhou, PR China; 11 Faculty of Management and Development Studies, University of the Philippines (Open University), Los Baños, Philippines; 12 Epidemiology and Global Health, Department of Public Health and Clinical Medicine, Faculty of Medicine, Umeå University, Umeå, Sweden; 13 The Rockefeller Foundation, Bangkok, Thailand; 14 National University of Laos, Vientiane, Lao PDR; 15 Faculty of Public Health, Mahidol University, Bangkok, Thailand; 16 University of Health Sciences, Vientiane, Lao PDR

**Keywords:** Universal Health Coverage, integration, ASEAN

## Abstract

**Background:**

The Association of Southeast Asian Nations (ASEAN) is characterized by much diversity in terms of geography, society, economic development, and health outcomes. The health systems as well as healthcare structure and provisions vary considerably. Consequently, the progress toward Universal Health Coverage (UHC) in these countries also varies. This paper aims to describe the progress toward UHC in the ASEAN countries and discuss how regional integration could influence UHC.

**Design:**

Data reported in this paper were obtained from published literature, reports, and gray literature available in the ASEAN countries. We used both online and manual search methods to gather the information and ‘snowball’ further data.

**Results:**

We found that, in general, ASEAN countries have made good progress toward UHC, partly due to relatively sustained political commitments to endorse UHC in these countries. However, all the countries in ASEAN are facing several common barriers to achieving UHC, namely 1) financial constraints, including low levels of overall and government spending on health; 2) supply side constraints, including inadequate numbers and densities of health workers; and 3) the ongoing epidemiological transition at different stages characterized by increasing burdens of non-communicable diseases, persisting infectious diseases, and reemergence of potentially pandemic infectious diseases. The ASEAN Economic Community's (AEC) goal of regional economic integration and a single market by 2015 presents both opportunities and challenges for UHC. Healthcare services have become more available but health and healthcare inequities will likely worsen as better-off citizens of member states might receive more benefits from the liberalization of trade policy in health, either via regional outmigration of health workers or intra-country health worker movement toward private hospitals, which tend to be located in urban areas. For ASEAN countries, UHC should be explicitly considered to mitigate deleterious effects of economic integration. Political commitments to safeguard health budgets and increase health spending will be necessary given liberalization's risks to health equity as well as migration and population aging which will increase demand on health systems. There is potential to organize select health services regionally to improve further efficiency.

**Conclusions:**

We believe that ASEAN has significant potential to become a force for better health in the region. We hope that all ASEAN citizens can enjoy higher health and safety standards, comprehensive social protection, and improved health status. We believe economic and other integration efforts can further these aspirations.

## References

[1] World Health Organization (2013). Positioning health in the post-2015 development agenda.

[2] World Health Organization (2010). The world health report: health systems financing: the path to universal coverage.

[3] World Health Organization (2013). Research for universal health coverage.

[4] United States (1989). Department of State. Bureau of Public A. ASEAN Department of State publication. Background notes series. http://www.state.gov/p/eap/regional/asean/.

[5] ASEAN Integration Monitoring Office, World Bank (2013). ASEAN integration monitoring report.

[6] Chongsuvivatwong V, Phua KH, Yap MT, Pocock NS, Hashim JH, Chhem R (2011). Health and health-care systems in southeast Asia: diversity and transitions. Lancet.

[7] Guinto R, Zuwasti Curran U, Suphanchaimat R, Pocock NS (2014). Universal health coverage in “One ASEAN”: are migrants included?. Glob Health Action.

[8] President of the Republic of Indonesia (2011). The Law of the Republic of Indonesia Number 24/2011 about Social Security Administrative Bodies (Badan Penyelenggara Jaminan Sosial).

[9] Chanel NewsAsia MediShield Life Review Committee unveils recommendations. http://www.channelnewsasia.com/news/singapore/medishield-life-review/1137558.html.

[10] Anuar MK 1Care: what ails Malaysia's healthcare system? The Malaysian Insider; 2012. http://www.themalaysianinsider.com/sideviews/article/1care-what-ails-malaysias-health-care-system-mustafa-k.-anuar/.

[11] Yow HC 1Care roadshow an “afterthought,” says PKR president. The Malaysian Insider; 2012. http://www.themalaysianinsider.com/malaysia/article/1-care-roadshow-an-afterthought-says-pkr-president.

[12] ASEAN. 11th ASEAN health ministers meeting. 2012 http://www.asean.org/news/item/11th-asean-health-ministers-meeting-2.

[13] Phua KH, Yap MT, Schwanke Khilji S, Lee H-PL, Detels R, Gulliford M, Karim QA, Tan CC (2015). Chapter 1.4. Development of public health in economic transition: the middle-income countries. Oxford textbook of global public health.

[14] Xu K, Evans DB, Kawabata K, Zeramdini R, Klavus J, Murray CJ (2003). Household catastrophic health expenditure: a multicountry analysis. Lancet.

[15] World Health Organization (2005). Distribution of health payments and catastrophic expenditures methodology.

[16] Van Minh H, Kim Phuong NT, Saksena P, James CD, Xu K (2013). Financial burden of household out-of pocket health expenditure in Viet Nam: findings from the National Living Standard Survey 2002–2010. Soc Sci Med.

[17] World Health Organization–Regional Office for Western Pacific (2011). The Cambodian demographic health survey and Cambodian socio-economic surveys: analysis report.

[18] World Health Organization–Regional Office for Western Pacific (2011). Financial burden from OOP expenditures and health facility utilization in Lao PDR.

[19] World Health Organization–Regional Office for Western Pacific (2011). Financial burden of health payments in the Philippines.

[20] Tangcharoensathien V, Patcharanarumol W, Ir P, Aljunid SM, Mukti AG, Akkhavong K (2011). Health-financing reforms in Southeast Asia: challenges in achieving universal coverage. Lancet.

[21] Kanchanachitra C, Lindelow M, Johnston T, Hanvoravongchai P, Lorenzo FM, Huong NL (2011). Human resources for health in Southeast Asia: shortages, distributional challenges, and international trade in health services. Lancet.

[22] Coker RJ, Hunter BM, Rudge JW, Liverani M, Hanvoravongchai P (2011). Emerging infectious diseases in Southeast Asia: regional challenges to control. Lancet.

[23] Dans A, Ng N, Varghese C, Tai ES, Firestone R, Bonita R (2011). The rise of chronic non-communicable diseases in Southeast Asia: time for action. Lancet.

[24] World Health Organization (2014). Global health observatory.

[25] World Health Organization (2012). Second report of Committee A, 65th World Health Assembly.

[26] United Nation (2010). World population prospects: the 2010 revision.

[27] World Health Organization (2010). World health statistics.

[28] Chhorvann CH, Vonthanak S, Bajracharya A, Dingle A, Bellows B (2012). Out-of-pocket expenditure among women using reproductive health services at public health facilities in Cambodia.

[29] World Health Organization (2013). World health statistics 2013.

[30] Ministry of Health of Myanmar (2013). Health in Myanmar. The Republic of Union of Myanmar. http://www.moh.gov.mm/file/MYANMAR%20HEALTH%20CARE%20SYSTEM.pdf.

[31] World Health Organization (2010). Global health observatory, Official Development Assistance (ODA) recipient country profiles, 2000–2010. http://www.who.int/gho/governance_aid_effectiveness/countries/en.

[32] Ministry of Health of Viet Nam, Health Partnership Group (2008). Join annual health review 2008.

[33] Somanathan A, Dao HL, Van Tien T (2013). UNICO studies series 24: integrating the poor into universal health coverage in Vietnam.

[34] Ministry of Health, Health Partnership Group (2013). Joint annual health review 2013.

[35] Lim J (2013). Myth or magic: the Singapore healthcare system.

[36] APOHSP (2013). Malaysia health system review. Asia Pacific Observatory on health systems and policies.

[37] Chee HL (2008). Ownership, control, and contention: challenges for the future of healthcare in Malaysia. Soc Sci Med.

[38] Wibulpolprasert S, Pachanee C (2008). Addressing the internal brain drain of medical doctors in Thailand: the story and lesson learned. Global Soc Pol.

[39] Kanithasen P, Jivakanont V, Boonnuch C (2011). AEC 2015: ambitions, expectations and challenges ASEAN's path towards greater economic and financial integration.

[40] Dee P, Corbett J, Umezaki S (2009). Services liberalization toward the ASEAN economic community. Deepening east Asian economic integration.

[41] Arunanondchai J, Fink C (2005). Trade in health services in the ASEAN region.

[42] Pocock NS, Phua KH (2011). Medical tourism and policy implications for health systems: a conceptual framework from a comparative study of Thailand, Singapore and Malaysia. Global Health.

[43] President of the Republic of Indonesia (2010). Presidential Regulation of the Republic of Indonesia Number 36/2010 on list of business fields closed and business fields open with conditions to investment in Indonesia.

[44] President of the Republic of Indonesia (2014). Presidential Regulation of the Republic of Indonesia Number 39/2014 on list of business fields closed and business fields open with conditions to investment in Indonesia.

[45] Indonesian Investment Coordinating Board (2013). Explanation to the Presidential Decree Number 39/2014 on Negative Investment List.

[46] Association of Southeast Asian Nations ASEAN mutual recognition arrangement framework on accountancy services. http://www.asean.org/communities/asean-economic-community/item/asean-mutual-recognition-arrangement-framework-on-accountancy-services-3.

[47] Stuckler D, Feigl A, Basu S, McKee M (2012). The political economy of universal health coverage.

[48] Savedoff WD, Smith AL (2011). Achieving universal health coverage: learning from Chile, Japan, Malaysia and Sweden.

[49] Attaran A, Capron AM (2014). Universal health coverage and health laws. Lancet.

[50] Marten R, McIntyre D, Travassos C, Shishkin S, Longde W, Reddy S (2014). An assessment of progress towards universal health coverage in Brazil, Russia, India, China, and South Africa (BRICS). Lancet.

[51] O'Connell T, Rasanathan K, Chopra M (2014). What does universal health coverage mean?. Lancet.

[52] World Health Organization (2008). Essential health packages: what are they for? What do they change?.

# Sub article: Innovations in non-communicable diseases management in ASEAN: a case series

**DOI:** 10.3402/gha.v7.25110

**Published:** 2014-09-22

**Authors:** Jeremy Lim, Melissa M. H. Chan, Fatimah Z. Alsagoff, Duc Ha

**Affiliations:** 1 Health and Life Sciences Practice, Asia Pacific Region, Oliver Wyman, Singapore; 2 Vriens and Partners, Singapore; 3 Secretarial and Coordination Division, Cabinet Office, Ministry of Health, Hanoi, Vietnam

**Keywords:** non-communicable diseases, innovations, health care, developing countries, ASEAN

## Abstract

**Background:**

Non-communicable diseases (NCDs) are reaching epidemic proportions worldwide and present an unprecedented challenge to economic and social development globally. In Southeast Asia, the challenges are exacerbated by vastly differing levels of health systems development and funding availability. In addressing the burden of NCDs, ASEAN nations need to fundamentally re-examine how health care services are structured and delivered and discover new models as undiscerning application of models from other geographies with different cultures and resources will be problematic.

**Objective:**

We sought to examine cases of innovation and identify critical success factors in NCD management in ASEAN.

**Design:**

A qualitative design, focusing on in-depth interviews and site visits to explore the meanings and perceptions of participants regarding innovations in NCD against the backdrop of the overall context of delivering health care within the country's context was adopted.

**Results:**

In total 12 case studies in six ASEAN countries were analysed. Primary interventions accounted for five of the total cases, whereas secondary interventions comprised four, and tertiary interventions three. Five core themes contributing to successful innovation for NCD management were identified. They include: 1) encourage better outcomes through leadership and support, 2) strengthen inter-disciplinary partnership, 3) community ownership is key, 4) recognise the needs of the people and what appeals to them, and 5) raise awareness through capacity building and increasing health literacy.

**Conclusions:**

Innovation is vital in enabling ASEAN nations to successfully address the growing crisis of NCDs. More of the same or wholesale transfers of developed world models will be ineffective and lead to financially unsustainable programmes or programmes lacking appropriate human capital. The case studies have demonstrated the transformative impact of innovation and identified key factors in successful implementation. Beyond pilot success, the bigger challenge is scaling up. Medical technologies are crucial but insufficient; passionate and engaged leaders and communities enabled by enlightened policy makers and funding agencies matter more.

Non-communicable diseases (NCDs) have been described as presenting an unprecedented challenge to economic and social development globally (1, 2). The latest projections from the World Health Organization (WHO) suggest 57 million deaths occurred globally in 2008, of which 36 million (63%) were the result of NCDs. Despite the immense burden of disease, NCDs, defined here as cardiovascular diseases, cancers, chronic respiratory conditions, and diabetes (3), nonetheless present a public health opportunity to intervene, not just in disease treatment but at all stages of disease progression. It is estimated that 80% of NCDs are preventable with appropriate diet and lifestyle choices (4) and large-scale clinical trials such as the UK PDS (5) highlight that good control of NCDs can have substantial effect on the incidence of downstream complications.

ASEAN which comprises mainly emerging economies faces difficulties in combating NCDs related to the rapid increase in disease incidence and inadequate health systems preparation for these increases. For example, in Indonesia, 7.6 million people are living with diabetes, while another 12.6 million have pre-diabetes (6). By 2030, the number of people with diabetes in Indonesia will hit 11.8 million (7). This equates to a 6% annual growth that exceeds the country's overall population growth (8). Moreover, fewer than half of those with diabetes are aware of their condition (6) and likely many are poorly informed of the risk factors and the appropriate behaviours to mitigate the risk. Those who remain in rural areas will have the greatest need for treatment from a health care system strained by demand for resources (9). Developing countries in ASEAN also have to deal with the continuing challenges arising from communicable diseases. Unlike in the developed world, where infectious diseases such as malaria have largely been eradicated or controlled, developing countries face an unfortunate double burden of managing infectious diseases and the NCD epidemic, which is due in part to the rapidly changing lifestyles and diets over one generation (10). Hence, a comprehensive and coherent NCD programme has to be implemented at the same time as infectious diseases are being fought (11).

## The need for NCD health care innovation in ASEAN

According to the WHO, without intervention, deaths from NCD are predicted to increase by 15% between 2010 and 2020 (1). The biggest increase would occur in the African, Eastern Mediterranean, and Southeast Asian regions (1). Fortunately, most premature deaths from heart disease, stroke, and diabetes can be averted with behavioural modifications and pharmaceutical interventions (12).

Chronic diseases tend to be of long duration, can be progressively disabling, and are often life-threatening, requiring a much different model of health care than what most ASEAN countries have now (13). Our health care systems have been geared towards treating acute illnesses, which usually have an identifiable single cause and readily apparent cure. On the other hand, chronic diseases by their very nature require holistic interventions in lifestyle and behaviour, education in self-management, and complex life-long treatments. Hence, more of the same interventions based on episodic care models will not do (14).

ASEAN needs to fundamentally re-examine how health care services are structured and delivered while also being aware of the resource availability of well-resourced compared to low-resourced countries. Constrained in various ways including the shortage of skilled staff in remote settings, poor facilities, and scarcity of essential equipment and medicines, health care providers in low-resourced countries are compelled to innovate and seek practical solutions that may never have been thought of in the well-resourced developed world.

It is in this light that the ASEAN NCD Network sought to find examples of innovation and success factors of these innovations in NCD management in ASEAN. This was undertaken as part of a White Paper by the ASEAN NCD Network exploring the use of innovations in ASEAN. The ASEAN NCD Network is an informal grouping of health care experts from around the region working together to address the issue of NCDs. The network aims to promote regional collaboration and cross-country sharing of ideas and best practices within the ASEAN region.

## Objectives of the study

The paper does not set out to be a comprehensive plan for addressing the challenges of NCDs. It concentrates on innovation and what has worked in the ASEAN context. It highlights the awareness of the cultural context and sensitivity that is needed in introducing innovations in Asia. While the word ‘innovation’ often brings to mind highly complex and extremely costly technological advancements, ‘innovation’ in this context is not defined as highly complex technological advancements but encompasses also solutions that make use of cheap and readily available resources that are contextually relevant in the respective setting.

## Methods

### Design

We used a qualitative design, focusing on in-depth interviews to explore the meanings and perceptions of participants regarding innovations in NCD against the backdrop of the overall context of delivering health care within the country's context.

### Participants and setting

A purposive sampling was used to identify the cases for innovation with two distinct strategies used to generate a diverse pool of innovation cases. First, we circulated an innovation identification checklist (Table 1) to members of the ASEAN NCD Network Steering Committee (Table 2), personnel from the various ASEAN Ministries or Departments of Health, and global NGOs for recommendations. This was then followed up by a call to the respective informants to gather more details of the project leader information and to also verify that the participants met the checklist criteria.

**Table 1:** Checklist for innovation identification

Categories		Tick where applicable	Comments (if any)
1	Target one of four NCD focus areas	[ ]	_______
	Cardiovascular diseases	[ ]	_______
	Diabetes	[ ]	_______
	Chronic lung diseases	[ ]	_______
	Cancer	[ ]	_______
2	Types of prevention	[ ]	_______
	Primary	[ ]	_______
	Secondary	[ ]	_______
	Tertiary	[ ]	_______
3	Occurs within Southeast Asia	[ ]	_______
	Yes	[ ]	_______
	No	[ ]	_______
4	Programme duration ≥18 months	[ ]	_______
	Yes	[ ]	_______
	No	[ ]	_______
5	Reasonably high-quality data available	[ ]	_______
	Yes	[ ]	_______
	No	[ ]	_______

**Table 2:** Steering committee members

Dr Jeremy Lim, Partner, Head of Health and Life Sciences Practice, Asia Pacific, Oliver Wyman
Dr Tai E Shyong, Head, Division of Endocrinology, National University Hospital, Singapore
Dr Daphne Khoo, Chief Medical Officer, Fortis Healthcare International, Singapore
Dr Nazeli Hamzah, President, Malaysian Association of Adolescent Health, Malaysia
Dr Wasista Budiwaluyo, Secretary General, Indonesian Hospital Association (PERSI), Indonesia
Dr Ha Anh Duc, Senior Researcher, Division of Non-Communicable Diseases, Institute of Population, Health and Development (PHAD), Vietnam
Dr Ly Ngoc Ha, Director, Development Center for Public Health, Vietnam
The late Dr Alberto G. Romualdez Jr., President, Culion Foundation, Philippines
Dr Antonio Dans, Professor, College of Medicine, University of the Philippines Manila, Philippines
Dr Pura Angela Wee, Associate Director, Zuellig Center for Asian Business Transformation, Asian Institute of Management (AIM), Philippines

The process yielded 12 cases in six ASEAN countries, comprising Thailand, Singapore, the Philippines, Indonesia, Vietnam, and Malaysia.

### Data collection

The interviews were conducted from January 2013 through December 2013 by a multidisciplinary team with varied backgrounds and experience, including: clinical medicine, clinical epidemiology, and public health.

For each of the innovations identified for more study, the research team conducted preliminary phone or email interviews with the project leaders to ascertain the suitability of the case study based on the criteria checklist.

A question guide was developed for the study trips where the researchers would personally meet and interview them about their innovations. The topic guide covered open-ended questions seeking to understand their innovations, personal motivations, and challenges faced.

The research team also travelled to the innovation sites to observe the innovations in action and to speak to health care providers, volunteers, and patients on the ground for their own perspectives on the programmes.

### Analysis and validation

A thematic analysis approach was adopted. The analysis sought to identify associations between themes and report patterns (15). All interviews were recorded through note taking. The data was examined manually and the themes were identified by highlighting the hard copies. The thematic analysis was further tested during discussions amongst the study team.

## Results

### Basic characteristics of the case studies of innovation

In total 12 case studies on innovation were found in six ASEAN countries, categorised into three interventions: primary, secondary, and tertiary. Primary interventions accounted for five of the total cases, whereas secondary interventions comprised of four, and tertiary interventions three (Tables 3–6).

**Table 3:** Classification of case studies yielded from different countries

	Level of prevention			
		
	Primary	Secondary	Tertiary	Total
Thailand	1	0	1	2
Singapore	1	1	0	2
The Philippines	2	0	0	2
Indonesia	0	1	2	3
Vietnam	0	1	0	1
Malaysia	1	1	0	2

**Table 4:** Summary of five innovations on NCD primary prevention in ASEAN^a^

**Innovation 1. Lifestyle Building Programme, Theptarin Hospital, Thailand**	
Focus	Diabetes and related chronic diseases
Innovation	First hospital in Thailand to introduce integrated diabetes care team comprising of an endocrinologist, a diabetes nurse educator, and a dietitian; introduction of new care models through a paradigm shift
	Chronic diseases through healthy living and behaviour modification
Strategy	Education, research, and patient education to introduce the concept of holistic team-based care for patients with diabetes
Key finding	Commitment to public education and emphasis on professional capacity building
**Innovation 2. Strategies to Promote Healthy Eating Behaviours, Singapore Health Promotion Board**	
Focus	Obesity
Innovation	Encouraging the people of Singapore to eat healthy by using novel tools adopted by market research companies, and engaging local food manufacturers and hawkers to develop and sell healthier foods though the board's ‘Ask For’ program
Aim	Find innovative ways to promote health and healthy eating by empowering Singaporeans to request and opt to choose healthier foods when eating out
Strategy	Advancing health promotion initiatives upstream by engaging local food manufacturers to co-develop healthier foods
Key finding	Upstreaming of education, health promotion initiatives and interventions as shown by successful health programs promotion at the national level
**Innovation 3. Enactment of a ‘Sin Tax’ on Tobacco Products, Department of Health, Philippines**	
Focus	Tobacco use
Innovation	Utilisation of strategic means and political will by pro-sin tax reformers to push forth their agenda; signed into law in December 2012 after 16 years of review and revisions
Aim	Introduction of the Sin Tax law in the Philippines so as to reduce tobacco consumption
Strategy	Building a critical mass base for supporting efforts of tobacco control advocates and pushing of the tobacco tax law through engaging lawmakers’ support and civil society participation
Key finding	The confluence of a popular administration with a firm political will and closely coordinated actions between the policy holders and the tobacco reform advocates from the civil society is crucial in mobilising supporters from the government and people to bring about change
**Innovation 4. ‘Gotong Royong’ Behavioural Change Model, Melaka State Health Department, Ministry of Health, Malaysia**	
Focus	Obesity and tobacco use
Innovation	Using the concept of ‘gotong royong’ – the spirit of volunteerism, selflessness, and working together for the benefit of the community; villagers of Malacca were given RM4,000 (approx.US$1,250) per village to print materials, train volunteers in health education, nutrition, and exercise
Aim	Engaging villagers in Malacca to take charge of their health
Strategy	Identifying village heads who are keen in leading the project and providing support and education to train the volunteers
Key finding	Sense of ownership, community empowerment, and a spirit of volunteerism were important factors in making the community health programme a success
**Innovation 5. Package of Essential Non-Communicable Disease (PEN) Implementation, Department of Health, Philippines**	
Focus	Tobacco use and diet
Innovation	Implementation of the WHO PEN programme with modifications on strategies and techniques to fit the needs of the people of Pateros, Philippines
Aim	Reduction of premature deaths due to NCDs in Pateros
Strategy	Soliciting support for the implementation of the PEN project by engaging key stakeholders in the health care system and policy makers in Pateros
Key finding	The anecdotal evidence of increased awareness regarding NCDs as well as the programs is encouraging; the Department of Health has adopted the PEN guidelines and is considering scaling the program to the whole country

a Complete case studies can be found at: www.healthspace.asia.

**Table 5:** Summary of four innovations on NCD secondary prevention in ASEAN^a^

**Innovation 1. Visual Inspection with Acetic Acid (VIA), Female Cancer Programme, Indonesia**	
Focus	Cervical cancer
Innovation	Working within the constraints of low-resource settings, health care providers are compelled to innovate and seek practical solutions for cervical cancer screening
Aim	Introduction of cervical screening to the provinces in Indonesia
Strategy	Introducing creative low-cost cervical cancer screening treatment to the provinces in Indonesia as well as the significance of socialisation and education of the women and husbands on the need for cervical screening and early treatment
Key finding	Apart from the solutions, a can-do attitude, inventiveness, and true community spirit are needed to make a programme successful
**Innovation 2. Earlier Presentation of Cancer Patients for Definitive Diagnosis and Treatment, Department of Radiotherapy, Oncology, & Palliative Care, Sarawak General Hospital, Malaysia**	
Focus	Breast and nasopharyngeal cancer
Innovation	Creative means of utilising the medical assistants (MAs) and nurses in the rural clinics to diagnose the patients are adopted for better outcomes
Aim	Education of community nurses and MAs in the rural areas on early symptoms of cancer (breast, cervical, and nasopharyngeal)
Strategy	Training the MAs and nurses facilitating referral system from the rural area to the hospital
Key finding	Localisation of health education materials helps the rural villagers to understand and connect
**Innovation 3. Community-Based Hypertension Management, Vietnam National Heart Institute**	
Focus	Hypertension
Innovation	Strategic utilisation of human capital to ensure the success of the programme
Aim	Introduction of community-based hypertension management programme in Vietnam
Strategy	Engaging in an community-based lifestyle study
Key finding	The success of the program is contributed by 1) successful engagement of the whole community, 2) support from committed local authorities and medical expertise, and 3) training of committed health care workers
**Innovation 4. Tele-Health Interventions to Chronic Patients, Eastern Health Alliance, Singapore**	
Focus	Diabetes
Innovation	Development of an alternative care delivery methods for chronic patients post discharge using tele-health system used by the Eastern Health Alliance for its Health Management Unit
Aim	Introduce innovative ways to keep the aging population healthy and prevent them from hospital admission
Strategy	Identifying discharged chronic patients through the Relationship Management Program; the system monitors the health of the patient and alerts the health care provider if the patient's test results showed worsening condition, visiting for consult or missing a medical appointment will be monitored as well
Key finding	Alternative methods are needed to explore care delivery for patients while also being aware of the patient's needs during their illness journey

a Complete case studies can be found at: www.healthspace.asia.

**Table 6:** Summary of three innovations on NCD tertiary prevention in ASEAN^a^

**Innovation 1. Digital Retinal Photography for Diabetics in Rural Communities, The Center of Excellence (COE) for Retina Diseases, Rajavithi Hospital & Institute of Medical Research and Technology Assessment (IMRTA), Department of Medical Services, Ministry of Public Health, Thailand**	
Focus	Diabetic retinopathy (DR)
Innovation	Alternative solutions are explored to bring DR screening into the villages so that more people could benefit
Aim	Bringing eye care to the villagers so that villagers could have DR screening access
Strategy	Identify committed village volunteers who are not medically trained to learn and perform the DR screening
Key finding	A strong political will is needed to implement the Diabetic Blindness Prevention project to the community
**Innovation 2. Improving Care of Pediatric Patients with Diabetes, Indonesian Pediatric Endocrinology Chapter, Indonesian Pediatric Society (IDAI) and Indonesian Association of Families with Diabetes Mellitus Children (IKADAR)**	
Focus	Diabetes
Innovation	The project lead skilfully capitalised on data to seek funds and also utilised multi-prong approaches for outreach programmes to create awareness
Aim	Establish a comprehensive diabetes management programme for children with type 1 diabetes
Strategy	Identifying the childhood diabetes problem with data, reach out to the funding bodies to present the case and seek funding; building capacity among health care providers and engaging stakeholders and family in awareness programmes
Key finding	Education and awareness creation for the patients and convincing stakeholders are key to successful programme implementation
**Innovation 3. Community Diabetes Strengthening, Indonesia**	
Focus	Diabetes
Innovation	Flexibility and stakeholder engagement strategies to introduce the programme were highlights of the initiative
Aim	Improve the capacity of preventing, detecting, and treating diabetes to reduce the burden of diabetes in Indonesia
Strategy	Establishing diabetes management systems in hospitals and primary health centres in the community
Key finding	Results showed a 15% increase in diabetes education provided in the provincial hospitals and over 20% increase in the puskesmas. A total of 1,237 health professions in all were trained in diabetes management; establishment of specific diabetes clinics

a Complete case studies can be found at: www.healthspace.asia.

### Analysis

Five themes which contribute to successful innovation for NCD management were identified based on the themes gathered from the case studies.

### Theme 1: Encourage better outcomes through leadership and support

The importance of leadership to the change management process is underscored by the fact that change, by definition, requires creating a new system and then institutionalising the new approaches. Change leadership is needed to be the driving force to instill visions and processes that fuel large-scale transformation (16).

The case studies illustrate that successful programs at least initially depend on the people and the leaders who are driving and implementing the programs. Similar leadership traits were identified in the people who drove the programmes.

These programme leaders were adept at:

1. Identifying both internal and external strengths and weaknesses of themselves and the programmes
2. Influencing and mobilising team members and partners to complement their skills
3. Utilising their strong networks to support their vision

Above all, passion, vision, and a strong sense of mission to improve the lives of people were the driving force behind innovation in NCD management in these case studies.

Table 7 illustrates examples of how better outcomes are encouraged through leadership and support.

**Table 7:** How better outcomes are encouraged through leadership and support

Barriers or challenges	Solution	Leadership traits exhibited
**Example: Innovation 1 – Thailand**		
The ‘Innovator’ lacked experience and had no concrete plan in disease detection programmes Limited health care staff: The Thai government focus then was on treatment and prevention of acute diseases, rather than on chronic diseases such as diabetes. Hence, medical manpower was trained accordingly	Partner like-minded clinicians and hospital managers Serve as President of the Nutrition Association and Organising Chairman of the International Congress of Nutrition (2009) to inspire the establishment of a master's and an undergraduate programme in Food and Dietetics at Thai universities	• The ‘innovator’ was able to identify strengths and weaknesses • Mobilise the right people to complement strengths and weaknesses • Utilise networks to advocate for change
**Example: Innovation 7 – Malaysia**		
Nurses and medical assistants (MAs) were unable to refer patients with cancer symptoms to the district hospital due to the referral system limitations Doctors in the district hospital do not see patients who were referred by the nurses and MAs. This affects the nurses and MAs morale to refer patients	Change the workflow process in partnership with the State Health Department to allow nurses and MAs to refer patients directly Nurses and MAs would alert the programme leader directly so that action could be taken	• Utilise networks to advocate for change • Leadership commitment and support

### Theme 2: Strengthen inter-disciplinary partnerships

Collaboration is based on the understanding that individual and community well-being is determined as much by social, environmental, and economic systems as by health care provision. Hence, the promotion and maintenance of health does not belong to one professional group or sector. Partnership constructs are widely advocated in order to implement strategies to influence the wider determinants of health and health inequalities, and thus secure population health improvement. Partnerships are seen as important tools for improving public health outcomes because shared intelligence of both ‘soft’ and ‘hard’ information improves the understanding of the needs and wants of the local communities. It also provides opportunities for shared learning (17). Yet, it is also recognised that partnership is neither easy nor a panacea for tackling health issues. Central to partnership working is an awareness of different working cultures and the roles of individuals and professionals can influence outcomes of collaboration.

For participants to believe that the partnership is beneficial, a clear defined vision of what needs to be achieved has to be established (18, 19). Hence, taking time to identify shared values through open discussions is deemed to be the first step in partnership. For example, to solicit support for the implementation of the Package of Essential NCD interventions in Pateros, the project leader engaged key stakeholders in the health care system and policy makers in Pateros. The consultation workshop involving the key stakeholders helped to establish a defined goal. The mayor of Pateros was also advised on the growing economic and social burden of NCDs in Pateros, paving the way for securing the necessary funding.

In the community diabetes innovation in Indonesia, the project leader also recognised that strengthening government partnership is also important hence one of the key strategies is to continually advocate at all levels of the government.

### Theme 3: Community ownership is key

Community ownership contributes to effective initiatives. NCD prevention and treatment generally requires patients to alter entrenched behaviours. Medical professionals, however, have limited bandwidth and resources to consistently monitor patients. As such, community members play an important role in serving as doctors’ conduits to support and promote lifestyles that mitigate NCDs.

In Malacca, whole communities are educated on caloric counting and ways to prepare healthier foods. The village chief is an example of a health ambassador who serves to inspire the entire village. The community in Kampung Pantai Peringgit comes together to exercise by walking while at the same time inspecting and removing potential breeding sites for the *Aedes* mosquito, which transmits dengue to humans.

The importance of community ownership was also recognised by Dr. Paisan Ruamviboonsuk, project leader in ‘Digital Retinal Photography for Diabetics in Rural Communities’ who brought eye care to people in the rural areas of Thailand. He articulated, ‘One of the most important elements that made this initiative a success was giving an opportunity to local community health care personnel in rural areas, who were not trained in ophthalmology at all, to solve a problem in public health ophthalmology for their own people. They run their own project and they can do it successfully’.

### Theme 4: Recognise the needs of the people and what appeals to them

To garner support and sustain results, programmes must incentivise and appeal to all stakeholders involved.

Examining the success of the Vietnamese blood pressure control program, the consistent monitoring and measurements of progress were important factors that helped to sustain the program. Dr. Quang Ngoc Nguyen, cardiologist at the Vietnam National Heart Institute and project leader, emphasised, ‘We had funding for only a few years to pay for the high blood pressure medicines. After that, the villagers had to pay for the medicines out of their own pockets, and they did! And this was because we measured. They could see for themselves the difference taking medicines made to their blood pressure and this encouraged them to continue’. The element that contributed to the program's success was measuring and associating health and well-being to the villagers’ adherence to medication.

In another example of a national initiative to modify eating behaviours and encourage Singaporeans to eat healthily, the Health Promotion Board (HPB) worked with hawkers to launch Singapore's ‘Healthy Hawker’ initiatzive (20). To garner participation from the hawkers, HPB structured and marketed the program so that hawkers could see tangible benefits. It was reported that hawkers had a tripling of sales of dishes made with brown rice and wholegrain noodles and more importantly, earnings went up by at least 10% thus encouraging hawkers to support the programme.

However, different communities are in varying stages of willingness and readiness to change. Hence, community engagement and needs identification plays an important role. To address this, Dr. Noraryana in Malacca presented village leaders with a menu of options to enable leaders to select and tailor programs to suit their own community's needs and interests.

### Theme 5: Raise awareness through capacity building and increasing health literacy

One recurrent theme throughout the case studies is the importance of education for capacity building among the health care professionals and patient education for patients.

In the example of introducing early cancer detection in Sarawak, the team was unable to reach out to the local villages to educate them on early cancer detection. However once they identified the maximum point of leverage – the medical assistants (MAs) and nurses who were highly regarded by the villagers, the outreach efforts were much more successful. The MAs and nurses lived in the villages hence they knew the villagers well and were able to establish rapport with them. By engaging and training the MAs and nurses who had a desire to upgrade their medical skills and knowledge, the programme's outreach expanded.

Increasing health literacy and awareness of disease conditions among patients was also a key strategy through most of the case studies. Patient education materials and methods were contextualised and customised to fit the rural villagers’ local norms. To raise awareness of diabetes among stakeholders and the general public, mass media campaigns were also conducted in Indonesia's diabetes program. Dr. Aman Pulungan, the project leader, had appeared on ‘live’ television speaking about childhood diabetes.

## Discussion

Four points are worth noting. First, there is no dearth of innovations in NCD management occurring throughout ASEAN countries, and there are myriad opportunities for ASEAN countries to learn from each other.

Second, a common observation from the cases is the frugality of innovation. For example, Vietnam's blood pressure control program works out to US$ 0.06 per villager; Malaysia's cancer control program costs US$ 9,250 a year. The Indonesian effort to improve diabetes health care delivery across eight sites in Java, Sumatra, and Sulawesi needed less than half a million US dollars from the World Diabetes Foundation (21). In ‘See and Treat’ programs to address cervical cancer, inexpensive household vinegar is the key ingredient.

However, more important than frugality is the third noteworthy issue which is the needed flexibility in deploying funds. We had identified earlier the importance of community engagement and local solutions, and this necessitates on-site adaptation to meet local needs. Hence, although funds can be secured nationally or at state-level, their use is local. One commonly expressed frustration was the restriction and conditions on the use of funding. For example, programmes received funds that allowed for commercially printing materials but not for purchasing computers, printers, and paper which would have been more cost-effective. We spoke to many leaders who highlighted examples of having to ‘work around’ funders’ conditions and obtaining funding from varied sources so that collectively, the programme's resource needs could be adequately covered.

The success of NCD management programmes depends on the empowerment of the local community and local leadership. The practical reality is that different communities have different ways of executing projects and hence adopt different ways of using funds. Funding agencies need to recognise this and strike that balance between appropriate governance and accountability and enabling recipients to work efficiently and expeditiously. Our case series suggests that the best funding agencies, whether governmental or non-governmental, are the ones that are actively involved in the program with an on-site presence and quick and easy approval processes for any variations from the submitted proposal.

It is critical that practitioners have the freedom to allocate these resources flexibly to avoid waste and inefficiencies. This is in some ways akin to start-up investing where investors bring funding and short- and long-term objectives but leave the management team otherwise largely alone to execute. The requirement to deploy resources as flexibly as possible is necessary given the rapidly changing market dynamic. In innovating in health programs, the process of executing often brings new learning. The opportunities to incorporate new learning into the program are important. Budget allocations and interim milestones should hence be flexible enough to allow for evolution and flexibility.

### Strengths of the study

This study presents innovative health promotion efforts towards NCD management. It highlights the need to be culturally aware when engaging stakeholders and the patient groups. More importantly, this study highlights a common trait identified throughout the whole case series – political will and strategic engagement of key stakeholders are the key factors for successful programmes implementation.

### Limitations of the study

Although we have cast the net widely in terms of participants, the sample may not represent the full spectrum of innovations in ASEAN. We acknowledge that there are challenges in inter-sectoral collaboration, and we do not attempt to simplify the complex nature of stakeholder engagement and collaboration. However, our main aim was to identify common themes that allow for successful innovations in the respective ASEAN countries.

## Conclusions

Innovation is vital in enabling ASEAN nations to successfully address the growing crisis of NCDs. More of the same or wholesale transfers of developed world models will lead to financially unsustainable programmes or programmes lacking appropriate human capital. The case studies have demonstrated the transformative impact of innovation and identified key factors in successful implementation. Beyond pilot success, the bigger challenge is growing from ‘seed to scale’. Medical technologies are crucial but insufficient; passionate and engaged leaders and communities enabled by enlightened policy makers and funding agencies matter.

In order to tackle the growing challenge of NCDs, we propose three recommendations to support innovations in NCD management:

### Build an Asian databank of innovations for addressing the growing burden of diseases arising from NCD

The innovation databank should become a first-call resource for ideas and solutions for ASEAN countries to counter NCD. Evidence on the effectiveness, value-for-money, and likely impact of the innovations could be made available online. Information should be free and publicly accessible. Innovations that are unable to show impact and sustainability will also be included as this could present as learning points and future references.

### Introduce platforms for innovators to engage with each other, funders, and policy makers

Providing platforms for networking would enable like-minded innovators to share resources and practices and to provide support for further innovations. The inclusion of funders and policy makers would also enhance mutual understanding and enable decision makers to understand the needs and challenges of the people on the ground when implementing programs.

### Support the NCD management innovators in bringing from SEED to SCALE and building up sustainable business models

The SEED to SCALE theory of social change promulgated by Future Generations (22) suggests that the most available and sustainable approach to scaling up successful pilots lies in redirecting how people apply their energies. The most valuable resource is not money but the energy and enthusiasm of the community which is created and reinforced by autonomy and ownership. Hence, supporting the innovators to determine their own priorities and focus on practical solutions would enhance momentum for change and solutions to fit local circumstances.

## References

[1] World Health Organization (2011). Global status report on non-communicable diseases 2010.

[2] Bloom DE, Cafiero ET, Jané-Llopis E, Abrahams-Gessel S, Bloom LR, Fathima S (2011). The global economic burden of non-communicable diseases.

[3] General Assembly of the United Nations (2011). Resolution adopted by the General Assembly on 19 September 2011: political declaration of the high-level meeting of the general assembly on the prevention and control of non-communicable diseases.

[4] Willett WC, Koplan JP, Nugent R, Dusenbury C, Puska P, Gaziano TA, Jamison DT, Breman JG, Measham AR (2006). Prevention of chronic disease by means of diet and lifestyle changes. Disease control priorities in developing countries.

[5] Yates T, Haffner SM, Schulte PJ, Thomas L, Huffman KM, Bales CW (2014). Association between change in daily ambulatory activity and cardiovascular events in people with impaired glucose tolerance (NAVIGATOR trial): a cohort analysis. Lancet.

[6] IDF diabetes atlas 5th edition 2012 update (2012). http://www.idf.org/sites/default/files/5E_IDFAtlasPoster_2012_EN.pdf.

[7] IDF diabetes atlas 6th edition (2013). http://www.idf.org/diabetesatlas.

[8] World economic outlook database, April 2012 (2012). http://www.imf.org/external/pubs/ft/weo/2012/01/weodata/weoselgr.aspx.

[9] Where economics and health meet: changing diabetes in Indonesia (2013). http://www.novonordisk.com/images/Sustainability/PDFs/Blueprint-for-change-Indonesia--52383_Korr19.pdf.

[10] Fact sheet: noncommunicable diseases http://www.who.int/mediacentre/factsheets/fs355/en/.

[11] Dans A, Ng N, Varghese C, Tai ES, Firestone R, Bonita R (2011). The rise of chronic non-communicable diseases in southeast Asia: time for action. Lancet.

[12] Frieden T (2014). CDC: 5 things cause two-thirds of U.S. deaths.

[13] Sharma J (2013). Chronic disease management in the South-East Asia Region: a need to do more. WHO South East Asia J Public Health.

[14] Keynote speech by Mr Gan Kim Yong, Minister for Health, at The Economist's “Healthcare In Asia 2012” Conference, 28 March 2012, at The Fullerton Hotel Ballroom (2012). http://www.moh.gov.sg/content/moh_web/home/pressRoom/speeches_d/2012/keynote_speech_by_Minister_Gan_Economists_Healthcare_in_Asia_2012_Conference_28Mar2012.html.

[15] Braun V, Clarke V (2006). Using thematic analysis in psychology. Qual Res Psychol.

[16] Kotter International (2011). Change management vs. change leadership – what's the difference? Forbes.

[17] Naidoo J, Wills J (2010). Developing practice for public health and health promotion.

[18] Delaney FG (1994). Muddling through the middle ground: theoretical concerns in intersectoral collaboration and health promotion. Health Promot Int.

[19] Plamping D, Gordon P, Pratt J (2000). Modernising the NHS: practical partnerships for health and local authorities. Br Med J.

[20] Hui PC (2011). Healthy hawker centre proves a hit.

[21] World Diabetes Foundation (2012). Prevention and control of diabetes WDF08-328 Indonesia.

[22] Future generations: empowering communities to shape their futures http://www.future.org/applied-research/process-change.

# Sub article: Universal health coverage in ‘One ASEAN’: are migrants included?

**DOI:** 10.3402/gha.v8.25749

**Published:** 2015-01-24

**Authors:** Ramon Lorenzo Luis R. Guinto, Ufara Zuwasti Curran, Rapeepong Suphanchaimat, Nicola S. Pocock

**Affiliations:** 1 Universal Health Care Study Group, University of the Philippines Manila, Manila, Philippines; 2 Nuffield Department of Population Health, University of Oxford, Oxford, United Kingdom; 3 International Health Policy Programme, Ministry of Public Health, Nonthaburi, Thailand; 4 Department of Global Health & Development, London School of Hygiene and Tropical Medicine, London, United Kingdom

**Keywords:** migrant health, migrant workers, ASEAN, Southeast Asia, universal health coverage, health financing

## Abstract

**Background:**

As the Association of South East Asian Nations (ASEAN) gears toward full regional integration by 2015, the cross-border mobility of workers and citizens at large is expected to further intensify in the coming years. While ASEAN member countries have already signed the Declaration on the Protection and Promotion of the Rights of Migrant Workers, the health rights of migrants still need to be addressed, especially with ongoing universal health coverage (UHC) reforms in most ASEAN countries. This paper seeks to examine the inclusion of migrants in the UHC systems of five ASEAN countries which exhibit diverse migration profiles and are currently undergoing varying stages of UHC development.

**Design:**

A scoping review of current migration trends and policies as well as ongoing UHC developments and migrant inclusion in UHC in Indonesia, Malaysia, Philippines, Singapore, and Thailand was conducted.

**Results:**

In general, all five countries, whether receiving or sending, have schemes that cover migrants to varying extents. Thailand even allows undocumented migrants to opt into its Compulsory Migrant Health Insurance scheme, while Malaysia and Singapore are still yet to consider including migrants in their government-run UHC systems. In terms of predominantly sending countries, the Philippines's social health insurance provides outbound migrants with portable insurance yet with limited benefits, while Indonesia still needs to strengthen the implementation of its compulsory migrant insurance which has a health insurance component. Overall, the five ASEAN countries continue to face implementation challenges, and will need to improve on their UHC design in order to ensure genuine inclusion of migrants, including undocumented migrants. However, such reforms will require strong political decisions from agencies outside the health sector that govern migration and labor policies. Furthermore, countries must engage in multilateral and bilateral dialogue as they redefine UHC beyond the basis of citizenship and reimagine UHC systems that transcend national borders.

**Conclusions:**

By enhancing migrant coverage, ASEAN countries can make UHC systems truly ‘universal’. Migrant inclusion in UHC is a human rights imperative, and it is in ASEAN's best interest to protect the health of migrants as it pursues the path toward collective social progress and regional economic prosperity.

Guided by the mantra “One Vision, One Identity, One Community,” the 10 member countries of the Association of Southeast Asian Nations (ASEAN) are now gearing toward full regional economic integration by 2015. As laid out in the ASEAN Economic Community Blueprint, the goal is to transform Southeast Asia into ‘a single market and production base, a highly competitive economic region, a region of equitable economic development, and a region fully integrated into the global economy’ which will allow free flow of goods, services, investment, capital, and skilled labor (1). Given these developments, a further increase in population mobility within the region can be expected in the coming years. For example, the regional bloc has developed ‘Mutual Recognition Arrangements’ that seek to harmonize professional qualification standards, regulations, and procedures across ASEAN member states to facilitate the freer movement and employment of qualified and certified personnel such as doctors, nurses, and dentists (2).

Migration, however, is not a new challenge for the ASEAN region. For the past three decades, Southeast Asia has already become one of the world's most dynamic regions, with a huge volume of migrant workers moving both within the region and between ASEAN and the rest of the world (3). In addition to inter- and intra-regional labor migration, other migration trends have been observed in Southeast Asia, such as undocumented or irregular migration (4) and human trafficking, especially of women and children, for forced labor and the sex industry, which reveal migration's most shameful face (5–7). On the contrary, the region's visa-free policy for ASEAN citizens facilitated the high flux of tourists and other types of temporary migrants from one ASEAN country to another (8). More tourists from other regions are also expected to enter ASEAN's premises once the plan to issue a common visa, similar to the European Union's Schengen visa, is implemented (9).

## Health, well-being, and rights of migrants in ASEAN

Unfortunately, while much attention, including in academic literature, has been devoted to the economic benefits and risks of intra-regional labor migration as well as the social costs of irregular migration and human trafficking within ASEAN, the health and well-being of migrants themselves still remain to be examined (10). For decades, the discourse about health and migration has merely focused on issues pertaining to infectious disease spread and border control measures (11), a narrow view that ignores the individual migrant's well-being and dignity.

Various international declarations and policy instruments have already underscored that health is a fundamental human right that should be enjoyed by all people, including migrants (12–14). In particular, the International Convention on the Protection of the Rights of All Migrant Workers and Members of their Families also emphasized migrants’ right to ‘receive any medical care that is urgently required for the preservation of their life or the avoidance of irreparable harm to their health … on the basis of equality of treatment with nationals of the State concerned’ (15). The 2008 World Health Assembly Resolution 61.17 also urged countries to ‘promote migrant-sensitive health policies’ and to ‘devise mechanisms for improving the health of all populations, including migrants’ (16). Finally, the health of migrants was also featured in the World Migration Report 2013 published by the International Organization for Migration. It is the first-ever report of its kind that focused on migrant well-being, thereby placing the migrant at the center of migration discourse (17).

In Southeast Asia, several efforts are also under way to build momentum around the issue of migrant rights and welfare. In 2007, the ASEAN member countries signed the Declaration on the Protection and Promotion of the Rights of Migrant Workers, which laid down the obligations of sending and receiving states in promoting the fundamental rights and dignity of migrant workers and their families (18). Protecting migrants’ rights was also identified as a strategic objective under the ASEAN Socio-Cultural Community (ASCC) Blueprint (19). Unfortunately, neither of the said regional instruments explicitly mentioned Migrants' right to health or health-related obligations of ASEAN member states toward migrant workers and other people on the move.

Now more than ever, addressing migrant health is necessary, as health problems faced by migrants have become increasingly glaring in recent years. For example, HIV–AIDS has been a major concern among migrants in Southeast Asia (20), particularly among migrant workers entering Thailand (21, 22) and ‘Overseas Filipino Workers’ returning or even deported back to the Philippines (23, 24). In addition, limited access to healthcare among migrants has also been featured in recent regional dialogues organized by various intergovernmental organizations (25). Unfortunately, little information is known about other health vulnerabilities commonly experienced by migrants such as occupational hazards, injuries, and chronic non-communicable diseases.

## Worldwide momentum for universal health coverage – are migrants included?

While migration continues to shape the health of Southeast Asians, the global health community is rallying around the goal of achieving universal health coverage (UHC), which is defined by the World Health Organization (WHO) as providing all people with access to needed health services without incurring financial hardship (26). UHC has already been achieved by most developed countries, in particular for their own citizens, and is now being pursued by being pursued by almost one hundred countries (27), including members of ASEAN (28), with varying levels and speed of progress. Furthermore, UHC is now being advocated by the global health community as an intrinsic component of the health goal for the post-2015 development agenda (29). Today, most countries already have or are establishing pre-payment and risk-pooling systems that combine tax-based financing with premium-based social health insurance and veer away from inefficient and expensive ‘fee-for-service’ payment models, all aimed to reduce out-of-pocket expenditures and prevent impoverishment among households (30).

With this timely campaign toward UHC spreading across the world and particularly within the ASEAN region, and given the background of increasing international migration as described earlier, it is legitimate to ask the question, “Are migrants included in UHC in ‘One ASEAN’?” This paper therefore seeks to explore the nexus between migration and UHC in the ASEAN context, and in particular to examine the nature and level of inclusion of migrants in the UHC schemes of various ASEAN countries.

While diverse frameworks have been developed to build a common understanding of UHC, this paper uses the classic UHC cube introduced by the WHO as a guiding framework for analysis (31). The question of whether migrants are considered, enrolled, and covered falls at large under the first dimension of UHC which is population coverage, represented by the x-axis of the cube (Fig. 1).

**Fig. 1:** Three dimensions to consider when moving toward universal health coverage, with emphasis on migrant population coverage. Adapted from Ref. (31).

Nevertheless, this paper attempts to also tackle the other two dimensions – benefit coverage (termed ‘services’ in Fig. 1), which pertains to the range of healthcare services (promotive, preventive, curative, rehabilitative, palliative) that are provided and paid for by the UHC system; and the level of financial protection (termed ‘direct costs’ in Fig. 1), which pertains to the proportion of costs of services covered by the financing scheme. While population coverage is usually given priority first, the two other dimensions are also important for migrants – ideally, in a UHC system, they should at least be enjoying the same basic benefits as well as some level of financial protection (through reduction of cost-sharing resulting in out-of-pocket payments [OPPs]) that are accorded to non-migrants in the countries of origin and destination alike. Furthermore, ideally, migrant-relevant health services such as medical screening and packages for travel- and occupation-related conditions should also be included in the range of benefits covered.

## Methodology

Since migration is a broad term, this paper only focused on international migrants, particularly international labor migrants and undocumented or irregular migrants (In this paper, the two terms are used interchangeably.). Internal migrants, particularly internally-displaced persons as a result of natural calamities or conflicts, are therefore not included in this paper. Nevertheless, UHC reforms should also consider internal migration to ensure people's access to healthcare anywhere within a country's borders (i.e. portability of health benefits).

For the purpose of this analysis, five out of the 10 ASEAN countries were selected – Indonesia, Malaysia, Philippines, Singapore, and Thailand. While a major consideration for the selection is the familiarity with and interest in these identified countries among the authors, these countries also best represent the entire Southeast Asian region in terms of both migration trends and UHC status. Two countries – Indonesia and Philippines – are predominantly sending countries, while Malaysia, Singapore, and Thailand are major destinations for migrant workers. Furthermore, the five countries are at varying stages in the evolution of UHC.

As this topic is a new area of policy and research, a scoping review approach was adopted. Relevant literature, including grey literature such as government policy documents and reports, media articles, as well as publications made by international institutions, published from 2000 to 2014 was reviewed, with the exception of UN resolutions and national laws enacted before 2000. Key terms such as ‘UHC’, ‘health insurance’, ‘Southeast Asia’, ‘migrant’, as well as the names of the five countries were used to search for references in Google, Google Scholar, and PubMed. Grey literature published in the native languages of Indonesia, Malaysia, and Thailand were also searched and provided by authors who are familiar with migration and UHC issues in those countries. Reference sections of retrieved articles were also checked for other relevant sources that were not captured by the aforementioned search engines.

Latest comparable migration- and UHC-related data were compiled and analyzed manually. Current migration trends, such as migrant stocks and flows, and migrant policies and issues in each country were briefly described. This was followed by discussions about ongoing developments in the UHC projects of the five study countries as well as the migrant health-related features of these systems, with emphasis on the three dimensions of the WHO UHC cube when applicable. Gaps, challenges, and opportunities for mainstreaming migrant health into UHC were then identified.

## Results

### Migration trends and policies in ASEAN

Table 1 summarizes the diverse migration profiles among the five ASEAN countries. Among the receiving countries, Thailand has the largest absolute number of in-migrants, followed by Malaysia and Singapore. However, as a percentage of population, migrants make up almost half of Singapore's total population, compared to only 8.3% in Malaysia and 5.6% in Thailand. It should be noted that, while this paper focuses on inclusion of migrant workers and undocumented migrants, these values also include foreign permanent residents (especially in the case of Singapore) as well as refugees and other migrant categories.

**Table 1:** Migration trends in five ASEAN countries

Parameter	Description	Indonesia	Malaysia	Philippines	Singapore	Thailand	Sources of data
General trend	Depends on the percentage out- or in-migration of total population	Sending	Receiving	Sending	Receiving	Receiving	
Out-migration	Stock estimate of citizens overseas (most recent update available) Includes permanent, temporary, and irregular migrants; tourists not included	2,992,550–6,000,000 in 2013	Estimated at 1 million in 2010	10,489,628 as of December 2012 in 218 countries and territories	192,300 in 2011	1,006,051 as of beginning of 2010	Indonesia: (32) Malaysia: World Bank. Malaysia Economic Monitor: Brain Drain. Kuala Lumpur: World Bank Malaysia; April 2011. Available from: http://www-wds.worldbank.org/external/default/WDSContentServer/WDSP/IB/2011/05/02/000356161_20110502023920/Rendered/PDF/614830WP0malay10Box358348B01PUBLIC1.pdf [cited 10 May 2014] Philippines: Commission on Filipinos Overseas. Stock Estimate of Overseas Filipinos (as of December 2012). Manila: CFO; 2012. Available from: http://www.cfo.gov.ph/images/stories/pdf/StockEstimate2012.pdf [cited 10 May 2014] Singapore: National Population and Talent Division of the Prime Minister's Office, Singapore Department of Statistics, Ministry of Home Affairs, and Immigration & Checkpoints Authority. Population in Brief 2011. Singapore: September 2011 Thailand: Huguet JW, Chamratrithirong A. Thailand Migration Report 2011 – Migration for development in Thailand: Overview and tools for policymakers. Bangkok: IOM Thailand; 2011
	As a percentage of total population	1.24–2.49	3.54	11.23	3.79	1.52	Total population data (2010 estimates) from the United Nations, Department of Economic and Social Affairs, Population Division (2012). World population prospects: the 2012 revision. Available from: http://esa.un.org/unpd/wpp/Excel-Data/population.htm [cited 24 June 2014]
Outward labor migration	Number of deployed workers or skilled migrants overseas in a given year (most recent estimate)	512,168 deployed in 2013	Approximately 330,000 skilled migrants in 2010	1,802,031 deployed in 2012	No information	147,623 deployed in 2011	Indonesia: National Agency for the Protection and Placement of Indonesian Migrant Workers (BNP2TKI). Available from: https://docs.google.com/file/d/0B9zVxTquSWwdQnUwVFlreHI0Y0NaT29JSDBFVnpOS3l1ZkJZ/edit Malaysia: World Bank. Malaysia Economic Monitor: Brain Drain. Kuala Lumpur: World Bank Malaysia; April 2011. Available from: http://www-wds.worldbank.org/external/default/WDSContentServer/WDSP/IB/2011/05/02/000356161_20110502023920/Rendered/PDF/614830WP0malay10Box358348B01PUBLIC1.pdf [cited 10 May 2014] Philippines: (44)
							Thailand: Ministry of Labor, Department of Employment, (2011) as cited in (45)
In-migration	International migrant stock at mid-year, 2013 Includes migrant workers, undocumented migrants, refugees, asylum-seekers, students, and other groups of foreign nationals residing in the country (B) Foreign-born (C) Foreign citizens (R) includes refugees	295,433 (C,R)	2,469,173 (B,R)	213,150 in 2013 (C,R)	2,323,252 (B)	3,721,735 (B,R)	(42)

When it comes to the sending countries, the Philippines reported the largest migrant stock overseas, with nearly 11% of its total population living or working outside of the country. The total includes permanent, temporary, as well as irregular migrants. However, Indonesia is facing difficulties in tracking their migrant flow and arriving at more precise estimates (32) hence the wide estimated range of 3–6 million Indonesians abroad, which nevertheless still reflects a considerably huge out-migration.

Among the three main receiving countries, demand for migrant labor remains high. In Singapore and to an extent, in Malaysia, migrants span the entire skill spectrum, with high-skilled migrants in knowledge industries at one end, and low-skilled migrants concentrated in sectors such as construction, manufacturing, the marine industry, domestic work (house help) and the service sector at the other. Because of the language requirement, there are arguably fewer high-skilled migrants in Thailand; however, its economy remains highly dependent on low-skilled labor, which contribute an estimated 7–10% of the total value of industry, and 4–5% that of agriculture (33).

Despite continuing high demand for migrant labor, all three receiving countries use restrictive policies to varying extents to discourage the use of migrant labor and to prevent permanent settlement of migrant workers. In Singapore for example, employers must pay worker levies to the government (higher for low-skilled than high-skilled workers) as well as SGD 5,000 (USD 4,010) in security bonds (money is returned upon repatriation of worker), and must adhere to sector-specific dependency ceilings. For instance, construction companies are required to hire at least one local worker for every seven foreign workers (34). ‘Foreign workers’ (as opposed to ‘foreign talents’ which refer to highly-skilled workers) are usually hired on short-term contracts (1–2 years).

In countries with large numbers of irregular migrants such as Thailand and Malaysia, intermittent crackdowns, raids on migrant workplaces, and deportations are common, particularly during times of economic or political crises (35). ASEAN countries also deploy migrant labor policy as a foreign policy tool. For example, Indonesia and Cambodia impose periodic bans on sending domestic workers (i.e. house helpers) to Malaysia in response to poor treatment by employers.

From a sending country's perspective, the Philippines had a long history as a sending country especially since the 1970s when labor migration became a centerpiece program in order to address massive unemployment. In 2013 alone, remittances sent by OFWs to families left behind in the Philippines totaled at nearly USD 22.9 billion or 8.4% of the country's gross domestic product (GDP) (36).

Cognizant of the importance of OFWs in Philippine society, the country has through the years developed a sophisticated suite of policies and programs designed to advance their rights and welfare (37), including minimum employment standards for compliance by foreign employers; a social security system that OFWs may register into – the Overseas Workers Welfare Administration (OWWA), considered the first and biggest migrant welfare fund in the world (38); and an extensive network of labor attaches deployed to Philippine embassies and consulates in receiving countries mandated to provide necessary assistance, to name a few. Furthermore, the country has been lauded for its extensive use of bilateral labor agreements that lay down obligations of both the Philippines and the receiving country in order to facilitate the orderly deployment of OFWs as well as to ensure migrant protection (39).

Today, Indonesia, the other major sending country in ASEAN, is also beginning to institute similar policies to protect its citizens overseas. For example, the National Agency for the Placement and Protection of Indonesian Migrant Workers (BNP2TKI) was established in 2006 to oversee the deployment of labor migrants and provide direct welfare and protective services. However, irregular migration remains a huge challenge especially for Indonesia. For example, BNP2TKI reported that in the period of 2006–2012, there were about 4 million Indonesian migrant workers overseas, while the number of undocumented Indonesian migrant workers was estimated to be two to four times higher (40). It was even suggested that the Indonesia–Malaysia migration corridor could be the second largest in the world, surpassed only by that between Mexico and the United States (41).

In terms of intra-regional migration within ASEAN, Malaysia remains the top destination for Indonesians (35% of its overseas citizens) (42) and Filipinos (686,547 deployed as of 2012) (43), while Singapore ranks third in terms of countries with newly hired and re-hired OFWs from the Philippines in 2012 (44). Nonetheless, the Philippines still sends more OFWs to the Middle East than to its neighboring Southeast Asian countries. Meanwhile, as of 2012, roughly 2.5 million of Thailand's low skilled migrants hailed from neighboring countries Cambodia, Laos, and Myanmar, with 1.5 million comprising of either family members of registered migrant workers or undocumented workers (45).

### Migrant inclusion in UHC among ASEAN countries

#### Receiving countries

Among receiving countries, Thailand's government spends comparatively more as a percentage of total health expenditure (75.5%) than Malaysia (55.2%) and Singapore (31%), and has lower OPPs than either country (13.5%) (Table 2). Nevertheless, all three countries have either claimed or been reported to have achieved UHC according to their respective definitions, which in general pertains to healthcare coverage at least for their own citizens (46–48). Thailand and Malaysia adopts a predominantly tax-based financing model, with those employed covered through payroll taxes while the rest of the population, including the poor and the informal sector, through general taxation. Malaysia however has long been considering to shift from a tax-financed system to a social health insurance (49), which could either be an opportunity or further threat to migrant inclusion in UHC if this aspect is not addressed early in the discourse. On the contrary, Singapore's UHC system is financed through medical savings, taxes, and premiums collected through its voluntary scheme for catastrophic illnesses, as described later.

**Table 2:** Health financing among the five ASEAN countries

	OPP as% total expenditure on health, 2012^a^	Total expenditure on health as% of GDP, 2011^b^	General government expenditure on health as% of total expenditure on health, 2011^b^
Indonesia	45.3	2.7	34.1
Malaysia	35.6	3.8	55.2
Philippines	52	4.1	33.3
Singapore	58.6	4.6	31
Thailand	13.1	4.1	75.5

a World Bank (2014). The World Bank DataBank. Available from: http://databank.worldbank.org/data/home.aspx [cited 5 July 2014].

b World Health Organization (2014). Global Health Observatory. Available from: http://www.who.int/gho/en/ [cited 3 August 2014].

#### Thailand

Thailand's National Health Act of 2002 (50) mandated that all Thai citizens not covered by existing schemes for civil servants (Civil Servant Medical Benefit Scheme or CSMBS) and formal private sector employees (Social Security Scheme or SSS) are entitled to the Universal Coverage Scheme (UCS); today, UCS covers approximately 75% of the total population. Legal migrants in the formal sector are also covered under the SSS. In addition, since 2001, the Compulsory Migrant Health Insurance (CMHI) has been enrolling migrant workers upon conduct of pre-employment health screening. Unlike the UCS, which is under the National Health Security Office, CMHI is administered directly by the Ministry of Public Health. Under the CMHI, health benefits, including outpatient and inpatient care, are linked to the hospital where the migrant was registered and screened (51). Irregular migrants are also allowed to opt into the CMHI, and an insurance package for migrant children up to age 7 years is available with annual fees of THB 365 (USD 12) (52).

There are however limitations to the CHMI. First, in August 2013, the annual premium, which is paid in advance by the employer and then later deducted from the migrant's wage, was raised from THB 1,300 to 2,200 (from USD 40 to 68). If they wish to continue their membership in the scheme, migrant workers also have to pay an additional THB 600 (USD 19) for the compulsory health screening every year, as the law is not clear on who should defray the examination cost. Moreover, in general, the benefits are still not the same as the ones made available to Thai citizens under UCS; examples of services not provided for CMHI members but guaranteed to UCS members include as therapy for psychotic and substance abuse patients, dental prosthesis, hemodialysis, and kidney transplant (53). CMHI is generally not portable as it is linked to the province and hospital where the migrant originally registered. Migrant coverage still needs to be expanded, since, as of August 2013, the scheme has only registered 66,000 out of the 1 million targeted beneficiaries (54).

Additionally, while the CMHI policy is quite open for undocumented migrants to be registered to the scheme, some hospitals may request various documents that can deter undocumented migrants from enrolling in the scheme. For instance, in Samut Prakan and Chiang Mai, a recent study found that hospitals often require at least one official document to purchase insurance, such as a temporary legitimate residence permit (also known as Tor Ror 38/1), a passport, or, for undocumented migrants without these identity documents, an approved document from the employer such as their house registration (55). Reasons for such documentary requirements include concerns that the card will be rented out to other migrant workers (as had occurred in some cases) and a general unease among some providers about selling the card to undocumented migrants – who themselves may not feel confident to approach the hospital and purchase the card (55).

#### Malaysia

Since 2011, Malaysia has been implementing the *Skim Perlindungan Insurans Kesihatan Pekerja Asing* (SPIKPA; Hospitalization and Surgical Scheme for Foreign Workers), the mandatory private medical coverage scheme for all foreign workers. Enforced by the Ministry of Health, all foreign workers are required to take up this compulsory scheme from one of 28 insurance providers (56), with a premium of MYR 120 (USD 34) and a total coverage of MYR 10,000 (USD 2,778) for use of any health services in the public health system (57). The scheme is mandatory for foreign workers in all sectors (premiums paid by employer or worker), but it is optional for house helpers and plantation workers (whose premiums must still be paid by employers). In Sabah however the rules differ slightly – plantation owners are required to pay for the premium for their workers. By the end of 2011, an estimated 1.2–1.4 million out of 1.8 million registered migrant workers were covered by the SPIKPA scheme (58, 59). Unlike Thailand's CHMI, SPIKPA does not allow irregular migrants to opt into the scheme.

In addition, migrant workers are also covered under the Workmen's Compensation Act (WCA), which provides for lump sum payments for death and disability and stipulates regulations on employer payment of medical costs. The scheme however has been criticized as the maximum liability of employers, which is MYR 300 (USD 84) for surgical ward treatment and MYR 250 (USD 70) for operation charges, as well as the maximum compensation, which amounts to only MYR 23,000 (USD 6,388) in the case of permanent disablement, are hugely insufficient (60).

Migrant workers are however not eligible to enroll in another worker protection scheme, the Social Security Organization (SOCSO), which provides insurance coverage against job-related injuries and disabilities, workplace accidents, occupational diseases and death (61). Another scheme, the Employee Provident Fund (EPF), requires mandatory monthly contribution among Malaysian formal sector workers which provides disbursements for medical care; however, registration remains optional for migrant workers. As a default, private sector employers may also opt for private insurance schemes for their workers.

Among those not covered (partially or fully) by any of these financing schemes, Malaysian citizens pay MYR 1 (USD 0.28) for every consultation with a general practitioner and MYR 5 (USD 1.39) with a specialist. However, non-citizens are charged MYR 15 (USD 4.2) and MYR 60 (USD 17), respectively (62). Surgeries and other specialist services incur higher OPPs, although Malaysian patients in third class wards at public hospitals can only be billed up to a maximum of MYR 500 (USD 139), or half of that for those aged 60 or over (58, 63). However, for foreigners including migrant workers, the minimum deposit is MYR 400 (USD 111.1) for third class wards and MYR 800 (USD 222.2) for surgical cases. While the WCA stipulates that medical charges above the maximum employer liability should come from public funds, in practice some migrant workers are left with excessive bills that they cannot pay.

In order to access public health services, migrant workers need to produce their private insurance card at the hospital registration counter, omitting the need for upfront cash payments (58). In practice however, many employers keep migrant's passports and health cards, making it difficult for them to seek treatment (64). Nonetheless, the public sector technically cannot refuse emergency care to those who cannot pay via prepaid insurance or OPPs, including irregular migrants.

#### Singapore

Finally, Singapore's healthcare financing framework adopts what is called “multiple layers of protection,” which combines heavy government subsidies for acute hospital care with contributory schemes for primary care and catastrophic illnesses (popularly known as 3M) (65, 66). The first ‘M’ refers to Medisave, a compulsory individual medical savings account to which employers and employees contribute, and which can be used to pay for medical expenses. MediShield, the second financing mechanism, is a low-cost and voluntary medical insurance scheme for catastrophic expenditures, and is typically used for larger medical bills. Currently, Singaporean citizens are allowed to opt out of this publicly-administered risk pool should they prefer to avail of private insurance. Recent changes to MediShield will make it a compulsory scheme with lifelong protection, making it more progressive (risk pooling across the entire population) than its predecessor. The third scheme, MediFund, constitutes the final safety net for needy Singaporean patients. It is a medical endowment fund set up by the government to cover those who cannot pay medical bills, covering those with lower incomes but also those who earn more but face large bills relative to their income.

Migrants in Singapore, whether high- or low-skilled workers, are not included under the 3M scheme, hence the private coverage options made available for them. Employers of high-skilled workers (registered under the ‘Employment Pass’ permit) are not required to purchase medical insurance, while for Work Permit holders (low-skilled foreign workers) or S-Pass holders (semi-skilled foreign workers), employers are required to purchase a minimum private medical insurance coverage of SGD 15,000 (USD 11,193) per year for inpatient care and surgery, a limit which is easily breached in face of large medical bills. Worse, foreign workers are ineligible for medical subsidies; in excess of what the medical insurance package can cover, employers are then required to bear the full costs of medical treatment.

Additionally, in cases of disputes on medical expenses arising from work-related illness or injury, the Work Injury Compensation Act (WICA) provides for a process through which claims can be made for medical leave wages, medical expenses and lump sum compensation for permanent incapacity or death. Under WICA, employers are mandated to provide their migrant employees with private insurance that is sufficient to meet payouts in case of work-related illness or injury. The amount for medical expenses compensation has been capped at SGD 30,000 (USD 22,386) (67), which can also be easily breached due to high cost of services (68). In addition, because of lengthy WICA claims processing, foreign workers lose income. While they are entitled to medical leave wages, very few actually receive them. Many see their work permit cancelled and are issued with a special pass which allows them to stay in Singapore while their claim is being processed, but not to take up employment. Many workers are thus forced to turn to nongovernmental organizations for support or to take illegal employment. For more serious cases, it is not uncommon for employers to quickly repatriate workers in order to avoid paying for medical treatment (69).

#### Sending countries

As Table 2 shows, the sending countries have similar levels of government spending on health as a proportion of total health spending, although Indonesia spends less on health as a proportion of GDP (2.7%) as compared to the Philippines (4.1%). While conventionally, destination countries are expected to ensure access to healthcare for migrants that they receive, source countries have also begun providing basic health coverage for their outgoing migrants.

#### Philippines

Since 1995, the Philippines’ National Health Insurance Program or PhilHealth has been striving to achieve its mandate of ensuring financial risk protection for all Filipino citizens (70). With the current administration's UHC program, PhilHealth has reported 79% population coverage, with the poorest 9.6 million families now already being subsidized by the national and local government (71). In 2013, the NHIA was amended (72) to pave the way for massive reforms in benefit design and provider-payment mechanisms (such as shift from fee-for-service to case-based payments), as well as in reducing co-payments (such as through a ‘No Balance Billing’ policy for indigents) (73).

As social health insurance, PhilHealth is financed primarily through premiums (for both employed and self-employed) and tax-sourced government subsidies (for indigents, retirees, and pensioners). Part of the premium-based scheme is a separate program for overseas workers, which is now called the Overseas Filipinos Program (OFP) in order to also cover non-working Filipinos abroad, including irregular migrants, immigrants, dual citizens, and international students. Land-based OFWs are required to pay their premiums individually, while for sea-based OFWs (i.e. seafarers), shipping companies share the cost. As of January 2014, annual premium costs PhP 2,400 (USD 55). In 2013, there are 3.14 million paying members under the OFP, which also covers 2.73 million additional dependents, totaling 5.86 million or 7.6% of the total population covered (71). A unique feature of the PhilHealth governing structure is the presence of an OFW representative in its board of directors.

PhilHealth membership is mandatory for OFWs who got hired through the Philippine Overseas Employment Administration (POEA), the agency responsible for facilitating overseas deployment. PhilHealth enrolment is in addition to other requirements that are stipulated in the Migrant Workers and Overseas Filipinos Act of 1995, which also requires overseas employers to purchase the same private health insurance, along with other worker protection measures, being provided for their locally-hired employees. Nonetheless, those who were not able to enroll in PhilHealth prior to departure may register via the website or its collecting partners in selected countries. PhilHealth membership also covers dependents (spouse, children, elderly parents) accompanying the overseas Filipino in the destination country or being left behind in the Philippines. Conversely, despite the Philippines being a predominantly sending country, PhilHealth also allows foreign nationals residing or working in the Philippines to enroll in PhilHealth as individually-paying or employed members, provided that they present an Alien Certificate of Registration.

Utilization of healthcare overseas is also covered by PhilHealth; however, members pay out-of-pocket first to be later reimbursed (in contrast to utilization in the Philippines, in which PhilHealth directly pays the accredited healthcare provider). This system occasionally results in difficulties in reimbursements among migrants who are hospitalized overseas. Plans to enable online filing of claims and to contract primary care physicians abroad to care for covered OFWs remain in the pipeline (74). Furthermore, benefit coverage for hospitalizations overseas remains inadequate, as PhilHealth is using the case rates applied in hospitals based in the Philippines. Such scheme disregards the huge differences in medical care costs between the Philippines and overseas.

Besides PhilHealth, as earlier mentioned, the Philippines has a migrant welfare fund called OWWA. Although not required but highly encouraged, membership in OWWA costs USD 25. OWWA provides a wide range of services, from accident, burial, and disability benefits to medical, repatriation, and livelihood assistance. OWWA was also handling health insurance for OFWs until the function was transferred to PhilHealth in 2005. A major critique of OWWA is that membership expires at the same time as the end of employment contract, and therefore migrant workers cannot anymore receive benefits upon return to the Philippines (75).

#### Indonesia

Indonesia seems to be following in the Philippines’ footsteps both in terms of UHC and migrant protection. In January 2014, Indonesia announced its goal to achieve UHC by 2019 (76). The national health insurance program, called *Jaminan Kesehatan Nasional* (JKN), seeks to unify three main existing yet fragmented schemes: *Jamkesmas*, the government-financed health insurance program for the poor and near poor; *Askes* for civil servants and pensioners; and *Jamsostek* for formal sector workers. Prior to JKN, these three separate schemes only cover 40% of its 240 million population (77). In addition to providing health coverage, *Askes* and *Jamsostek* are also social security schemes that include employment injury, retirement, and death benefits (78).

Similar to PhilHealth, membership in the revitalized JKN is mandatory to all Indonesian citizens, as the three existing schemes failed to enroll the country's significantly huge informal sector. The program is to be funded mostly through premiums paid directly by self-employed and informal sector members, or deducted from wages for those employed either in public or private sector. On the contrary, Indonesia's poor – estimated at 86.4 million – are to be subsidized by the national government. JKN members are entitled to a range of personal health services, including promotive, preventive, curative and rehabilitative services (78).

As early as now, *Badan Penyelenggara Jaminan Sosial* (BPJS), a dedicated agency mandated to implement JKN, is already drawing critique from different corners for various reasons, such as inadequate and uncertain funding, lack of proper planning for health facilities and health workers, and poor information dissemination among the public, to name a few (79–82).

Since JKN is still evolving, it will take time before migrants are deliberately considered, like in PhilHealth's OFP. At present, health benefits are incorporated in the compulsory Migrant Worker Insurance Program, which includes illness, accident, and death coverage (83). Furthermore, like in the Philippines, bilateral agreements with select destination countries such as Malaysia stipulate overseas employers’ obligation to provide private health insurance for workplace accidents and pre-employment medical examinations (84). Despite the existence of such protective policies, implementation gaps remain, such as huge numbers of claims unprocessed by insurance companies and ill-defined coverage and excluded conditions (83, 84).

Finally, as a receiving country, Indonesia allows migrants who have worked for at least 6 months to enroll in JKN. However, foreigners in Indonesia are reluctant to join in the young scheme, identifying unclear conditions and redundant coverage as they are already provided with private health insurance by their employers (85).

## Discussion

### Redefining UHC for migrants

Table 3 summarizes the five ASEAN countries’ UHC developments as well as their migrant-related features. Overall, the five countries are not starting from scratch in terms of considering migrants in their respective health systems; however, all countries remain marred with implementation issues, from migrants still not covered with insurance in Thailand to difficulties in benefit reimbursements in the Philippines. Nonetheless, these countries can certainly do better in terms of enhancing migrant inclusion in UHC, primarily in terms of population coverage, but also in the two other dimensions of the WHO UHC cube framework – benefit coverage and level of financial protection – which are also touched in the succeeding discussion.

**Table 3:** Migrant-inclusive features of UHC in five ASEAN countries

	Receiving countries			Sending countries	
	
Parameter	Thailand	Malaysia	Singapore	Philippines	Indonesia
UHC overall design	Predominantly financed from general taxation for the poor and informal sector (UCS) and civil servants (CSMBS) combined with payroll taxes for those employed (SSS); membership mandated by law	Two-tiered system; public sector covering all Malaysian citizens funded by general taxes, while private sector funded through private health insurance and out-of-pocket spending	An innovative financing system comprised of government subsidies, mandatory premiums paid jointly by employer and employee, voluntary opt-out insurance for catastrophic illness, and government subsidy for the indigent; membership mandated by law	Social health insurance (PhilHealth) financed through premiums paid voluntarily (informal sector), payroll taxes (employed), or subsidy from national government budget from taxes (indigents); membership mandated by law	Social health insurance (JKN) financed through premiums paid voluntarily (informal sector), from payroll taxes (employed), or through subsidy from national government budget from taxes (indigents); membership mandated by law
Ongoing UHC developments/current status and challenges	Already achieved UHC especially for Thai citizens (in terms of population and benefit coverage as well as low out-of-pocket payments)	Already achieved UHC especially for Malaysian citizens; however, shift to social health insurance currently being considered	Already achieved UHC especially for Singaporean citizens (in terms of population and benefit coverage); still high out-of-pocket payments (58%)	79% population coverage; still high out-of-pocket payments (52%); fee-for-service payments shifted to case rates; outpatient packages still need to be rolled out; deadline for UHC set in 2016	UHC just recently rolled out in 2014; deadline for UHC set in 2019
Migrant-inclusive features of UHC	Separate scheme for legal migrant workers (CHMI) which also allows undocumented migrants to opt in; provides access to a comprehensive range of services, including antiretroviral treatment	Enrollment in private medical insurance schemes mandatory for legal migrants to avail of publicly-provided services; Workmen's Compensation Act provides guarantee for employer assistance for death and disability	Low- and semi-skilled migrants required to be enrolled in private health insurance by employers; Work Injury Compensation Act provides guarantee for employer assistance for disability and death	Separate procedure for membership for Overseas Filipinos but integrated with the national pool; covers overseas hospitalization and family members in country of destination or left behind; separate life insurance specific for migrant workers also exists (Overseas Welfare Workers Fund)	Migrant health insurance not yet part of UHC system but incorporated in compulsory Migrant Worker Insurance Program
Current status and challenges facing migrant inclusion in UHC	Annual premiums need to be paid by migrants themselves; benefits less comprehensive than those for Thai citizens	Migrants still need to be included in the government-run UHC system (beyond access to emergency care); higher co-payments charged against migrants; undocumented migrants totally left out	Migrants still need to be included in the 3M framework; insufficient benefits provided by private insurance; implementation problems due to unscrupulous employers and insurers	Difficult expansion to enroll undocumented migrants; benefits still inadequate due to overseas adoption of domestic case rates; delays and difficulties in processing reimbursements	Undocumented migrants remain uncovered with compulsory insurance; claims unprocessed by insurers; ill-defined packages and excluded conditions

Among the receiving countries, Thailand, a middle-income country that has already realized UHC for its citizens, can be rated as having gone the furthest in terms of ensuring migrant inclusion in UHC. Its parallel migrant scheme and the flexibility allowing undocumented migrants to opt into the system indicates that Thailand's progressive view of UHC goes beyond coverage on the basis of citizenship. This broad conceptualization of UHC is still yet to surface in the ongoing global discourse on UHC. There has been much talk about UHC being one of countries’ national responsibilities for the fulfillment of the right to health (86), but such a citizenship-based notion disregards a huge number of non-nationals living or working in a globalized and highly mobile world.

Singapore and Malaysia are two major destination countries that also claimed or were documented to have already achieved UHC. However, the UHC systems existing in these countries clearly pertain to universal coverage for their respective citizens only. There remains a considerable number of migrant workers and undocumented migrants in these two countries who are not covered by health insurance or inadequately covered with limited benefit packages and high co-payments. This is primarily due to their adoption of the private health insurance model for migrants, which is worsened by absence of strong regulation especially towards employers of migrant workers. If migrant coverage will be a criterion in gauging whether UHC has been achieved or not, then it can be concluded that Singapore and Malaysia have, in actuality, not yet realized UHC in the broadest sense.

In all three receiving countries, migrant workers are extremely dependent on employers for registration with authorities, insurance schemes, and health providers, as well as for their general upkeep and maintenance. Without proper monitoring and enforcement, employers can and do try to reduce costs by under-insuring workers or, for irregular migrants, not insuring them at all. The Ministries of Health of Malaysia and Singapore may therefore consider including migrants in their government-run UHC systems for their citizens, or developing a separate yet still government-run scheme such as what Thailand has done or implementing tight regulation should they prefer retaining their mandatory private health insurance models for migrants. Furthermore, in order to provide adequate health and financial protection, benefits should also be raised to a level that is on par with that provided for native workers and that is realistic given the average cost of healthcare overseas.

However, Thailand's CHMI scheme could be improved by ensuring portability within the country and by allowing premiums to be paid by installment, alleviating the financial burden imposed by lump sum payments. The Thai Ministry of Public Health may also ensure that hospitals relax documentary requirements in order to encourage undocumented migrants to purchase the CHMI.

Conversely, even before their respective UHC projects have commenced, the two sending countries, Indonesia and Philippines, have already begun considering health protection for the migrants that they deploy overseas. As earlier described, health insurance is previously embedded in the Philippines’ mandatory migrant welfare fund (OWWA); now it has already been transferred to PhilHealth. However, health protection still remains a part of the compulsory insurance for outgoing Indonesian migrants, and much work needs to be done to ensure its full implementation and hopefully eventual integration with the newly-established UHC system.

Today, the two countries are embarking on massive health financing reforms toward UHC, and migrant health protection is expected to become a key feature of their UHC systems in the near future. While Indonesia's very young UHC system will still have to focus its resources toward covering its non-moving citizens for now, the Philippines, however, provides a template for predominantly sending middle-income countries on ensuring inclusion of outbound migrants in universal coverage. While still facing operational challenges as well as the need for expanding insurance benefits and the proportion of costs covered, PhilHealth already allows overseas portability of insurance, offers benefit packages for conditions that are relevant to migrants such as viral pandemics (i.e. SARS, Influenza A(H1N1), MERS-CoV), and even extends the benefits to migrant families who are accompanying the migrant abroad or are left behind in the Philippines. Such measures demonstrate the need to reimagine UHC as systems that transcend national borders.

### UHC and migrant health as part of ASEAN's social protection agenda

The issue of UHC among migrants is also very much intertwined with the broader discourse on social protection, whose goal is to secure protection for citizens from lack of work-related income, lack of access to healthcare, insufficient family support, and general poverty and social exclusion (87). Since 2012, the International Labor Organization (ILO) has been advocating for the setting of national ‘social protection floors’ which guarantee access to essential healthcare and basic income security for children, unemployed adults, and older persons (88). Social protection for migrants is even emphasized in the United Nations General Assembly Resolution 40/144 on the human rights of individuals who are not nationals of the country in which they live (89).

The ASEAN regional bloc has also expressed commitment to social protection. In addition to provision of accessible healthcare services, the ASCC Blueprint also identified social welfare and protection as a priority, and envisioned putting in place social safety nets to protect citizens from the negative impacts of integration and globalization (19). While the measures laid down in the blueprint, such as mapping of social protection regimes in ASEAN and the establishment of a social insurance system to cover the informal sector, remain a work-in-progress, there is room for building coherence among the related agendas of social protection, migrant welfare, and UHC.

### UHC – including undocumented migrants?

While challenges in providing health coverage for legal migrant workers by both source and destination countries are now being gradually tackled, coverage among undocumented or irregular migrants, including seasonal migrants, one-day or circular migrants (those who move in for a week or months and then back and come again), and stop-over migrants (those who stay for a while before moving to another country), has oftentimes been avoided due to its sensitive political nature. For instance, the ASEAN Declaration emphasized that ‘the receiving states and sending states shall, for humanitarian reasons, closely cooperate to resolve the cases of migrant workers who, through no fault of their own, have subsequently become undocumented’. However, the Declaration also underscored that it does not imply regularization of the situation of migrant workers who are undocumented (18). This poses a challenge as undocumented persons and refugees, who are not included in existing UHC systems, comprise some of the most vulnerable and marginalized migrant subgroups facing higher health risks and therefore requiring greater attention.

ASEAN member countries may also emulate examples of similar regional blocs that have extensive experience in improving access to healthcare among migrants, including undocumented migrants who are not covered with private health insurance, for instance by their employers. For example, while most countries in Europe provide no more than emergency services for undocumented migrants, some countries either provide more services or allow undocumented migrants to opt into national insurance schemes upon meeting certain requirements such as payment of premiums (90). Among the five ASEAN countries, Thailand and Philippines present some progress though, as both countries already allow irregular migrants (inbound and outbound, respectively) to enroll into the migrant arm of their respective UHC systems.

Including irregular migrants in UHC, whether through tax-based, premium-based or other potential forms of financing, will require a deliberate decision to separate the issue of irregular migrant status from people's entitlement to accessing essential healthcare. While this may not be problematic from a public health perspective, such a stance may strike some sensitive chords in other sectors such as those governing migration and labor policies. Covering undocumented migrants may be misconstrued as condoning irregular migration, even if international human rights instruments that guarantee the right to health to all people regardless of migrant status already exist. Given this situation, crucial policy decisions made by agencies from outside the health sector, such as those that deal with overseas labor, immigration issues and diplomatic relations, are critical. Ministries of Health of ASEAN countries therefore must actively negotiate with their counterparts in government to advocate for realizing the health rights of irregular migrants through UHC. Ultimately, the issue of healthcare access regardless of migrant status may require broadening the focus of migration discourse in ASEAN from mere “ASEAN migrant workers” to “ASEAN citizens.”

### Harnessing ASEAN's open dialogue approach to advance migrant health

For almost half a century, ASEAN has nurtured among its member countries a culture of continuous and open dialogue. In fact, the regional bloc is originally conceived as a loose network of countries to function in that manner, until the idea of a more integrated ASEAN community was conceived in 2003. Nevertheless, regional integration, especially economic integration, demands a deeper level of dialogue about shared pressing issues such as migration and health. These issues, however, cannot be resolved overnight – for example, seven years have already passed since the signing of the ASEAN Declaration on the Protection and Promotion of Rights of Migrant Workers and the instrument that will serve as its implementing guideline is still yet to be finalized and approved.

Nonetheless, promoting migrant health has already been recently identified as a priority in the ASEAN Strategic Framework on Health Development (91). Three of the study countries – Indonesia, Philippines, and Thailand – serve as the lead countries in this area of cooperation. In 2012, Indonesia hosted a workshop on migrant health to develop a set of recommendations for increasing access to health services for migrants. Another possible platform where migrant integration in UHC systems can be discussed is the recently-created ASEAN Plus Three (China, Japan, South Korea) UHC Network (92).

Outside of formal ASEAN platforms, member countries may also take the bilateral route and discuss with counterpart countries on how to improve financial coverage and access to healthcare among migrants. In terms of the ASEAN integration, UHC and migrant health nexus, the roles of origin and destination countries are equally important, particularly in the face of health system inequities between neighboring countries. For instance, Thailand has a more developed health system compared to those in Cambodia, Myanmar and Laos, making it an attractive place to access healthcare among migrants. For political and internal security reasons (e.g. disease control), Thailand and the surrounding countries already have established bilateral collaborations between their respective public health systems (93).

Recently, Thailand and Cambodia have signed an MOU to develop border health services to be implemented by designated national task forces. At a July 2013 meeting, the countries agreed to improve the referral system and care for critically ill patients, as well as non-critical case referrals across the border. One proposal involved building ‘sister hospital’ networks of Thai and Cambodian hospitals on both sides of the border to facilitate cross-border referrals (93). There are several other examples of bilateral cooperation of technical expertise sharing, human resource development and infectious disease control between Thailand, Cambodia, Laos and Myanmar, indicating that an inclusive migrant health approach involves close cooperation with neighboring countries. For example, a series of dialogues has already been undertaken to explore how these countries can jointly address health policy, financing, and care delivery issues for migrants crossing the Thai border (94). Challenging as they may seem due to the huge diversity of healthcare financing arrangements among countries, co-financing mechanisms between sending and receiving countries may also be explored.

### Migrant health and UHC – a new research agenda

One of the major challenges faced during the conduct of this review is the dearth of literature examining migrant health in general, and migrant health in connection to UHC or health systems in particular. To date, limited academic and policy research on how migrants access health services in ASEAN countries means that we do not have a full understanding of the health challenges they face throughout the entire migration cycle. Clearly, there is a need to develop a research agenda that examines this nexus of migration and health (95) and to ensure that health systems and UHC are part of it. Furthermore, research at the country level is therefore highly encouraged, and these studies can feed into the broader regional discourse on migrant inclusion in UHC.

Comparisons between countries also pose a challenge due to the diversity of UHC designs, migration profiles, and migrant protection schemes, not to mention the reliability of data on migration. A monitoring and evaluation framework can later be developed to allow a more comprehensive and robust cross-country comparison. Finally, the link between migration and UHC requires transdisciplinary research, as the question of how UHC can be made migrant-inclusive will need inputs not just from the public health and health systems perspective, but also from labor studies, political science, and international affairs, to name a few.

## Conclusions

In the coming years, with the ongoing move toward regional integration, ASEAN will continue to be a highly dynamic and mobile region. Hence, ASEAN countries should capitalize on the momentum built by both ASEAN integration and the UHC agenda in order to build migrant-inclusive health systems. Origin and destination country efforts to improve migrant health coverage are equally important, and there are more ways than one to ensure that migrants are included in UHC.

The reasons for including migrants in UHC in ASEAN countries are many. First and foremost, addressing the health needs of migrants in ASEAN is a matter of human rights and social justice, which are fundamental principles already enshrined in the regional bloc's numerous instruments. Moreover, it is in ASEAN's best interest to protect the health of migrants as it pursues the regional path toward collective social progress and economic prosperity. Indeed, healthy migrants contribute to the advancement of human capital in both sending and receiving countries, thereby creating healthy communities and healthy economies. ASEAN can also take leadership in the ongoing global conversation on the shape of the post-2015 development agenda, particularly the health goal which is most likely to incorporate UHC. Finally, the region can demonstrate to the rest of the world that UHC can and should go beyond health protection on the basis of citizenship, and therefore must ensure the inclusion of non-nationals (96), and that UHC can be reimagined as systems that transcend national borders. Leaving out migrants in the UHC agenda is clearly not ‘universal’ at all, and is therefore a huge step backward from achieving its very goal – access to affordable and quality healthcare for all, anywhere, all the time.

## References

[1] Association of Southeast Asian Nations (ASEAN) (2007). ASEAN economic community blueprint.

[2] The University of Queensland Mutual recognition arrangements (MRA) concepts. CBAMT – competency standards for tourism in ASEAN states.

[3] Kaur A (2007). On the move: international migration in Southeast Asia since the 1980s. Hist Compass.

[4] Asis MM (2004). Borders, globalization and irregular migration in Southeast Asia. International migration in Southeast Asia.

[5] Asian Forum of Parliamentarians on Population and Development, AusAID, Global Alliance Against Traffic in Women Policy brief: human trafficking in South-East Asia. http://www.afppd.org/files/1113/8206/9530/Policy_Brief.pdf.

[6] Cameron S (2007). Trafficking and related labour exploitation in the ASEAN region. http://www.icsw.org/doc/Trafficking%20Labour%20Exploitation%20in%20ASEAN%2007.pdf.

[7] United Nations Office on Drugs and Crime (UNODC) (2012). Global report on trafficking in persons, 2012.

[8] ASEAN (2006). ASEAN framework agreement on visa exemption. http://www.asean.org/communities/asean-political-security-community/item/asean-framework-agreement-on-visa-exemption-kuala-lumpur-25-july-2006-2.

[9] Asians favour single ASEAN visa Travel Daily Media. http://www.traveldailymedia.com/199423/asians-favour-single-asean-visa/.

[10] International Organization for Migration (IOM) Mainstreaming health into the migration and development dialogue. http://www.iom.int/files/live/sites/iom/files/What-We-Do/docs/PositionPaper_HMD_1106.pdf.

[11] Gushulak BD, MacPherson DW (2004). Globalization of infectious diseases: the impact of migration. Clin Infect Dis.

[12] United Nations The universal declaration of human rights. http://www.un.org/en/documents/udhr/.

[13] World Health Organization (WHO) (1946). Constitution of the World Health Organization.

[14] United Nations (1966). International covenant on economic, social and cultural rights.

[15] United Nations General Assembly (1990). International convention on the protection of the rights of all migrant workers and members of their families.

[16] WHO (2008). World Health Assembly resolution 61.17 on health of migrants.

[17] IOM (2013). World migration report 2013: migrant well-being and development.

[18] ASEAN (2007). Declaration on the protection and promotion of the rights of migrant workers. http://www.asean.org/communities/asean-political-security-community/item/asean-declaration-on-the-protection-and-promotion-of-the-rights-of-migrant-workers-3.

[19] ASEAN (2009). ASEAN socio-cultural community blueprint.

[20] ASEAN and Joint United Nations Initiative on Mobility and HIV/AIDS in South East Asia (JUNIMA) (2011). Multi-Stakeholder Dialogue on Migrants' Health and Access to HIV Services in the ASEAN Region - Meeting Report. http://www.junima.org/news_documents/MSDNov11/MSD_ASEAN_Meeting_Report_Migrant_Workers_Health_Access.pdf.

[21] IOM (2008). Migration and HIV/AIDS in Thailand: a desk review of migrant labour sectors. http://www.migrationcenter.mahidol.ac.th/download/Migration%20and%20HIV%20AIDS%20Desk%20Review%20final.pdf.

[22] Jitthai N, Yongpanichkul S, Bijaisoradat M (2010). Migration and HIV/AIDS in Thailand: triangulation of biological, behavioural and programmatic response data in selected provinces. http://www.migrationcenter.mahidol.ac.th/download/PDF_HIV%20Triangulation%20Report_Final.pdf.

[23] National Epidemiology Center, Department of Health (2014). Philippine HIV and AIDS registry. http://www.pnac.org.ph/uploads/documents/publications/NEC_HIV_April-AIDSreg2014.pdf.

[24] (2014). HIV-positive OFWs seen topping 3000-mark. http://www.interaksyon.com/article/87572/hiv-positive-ofws-seen-topping-3000-mark.

[25] United Nations Development Programme (UNDP) (2010). Regional dialogue on the health challenges for Asian labour migrants - Meeting Report. http://www.globalmigrationgroup.org/sites/default/files/uploads/gmg-topics/health/Regional_Dialogue_on_the_Health_Challenges_for_Asian.pdf.

[26] WHO Questions and answers on universal health coverage. http://www.who.int/contracting/documents/QandAUHC.pdf?ua=1.

[27] Huang Y (2012). World momentum builds for universal health coverage. The new global health agenda: universal health coverage.

[28] Tangcharoensathien V, Patcharanarumol W, Ir P, Aljunid SM, Mukti AG, Akkhavong K (2011). Health-financing reforms in southeast Asia: challenges in achieving universal coverage. Lancet.

[29] WHO (2014). World Health Assembly resolution 67.14 on health in the post-2015 development agenda.

[30] Lagomarsino G, Garabrant A, Adyas A, Muga R, Otoo N (2012). Moving towards universal health coverage: health insurance reforms in nine developing countries in Africa and Asia. Lancet.

[31] WHO (2010). World health report 2010 – health systems financing: the path to universal coverage.

[32] Muhidin S, Utomo A (2013). How many overseas Indonesians are there?. (Discussion Paper Series No.1/2013) Indonesia Diaspora Network.

[33] Martin P (2007). The economic contribution of migrant workers to Thailand: towards policy development. http://www.ilo.org/wcmsp5/groups/public/---asia/---ro-bangkok/documents/publication/wcms_098230.pdf.

[34] Ministry of Manpower, Singapore Government (2014). Levies & quotas for hiring foreign workers. http://www.mom.gov.sg/foreign-manpower/foreign-worker-levies/Pages/levies-quotas-for-hiring-foreign-workers.aspx.

[35] (2014). 2,156 undocumented migrant workers from Indonesia caught in Malaysia. http://www.thejakartapost.com/news/2014/02/24/2156-undocumented-migrant-workers-indonesia-caught-malaysia.html.

[36] Bangko Sentral ng Pilipinas (Central Bank of the Philippines) Overseas Filipinos’ Cash Remittances 2010–2014. http://www.bsp.gov.ph/statistics/spei_pub/Table%2023.pdf.

[37] Ambito JS, Banzon MSL (2011). Review of Philippine migration laws and regulations: gains, gaps, prospects. http://dirp4.pids.gov.ph/ris/dps/pidsdps1137.pdf.

[38] Agunias DR, Ruiz NG (2007). Protecting overseas workers: lessons and cautions from the Philippines. http://www.migrationpolicy.org/research/protecting-overseas-workers-lessons-and-cautions-philippines.

[39] Blank NR (2011). Making migration policy: reflections on the Philippines’ bilateral labor agreements. Asian Polit Policy.

[40] ILO (2013). 10 years of work on labour migration in Indonesia. http://www.ilo.org/wcmsp5/groups/public/---asia/---ro-bangkok/---ilo-jakarta/documents/publication/wcms_213361.pdf.

[41] Hugo G (2007). Indonesia's labor looks abroad. http://www.migrationpolicy.org/article/indonesias-labor-looks-abroad.

[42] United Nations, Department of Economic and Social Affairs, Population Division (2013). Trends in international migrant stock: the 2013 revision – migrants by destination and origin. http://esa.un.org/unmigration/TIMSO2013/documents/MIgrantStocks_Documentation.pdf.

[43] Commission on Filipinos Overseas (2012). Global mapping of overseas Filipinos. http://www.cfo.gov.ph/index.php?option=com_content&view=article&id=1339:global-mapping-of-overseas-filipinos-&catid=134:statisticsstock-estimate&Itemid=814.

[44] Philippine Overseas Employment Administration (2012). 2008–2012 overseas employment statistics. http://www.poea.gov.ph/stats/2012_stats.pdf.

[45] Huguet J, Chamratrithirong A, Natali C (2012). Thailand at a crossroads: challenges and opportunities in leveraging migration for development. http://www.migrationpolicy.org/research/Thailand-Leveraging-Migration.

[46] Haseltine W (2013). Affordable excellence: the Singapore healthcare story.

[47] Evans TG, Chowdhury MR, Evans DB, Fidler AH, Lindelow M, Mills A (2012). Thailand's universal coverage scheme: achievements and challenges. An independent assessment of the first 10 years (2001–2010).

[48] Yu CP, Whynes DK, Sach TH (2008). Equity in health care financing: The case of Malaysia. Int J Equity Health.

[49] Chee HL (2008). Ownership, control, and contention: challenges for the future of healthcare in Malaysia. Soc Sci Med.

[50] National Health Security Office, Thailand (2002). National Health Security Act B.E. 2545 (A.D. 2002). http://www.nhso.go.th/eng/Files/Userfiles/file/Thailand_NHS_Act.pdf.

[51] Srithamrongsawat S, Wisessang R, Ratjaroenkhajorn S (2009). Financing healthcare for migrants: a case study from Thailand. http://publications.iom.int/bookstore/free/Financing_Healthcare_for_Migrants_Thailand.pdf.

[52] Press B (2014). New health insurance for migrant workers facing challenges. http://www.phamit.org/hilight-detail.php?lang=en&id=12.

[53] Health Insurance Group (2013). Health card for uninsured foreigners and health card for mother and child. Seminar on measures and protocols of medical examination, insuring migrants and protecting maternal and child health.

[54] ASTV Manager Online (2006). The Ministry of Public Health failed to have migrants registered to the insurance, only 66 thousand health cards purchased [Internet]. http://www.manager.co.th/qol/viewnews.aspx?NewsID=9560000156809.

[55] Sunpuwan M, Chamchan C Management of health insurance system and health service provision for migrant workers in Thailand: case studies from Samut Prakan and Chiang Mai.

[56] Economic Transformation Programme (ETP) (2014). Healthcare – EPP 1: mandating private insurance for foreign workers. http://etp.pemandu.gov.my/Healthcare-@-Healthcare_-_EPP_1-;_Mandating_Private_Insurance_for_Foreign_Workers.aspx.

[57] AXA Hospitalisation & surgical scheme for foreign workers. http://www.axa.com.my/861/en/health-insurance/group-health/spikpa.

[58] Chua H, Cheah J (2012). Financing universal coverage in Malaysia: a case study. BMC Public Health.

[59] Sen TE (2012). Labour dept: foreign workers must be insured. http://www.freemalaysiatoday.com/category/nation/2012/05/12/labour-dept-foreign-workers-must-be-insured/.

[60] Hector C, Somwong P (2014). Employers must pay all medical cost of workers especially when it is a work-related accident or an occupational disease. http://www.shanland.org/index.php?option=com_content&view=article&id=3421:employers-must-pay-all-medical-cost-of-workers-especially-when-it-is-a-work-related-accident-or-an-occupational-disease&catid=102:mailbox&Itemid=279.

[61] Liew H (2013). SOCSO – what is it? Employer/employee responsibilities and more. http://my.news.yahoo.com/guide-socso-malaysia-personal-accident-insurance-scheme-222833874.html.

[62] Jaafar S, Noh KM, Muttalib KA, Othman NH, Healy J (2013). Malaysia health system review (Health Systems in Transition, Vol. 3 No. 1 2012). http://www.wpro.who.int/asia_pacific_observatory/hits/series/Malaysia_Health_Systems_Review2013.pdf.

[63] Ahmad ZA (2013). Third-class ward charge in govt hospitals reduced by half from May 1. http://www.thestar.com.my/News/Nation/2012/04/19/Thirdclass-ward-charge-in-govt-hospitals-reduced-by-half-from-May-1/.

[64] Alhadjri A, Cheng D (2013). Repeal archaic law to protect migrant workers: Tenaganita. http://www.thesundaily.my/news/747601.

[65] Ho CWL (2013). The equity of universal health coverage: some reflections from Singapore. http://www.brookings.edu/research/opinions/2013/12/09-singapore-universal-health-coverage-ho.

[66] Lim J (2013). Myth or magic: the Singapore healthcare system.

[67] Ministry of Manpower, Singapore Government (2014). Work injury compensation act: frequently asked questions. http://www.mom.gov.sg/legislation/occupational-safety-health/Pages/work-injury-compensation-act-faqs.aspx.

[68] Johnraj M, Bacolod N (2013). The buck stops where? A healthcare policy trilemma for Singapore's Foreign Workers. http://lkyspp.nus.edu.sg/wp-content/uploads/2013/07/LKYSPP-Case-Study-A-Healthcare-Policy-Trilemma-for-Singapores-Foreign-Workersl.pdf.

[69] (2014). Injured workers inside and outside WICA: processes, problems and solutions. http://twc2.org.sg/2014/02/14/injured-workers-inside-and-outside-wica/.

[70] Republic of the Philippines (1994). Republic Act No. 7875. http://www.philhealth.gov.ph/about_us/ra7875.pdf.

[71] PhilHealth. Stats & charts (2013). http://www.philhealth.gov.ph/about_us/statsncharts/snc2013.pdf.

[72] Republic of the Philippines (2012). Republic Act No. 10606. http://www.philstar.com/science-and-technology/768702/philhealth-implements-no-balance-billing-scheme.

[73] Crisostomo S (2012). PhilHealth implements “no balance billing” scheme. http://www.philstar.com/science-and-technology/768702/philhealth-implements-no-balance-billing-scheme.

[74] (2012). OFWs may soon file claims online for hospital bills incurred abroad – PhilHealth. http://globalnation.inquirer.net/47718/ofws-may-soon-file-claims-online-for-hospital-bills-incurred-abroad-philhealth.

[75] (2013). Group to pursue lifetime OFW membership to OWWA, pensions for retired OFWs. http://www.remate.ph/2013/01/group-to-pursue-lifetime-ofw-membership-to-owwa-pensions-for-retired-ofws/.

[76] (2014). Indonesia strides towards universal health care. Lancet.

[77] Thabrany H (2014). Birth of Indonesia's “medicare”: fasten your seatbelts. http://www.thejakartapost.com/news/2014/01/02/birth-indonesia-s-medicare-fasten-your-seatbelts.html.

[78] ILO (2008). Indonesia: implementation of the national social security system law (Series on social security extension initiatives in South East Asia). http://www.ilo.org/wcmsp5/groups/public/---ed_protect/---soc_sec/documents/publication/wcms_secsoc_6595.pdf.

[79] Slater A Criticism builds of Indonesia's government-sponsored health insurance scheme. http://www.globalsurance.com/news/2014/03/31/criticism-builds-of-indonesia%E2%80%99s-government-sponsored-health-insurance-scheme/.

[80] (2014). Hopes and fears as Indonesia rolls out universal healthcare. http://www.irinnews.org/report/99463/hopes-and-fears-as-indonesia-rolls-out-universal-healthcare.

[81] Johnson Co (2014). Indonesia: universal health care program implemented. http://www.loc.gov/lawweb/servlet/lloc_news?disp3_l205403816_text.

[82] Hewson J (2014). Indonesia's innovative healthcare scheme. http://www.aljazeera.com/indepth/features/2014/01/indonesia-innovative-healthcare-scheme-201411485331598453.html.

[83] Farbenblum B (2013). Migrant workers’ access to justice at home: Indonesia.

[84] Hall A (2011). Migrant workers’ rights to social protection in ASEAN: case studies of Indonesia, Philippines, Singapore and Thailand. http://www.fes.org.ph/media/Publications_PDF_Files/14._Migrant_Workersrsquo_Rights___to_Social_Protection__in_ASEAN.pdf.

[85] Osman N (2014). Foreign workers reluctant to join national health insurance. http://www.thejakartapost.com/news/2014/01/04/foreign-workers-reluctant-join-national-health-insurance.html.

[86] Gostin LO, Ooms G, Heywood M, Haffeld J, Møgedal S, Røttingen J-A (2010). The joint action and learning initiative on national and global responsibilities for health. http://www.who.int/healthsystems/topics/financing/healthreport/JALI_No53.pdf.

[87] ILO (2010). World social security report 2010: providing coverage in times of crisis and beyond.

[88] ILO and WHO (2011). Social protection floor for a fair and inclusive globalization - report of the advisory group. http://www.who.int/healthsystems/topics/financing/healthreport/JALI_No53.pdf.

[89] United Nations General Assembly (1985). Resolution 40/144. Declaration on the human rights of individuals who are not nationals of the country in which they live.

[90] Gray BH, van Ginneken E (2012). Health care for undocumented migrants: European approaches. Issue Brief (Commonw Fund).

[91] Fernando FM (2013). ASEAN strategic framework on health development (2010–2015): increase access to health services for ASEAN people. http://www.searo.who.int/thailand/news/asean_health_cooperation_increasing_access_to_health_people_dr_ferdinal.pdf.

[92] (2014). http://ihppthaigov.net/images/DRAFT_Minute_SC_Meeting_ASEANPlusThreeUHC_Network.pdf.

[93] Kantayaporn T, Malik S (2013). Migration and health service system in Thailand: situation, responses, and challenges in a context of AEC in 2015.

[94] International Health Policy Programme, Thailand (2014). Conference Series on Migrant Health in the Mekong Region: The Second Consultative Meeting on Policy, Health Financing and Health Service Delivery Issues. http://ihppthaigov.net/index.php/2011-08-15-07-10-58/events/item/190-conference-series-on-migrant-health-in-the-mekong-region-the-second-consultative-meeting-on-policy-health-financing-and-health-service-delivery-issues.

[95] Benach J, Muntaner C, Chung H, Benavides FG (2010). Immigration, employment relations, and health: developing a research agenda. Am J Ind Med.

[96] Brolan CE, Dagron S, Forman L, Hammonds R, Latif LA, Waris A (2013). Health rights in the post-2015 development agenda: including non-nationals. Bull World Health Organ.

# Sub article: The ASEAN economic community and medical qualification

**DOI:** 10.3402/gha.v7.24535

**Published:** 2014-09-10

**Authors:** Jathurong Kittrakulrat, Witthawin Jongjatuporn, Ravipol Jurjai, Nicha Jarupanich, Krit Pongpirul

**Affiliations:** 1 Medical Students for Health Systems and Services (MS-HSS), Thailand Research Center for Health Services System (TRC-HS), Faculty of Medicine, Chulalongkorn University, Bangkok, Thailand; 2 Department of Preventive and Social Medicine, Faculty of Medicine, Chulalongkorn University, Bangkok, Thailand; 3 Department of International Health, Johns Hopkins Bloomberg School of Public Health, Baltimore, MD, USA; 4 Bumrungrad International Hospital, Bangkok, Thailand

**Keywords:** AEC, medical licensing examination, medical qualification, medical education, medical practice

## Abstract

**Background:**

In the regional movement toward ASEAN Economic Community (AEC), medical professions including physicians can be qualified to practice medicine in another country. Ensuring comparable, excellent medical qualification systems is crucial but the availability and analysis of relevant information has been lacking.

**Objective:**

This study had the following aims: 1) to comparatively analyze information on Medical Licensing Examinations (MLE) across ASEAN countries and 2) to assess stakeholders’ view on potential consequences of AEC on the medical profession from a Thai perspective.

**Design:**

To search for relevant information on MLE, we started with each country's national body as the primary data source. In case of lack of available data, secondary data sources including official websites of medical universities, colleagues in international and national medical student organizations, and some other appropriate Internet sources were used. Feasibility and concerns about validity and reliability of these sources were discussed among investigators. Experts in the region invited through HealthSpace.Asia conducted the final data validation. For the second objective, in-depth interviews were conducted with 13 Thai stakeholders, purposely selected based on a maximum variation sampling technique to represent the points of view of the medical licensing authority, the medical profession, ethicists and economists.

**Results:**

MLE systems exist in all ASEAN countries except Brunei, but vary greatly. Although the majority has a national MLE system, Singapore, Indonesia, and Vietnam accept results of MLE conducted at universities. Thailand adopted the USA's 3-step approach that aims to check pre-clinical knowledge, clinical knowledge, and clinical skills. Most countries, however, require only one step. A multiple choice question (MCQ) is the most commonly used method of assessment; a modified essay question (MEQ) is the next most common. Although both tests assess candidate's knowledge, the Objective Structured Clinical Examination (OSCE) is used to verify clinical skills of the examinee. The validity of the medical license and that it reflects a consistent and high standard of medical knowledge is a sensitive issue because of potentially unfair movement of physicians and an embedded sense of domination, at least from a Thai perspective.

**Conclusions:**

MLE systems differ across ASEAN countries in some important aspects that might be of concern from a fairness viewpoint and therefore should be addressed in the movement toward AEC.

The Association of Southeast Asian Nations (ASEAN) is the geo-political and economic cooperation across ten countries: Brunei Darussalam, Cambodia, Indonesia, Laos, Malaysia, Myanmar, the Philippines, Singapore, Thailand, and Vietnam. To promote free trade and services across boundaries, an initiative called the ASEAN Economic Community (AEC) will start in 2015 (1). According to the Mutual Recognition Arrangement (MRA) of this regional movement, physicians, nurses, and dentists are among seven selected professional groups (physician, nurse, dentist, accountant, engineer, architect, and surveyor) that can be qualified to practice in another country (2). The flow of health professionals and cross-border health services is seen as crucial to the success of AEC but needs to be evaluated (3).

Literature on the potential implications of international trade in health services has focused only on the exchange of health care providers and patients across borders or mal-distribution of health resources across urban and rural areas (4, 5). An analysis of current trade patterns based on a ‘four modes of supply’ framework has focused on location and movement of suppliers and consumers; (4) however, this framework may be too simplified for health care professionals, especially physicians. The four modes – cross-border supply, consumption abroad, commercial presence, and movement of individual service providers – did not touch upon the production of suppliers, which is also included in the current context. No framework that integrates both pre- and post-production as well as migration of health care professionals in ASEAN has been proposed in the literature.

Lessons from the European Union (EU) about physician migration could inform the AEC initiative for health care professionals. Health professionals’ migration is affected by push and pull factors (6, 7) as people are more likely to move from their current job with low pay, poor working conditions, as well as limited career opportunities to a relatively better position in another site. Since countries may have different standards, qualifications, and linguistic requirements, there is a period of adapting to clinical, organizational, and social culture of the new country. Unlike non-health care professions, clinical practice relies not only on medical knowledge and skill, but also on interpersonal communication with patients and relatives (8). EU experience suggested that language is one of the most important factors that affect the movement of professional groups, especially in the health care sector (6).

To be a physician, one must complete the required professional medical training, be conferred the professional medical qualification, and be licensed by the Professional Medical Regulatory Authority (PMRA) in the Country of Origin documenting they are technically, ethically, and legally qualified to undertake professional medical practice (2). This privilege usually is not automatically recognized by the ‘Host Country’ (2) – a country where a foreign medical practitioner applies for registration to practice medicine. Australia, for example, requires overseas-trained health professionals to pass fitness-to-practice assessments prior to being registered to practice (9).

Medical education and physician migration are related (10). On the one hand, unequal educational capacity leads to imbalances in the physician workforce (11). On the other hand, a critical mass of physicians is needed to sustain and enhance the medical education enterprise. Further, some countries intentionally train a surplus of health care professionals to supply other countries. Until now, evidence relevant to both AEC and health services were mainly about graduated health professionals but not about medical education and qualification systems.

Current attempts to prepare for the transition to the AEC have focused more on medical education than qualifications (10). The President of the Medical Council of Thailand announced an effort to open more medical schools not only to support the Thai government's medical hub policy but also resolve the doctor shortage problem (12).

Comparable medical qualification systems are crucial to ensure a ‘fair exchange’ of physician workforces among countries. Differences will need to be addressed as part of the effort to harmonize the systems and to realize the MRA and free flow of medical practitioners. Despite the existence of PMRAs in each country (Table 1), good analysis and synthesis of relevant information on medical qualifications in the ASEAN region, has been lacking. The objectives of the present study were (1) to comparatively analyze information on medical licensing examination (MLE) systems across ASEAN countries and (2) to assess stakeholders' view on potential consequences of the AEC on the medical profession from a Thai perspective.

**Table 1:** Professional Medical Regulatory Authority (PMRA) of ASEAN Countries

Member State	Professional Medical Regulatory Authority (PMRA)
Brunei Darussalam	Brunei Medical Board
Cambodia	Cambodian Medical Council and Ministry of Health
Indonesia	Indonesian Medical Council and Ministry of Health
Lao PDR	Ministry of Health
Malaysia	Malaysian Medical Council
Myanmar	Myanmar Medical Council, Ministry of Health
Philippines	Professional Regulation Commission, Board of Medicine and Philippine Medical Association
Singapore	Singapore Medical Council and Specialists Accreditation Board
Thailand	Thailand Medical Council and Ministry of Public Health
Vietnam	Ministry of Health

Source: 2009 ASEAN Mutual Recognition Arrangement on Medical Practitioners. 14th ASEAN Summit, February 26, 2009; Cha-am, Thailand.

## Methods

This study is comprised of two components. To search for relevant information on the MLEs, we included data from the ten national authorities potentially responsible for MLEs of each country as our primary data source. We initially evaluated official websites and made additional queries using email or telephone where possible. Data from these sources were considered most reliable and valid, but were not always available for some countries such as Singapore that does not have centralized MLE, or Brunei that only imports physicians.

For countries lacking a primary data source or with incomplete data, we needed to use data from alternative sources. To gain a better understanding of the national systems, we first checked the official website of medical schools listed in the International Medical Education Directory (IMED) that offered MLE-relevant information (13).

We then approached our colleagues in the Asian Medical Students’ Association (AMSA) – the largest medical student community in Asia (14). This insider's information was quite reliable but might be incomplete. Our third source of data was the Chula-ASEAN Medical Schools Initiative (CU-AMSI) – a collaboration between Chulalongkorn University, Thailand; University of Health Sciences of Cambodia; University of Health Sciences, Laos PDR; University of Medicine 1, Yangon, Myanmar; University of Medicine 2, Yangon, Myanmar; University of Pharmacy, Yangon Myanmar to strengthen the countries’ capacity in medical education and research. Medical students from these countries were informally interviewed to assess their knowledge about MLEs. Some essential information missed by the above approaches was retrieved from other Internet sources as appropriate. Feasibility and concerns about validity and reliability of the data from these secondary sources were discussed among the investigators. The initial synthesis of information was sent to experts in the region, who were invited through HealthSpace.Asia connections. They were asked to validate the findings specific to their countries and then to provide some corrections with supporting evidence.

**Table 2:** Comparing Medical Licensing Examination across 10 ASEAN Countries

		Thailand	Philippines	Singapore	Indonesia	Malaysia	Vietnam	Myanmar	Cambodia	Lao PDR	Brunei
National authority		Center for Medical Competency Assessment and Accreditation	Philippines Board of Medicine	Singapore Medical Council	Indonesia Medical Council	Malaysia Medical Council	Health Ministry/Provincial Agency	Myanmar Medical Council	National Exit Exam Committee and Medical Council of Cambodia	National Medical Council of Laos	Brunei Medical Board
Language in		English 50%	English	English	Bahasa	English		English	Khmer	English,	No
examination		Thai 50%			Indonesia			Burmese		Laos	
Official language		Thai	Philipino English	English, Malaysian, Chinese	Bahasa Indonesia	Malaysian, English	Vietnamese	Burmese	Khmer	Laos	Bahasa Melayu
Steps		3	1	5	1	1		1	1,3	1	0
Methods	MCQ	Yes			Yes	Yes					No
of examination	MEQ	Yes	Yes								No
	OSCE	Yes			Yes	Yes					No
# Medical schools		21	43	2	73	24	12	8	2	1	1
Duration of courses	Pre-clinic	3	3	2	3	2	3	3	6	3	No
(years)	Clinic	3	1	3	3	3	3	3–3.5	2	3	No
	Total	6	5	5	6	5	6	6–7	8	6	No
Doctor:Patient		1:2,700	1:1,800	1:580	1:7,700	1:1,400	1:1,900	1:2,800	1:6,300	1:1,700	1:736
Population (×1,000)		67,312	94,013	5,077	234,181	28,909	86,930	52,797	15,296	6,230	415
Centralization		Yes	No	No	No		No				

For stakeholder analysis, in-depth interviews were conducted with 13 Thai experts. Based on maximum variation sampling technique (15), they were purposively selected to represent the medical licensing authority, the medical profession, ethicists as well as economists. The interview guide contained questions about the impact of AEC on the medical profession, the validity of the medical license, and other important issues relevant to MLE. The interviews were voice recorded and transcribed in Thai language. After all data had been collected, the investigators initially familiarized themselves with the data by listening to tapes and re-reading transcribed interviews in order to identify key ideas and recurrent themes until the investigators became familiar with them in their entirety. A coding scheme was then developed by drawing on a priori issues and questions derived from the study objectives, points raised during the interviews, as well as themes that recurred in the data. The coding scheme was used to code all transcripts. Atlas.ti 6 software (Scientific Software Development GmbH, Berlin, Germany) was used to facilitate the qualitative data analysis.

This study was part of a project submitted to a Medical Ethics course, Faculty of Medicine, Chulalongkorn University. The two components of this study had minimal ethical concerns and were not submitted for consideration by the Institutional Review Board (IRB). The first part of this study deals with only publicly available information. Although the second part of this project involved subjects who were key informants, data was ‘from’ them but not ‘about’ them. Rather, it was opinions and judgments about MLE system from a Thai perspective. Therefore, the project did not qualify as human subjects research as defined by United States Department of Health and Human Services regulations 45 CFR 46.102, and did not require IRB review, which also concurs with relevant Thai regulations.

## Results

Brunei has the best doctor to patient ratio despite no medical school or MLE (Table 2). In most countries, MLEs are run by their respective medical council, which usually is a part of the country's government health ministry. With regard to length of time of recognition of qualification, some countries, including Thailand, offer life-long certification, whereas Vietnam requires a renewal every 5 years.

Thailand and Indonesia have a national MLE system whereas Singapore and Vietnam accept results of MLE conducted at universities. Although the MLE of most countries requires only one step, Thailand adopts the USA's 3-step approach that aims to check pre-clinical knowledge, clinical knowledge, and clinical skills. In Cambodia, the MLE system is comprised of two components. The National Exit Exam Committee is responsible for organizing the national examination and the Medical Council of Cambodia will provide a license to practice to medical doctors who pass the examination.

Regarding examination types, multiple choice question (MCQ) formats are the most commonly used, followed by the modified essay question (MEQ) format. Although both tests try to assess a candidate's knowledge, the Objective Structured Clinical Examination (OSCE) was used to verify clinical skills of the examinee.

Medical education systems vary across ASEAN countries and affect the development of MLE systems. Medical students usually were required to complete 5–7 years of coursework. In Singapore, medical students must pass an annual examination in their medical schools before they can proceed to the next level. Immediately following successful completion of the fifth-year examination, they receive provisional registration which is valid only for the internship period; successful completion of the internship allows progression to full registration. This 5-step MLE system is similar to Thailand's system before centralization.

Language variation across countries does exist not only for general communication, but also in the MLE. English is used in seven countries whereas six prefer the local language, especially in the OSCE. Although Thailand's official language is Thai, approximately 10% of the written examinations for pre-clinical and clinical knowledge are in English. Similar phenomena occur in Myanmar, Cambodia, and Laos where English examination questions are added to the ones in their respective native languages. Interestingly, Malaysia uses only English rather than its native language. Vietnam and Indonesia are the only countries that do not use the English language in its MLE at any stage.

Three major themes emerged from in-depth interviews with 13 Thai experts. The first theme is the extent to which a medical license issued by one country is valid in other countries. Two interesting concepts emerged from the interviews. ‘License transfer’ happens when a license issued by country A is valid in both country A and B. This, from the interviewee's perspective, seems to result in a sense of domination by country A. ‘Common license’ means a license jointly issued by and therefore valid in all participating countries. Although this common license forms a sense of community, only 2 out of 13 interviewees thought that it is possible to achieve; the rest disagreed mainly because of diverse systems and contexts across ASEAN countries in the quality of medical education, language used, and culture. ‘Each country has its own rule and context, to which whoever would like to do clinical practice must comply. As the country systems have been developed to promote the national interest, not regional, I consider an ASEAN common license unethical’, stated one interviewee. All interviewees still thought that further development of medical education and practice in this region is essential.

The second theme is about the language used in each step of the licensing examination. Ten out of thirteen interviewees said basic science and clinical knowledge examination should be executed in a common language like English. Experts from all but the economic field stressed the importance of using native language in assessing clinical competency not only must knowledge and skill be evaluated but also interpersonal communication. ‘It would be unfair to a Thai patient if his or her doctor does not speak the same language. Drug prescription and surgical operation are important, but the patient would be worse off without a clear understanding of what the doctor tried to explain’.

The third theme is that migration of both supply and demand of health care services is complex. Although many interviewees voiced a concern about the migration of Thai physicians to places with better financial benefits, seven thought that non-financial aspects of the Thai context may be more influential including returning to Thailand for family or social reasons. On the contrary, the movement of both medical practitioners and patients from other countries into Thailand is more likely but the impact on health systems is inconclusive.

## Discussion

This study is the first to offer comparative information on medical qualification systems across ASEAN countries. In addition to different basic characteristics of medical education, MLE systems in ASEAN countries are diverse in many aspects including language, number of steps in the process, as well as methods of examination. These differences need to be addressed as part of the harmonization effort. Findings from an online survey suggested that ‘recognition of qualification’ should be standardized and could start from basic functions such as licensure/registration, especially when evidence on the competence of regulators and their diversity were still unclear (16).

The initial assumption that information on medical education and MLE systems would be readily accessible from a country's national body turned out to be invalid as our attempts to identify a primary source revealed incomplete information. Secondary sources became important for our data analysis and synthesis despite questionable quality when standard criteria were applied (17). The objective of this study was just to compare the systems in general.

Unlike individuals in other professions, health care professionals, especially doctors, require not only technical but also interpersonal skills. Evidence suggested that quality of care can be adversely affected by a language barrier (18, 19). In addition to language, evidence consistently suggested that race and ethnicity also substantially influenced the quality of the doctor-patient relationship (20). Validity of the medical license is a sensitive issue, at least from the Thai perspective. The potential imbalance or unfair movement of physicians and relevant policies or agreements across ASEAN countries embed a sense of domination (21); this inevitably distorts the concept of unity. Any political negotiation regarding the medical license validity should be carefully done, however.

Currently, there are some attempts to unify the medical curriculum and the examination system across India (22) and the Emirates (23) to diminish regional differences; however, the application of these experiences to ASEAN region without adequate data is limited. Information presented in this study is beneficial for potential harmonization of medical education and qualification systems across national boundaries. We hope that the comparative data and the Thai perspective presented in this study will provide input for the collaborative development of a framework for smooth implementation of relevant systems to facilitate free movement of doctors across ASEAN countries under the Mutual Recognition Arrangements. As only Thai perspective was focused in the present study, further harmonized efforts should be done by representatives of all ASEAN countries to synthesize country specific concerns.

## Conclusion

MLE systems differ across ASEAN countries in some important aspects that might be of concern from a fairness viewpoint and therefore should be addressed in the movement toward AEC.

## References

[1] Kanithasen P, Jivakanont V, Boonnuch C (2011). AEC 2015: ambitions, expectations and challenges ASEAN's path towards greater economic and financial integration.

[2] (2009). 2009 Asean Mutual Recognition Arrangement on Medical Practitioners.

[3] Lim J, Umareddy I, Cockerham WC, Dingwall R, Quah SR (2014). Health care services, cross-border. The Wiley Blackwell encyclopedia of health, illness, behavior, and society.

[4] Arunanondchai J, Fink C (2006). Trade in health services in the ASEAN region. Health Promot Int.

[5] Supakankunti S, Herberholz C, Tullao TS, Lim HH (2012). Transforming the ASEAN Economic Community (AEC) into A Global Services Hub: enhancing the competitiveness of the health services sector in Thailand. Developing ASEAN Economic Community (AEC) into A Global Services Hub, ERIA Research Project Report 2011–1.

[6] Gerlinger T, Schmucker R (2007). Transnational migration of health professionals in the European Union. Cadernos de saude publica.

[7] World Health Organization (2006). Health worker migration in the European region: country case studies and policy implication.

[8] Dyche L (2007). Interpersonal skill in medicine: the essential partner of verbal communication. Can J Gen Intern Med.

[9] Vaughan B, Sullivan V, Gosling C, McLaughlin P, Fryer G, Wolff M (2012). Assessing fitness-to-practice of overseas-trained health practitioners by Australian registration & accreditation bodies. BMC Med.

[10] Norcini JJ, Mazmanian PE (2005). Physician migration, education, and health care. J Contin Educ Health Prof.

[11] Marchal B, Kegels G (2003). Health workforce imbalances in times of globalization: brain drain or professional mobility?. Int J Health Plann Manag.

[12] Khaopa W (2013). More varsities offering medical courses. The Nation.

[13] Foundation for Advancement of International Medical Education and Research (FAIMER) The International Medical Education Directory (IMED). http://www.faimer.org/resources/imed.html.

[14] AMSA AMSA International. http://amsa-international.org/about/.

[15] Patton M (1990). Qualitative evaluation and research methods.

[16] Bescanon LJR, Rockey P, Zanten Mv (2012). Regulation of health professions: disparate worldwide approaches are a challenge to harmonization. World Med J.

[17] Sorensen HT, Sabroe S, Olsen J (1996). A framework for evaluation of secondary data sources for epidemiological research. Int J Epidemiol.

[18] Timmins CL (2002). The impact of language barriers on the health care of Latinos in the United States: a review of the literature and guidelines for practice. J Midwifery Wom Health.

[19] Bernard A, Whitaker M, Ray M, Rockich A, Barton-Baxter M, Barnes SL (2006). Impact of language barrier on acute care medical professionals is dependent upon role. J Prof Nurs.

[20] Ferguson WJ, Candib LM (2002). Culture, language, and the doctor-patient relationship. Family Med.

[21] Stewart A (2001). Theories of power and domination: the politics of empowerment in late modernity.

[22] (2011). Medical Council of India hints at unified medical education system from 2013. DNA. http://www.dnaindia.com/india/report-medical-council-of-india-hints-at-unified-medical-education-system-from-2013-1528341.

[23] Saberi M Professionals welcome unified medical licensing in UAE 2014. http://gulfnews.com/news/gulf/uae/health/professionals-welcome-unified-medical-licensing-in-uae-1.1284477.

# Sub article: Horizontal inequity in public health care service utilization for non-communicable diseases in urban Vietnam

**DOI:** 10.3402/gha.v7.24919

**Published:** 2014-08-04

**Authors:** Vu Duy Kien, Hoang Van Minh, Kim Bao Giang, Lars Weinehall, Nawi Ng

**Affiliations:** 1 Center for Health System Research, Hanoi Medical University, Hanoi, Vietnam; 2 Department of Public Health and Clinical Medicine, Unit of Epidemiology and Global Health, Umeå University, Umeå, Sweden; 3 Institute for Preventive Medicine and Public Health, Hanoi Medical University, Hanoi, Vietnam

**Keywords:** healthcare utilization, horizontal equity, non-communicable diseases, decomposition, urban Vietnam

## Abstract

**Background:**

A health system that provides equitable health care is a principal goal in many countries. Measuring horizontal inequity (HI) in health care utilization is important to develop appropriate and equitable public policies, especially policies related to non-communicable diseases (NCDs).

**Design:**

A cross-sectional survey of 1,211 randomly selected households in slum and non-slum areas was carried out in four urban districts of Hanoi city in 2013. This study utilized data from 3,736 individuals aged 15 years and older. Respondents were asked about health care use during the previous 12 months; information included sex, age, and self-reported NCDs. We assessed the extent of inequity in utilization of public health care services. Concentration indexes for health care utilization and health care needs were constructed via probit regression of individual utilization of public health care services, controlling for age, sex, and NCDs. In addition, concentration indexes were decomposed to identify factors contributing to inequalities in health care utilization.

**Results:**

The proportion of healthcare utilization in the slum and non-slum areas was 21.4 and 26.9%, respectively. HI in health care utilization in favor of the rich was observed in the slum areas, whereas horizontal equity was achieved among the non-slum areas. In the slum areas, we identified some key factors that affect the utilization of public health care services.

**Conclusion:**

Our results suggest that to achieve horizontal equity in utilization of public health care services, policy should target preventive interventions for NCDs, focusing more on the poor in slum areas.

A health system that provides equitable health care is a principal goal in many countries in the world. Horizontal equity in healthcare utilization is defined as ‘equal treatment for equal medical need, irrespective of other characteristics such as income, race, place of residence, etc.’ (1–3). Although several achievements in health care have been observed, the distribution of health care utilization is still inequitable (4, 5). Studies show that people’s health needs are not addressed equitably in many developed and developing countries (5–7). The evidence also suggests that after controlling for needs, health care distribution that benefits the rich greatly exceeds distribution that benefits the poor (6, 8, 9). Appropriate methods of measuring horizontal inequity (HI) in health care utilization play a key role in contributing to the development of appropriate public policies for health care systems (5, 7).

Since Vietnam’s economic reform in 1986, the country has achieved significant results in economic development. In addition, the health of the country’s population has improved substantially. Average life expectancy at birth increased from 67.8 years in 2000 to 73 years in 2012 (10, 11). The infant mortality rate decreased from 44.4 per 1,000 live births in 1990 to 15.4 per 1,000 live births in 2012 (11). However, Vietnam is suffering a double burden of diseases wherein communicable diseases still exist while non-communicable diseases (NCDs) are increasing (12). Along with the rapid economic growth, Vietnam has undergone a dramatic period of urbanization. The urban population increased from 24% in 1999 to 30% in 2009 (13).

The health care system in Vietnam is a mixed public–private provider system with the public sector dominating. The quality of health service delivery remains inadequate and poor for the population (12, 14, 15). Universal health coverage (UHC) aims to ensure that all people have access to health care services at affordable cost. In 2008, Vietnam approved a law on Social Health Insurance (SHI) to create a national SHI program, and in 2013 it developed a project to implement a roadmap toward UHC in 2012–2015 and a vision of UHC in 2020 (11). The Vietnam Living Standard Surveys have reported a gap between the rich and the poor in terms of health care utilization in different cross-sectional periods (16–19). Another study also reports that health care utilization in Vietnam changes in several years (15). There is however little information about horizontal inequality in health care utilization in urban areas, especially in slum areas. Thus, the aim of this study is to identify HI in health care utilization in urban slum area in Vietnam. In addition, we identify relevant health factors, including self-reported NCDs, associated with inequity in health care utilization.

## Methods

### Study setting

The study was carried out in Hanoi, the capital city of Vietnam, which has 29 districts of which 10 are urban and 19 are rural. Hanoi’s population is estimated to be 6.5 million, of which 2.6 million (41%) live in urban districts (20). In this study, we purposely selected four urban districts, namely Ba Dinh, Dong Da, Hai Ba Trung, and Hoan Kiem, as they included both slum and non-slum areas. We adapted a definition of the United Nations that slum areas are ‘those groups of households whose people live in temporary houses, insecure locations, and narrow spaces, or near a polluted environment’ (21).

### Study design and data collection

We conducted a population-based cross-sectional study in February–March 2013. Face-to-face interviews using structured questionnaires were conducted with the heads of households. The interviews were carried out by trained medical students of Hanoi Medical University and were supervised by senior staff of the Center for Health System Research, Hanoi Medical University.

### Sample size and sampling

The sample size was determined using a level of significance of 0.05 with a relative precision of 0.4, while an anticipated 10% of households had at least one member with a NCD (we estimate this prevalence in a pilot study of 60 households) (22). We control for a 30% non-response rate and design effects of 2 because of the use of cluster sampling. Thus, the targeted sample size is at least 600 households in either the non-slum or the slum areas. A mapping study was conducted to determine the list of 84 slum areas in the four urban districts. We randomly selected 30 slum areas from a list of 84 slum areas and 30 non-slum areas in the same commune by matching each area. In each area, we targeted a selection of approximately 20 households. A household is defined as comprising one person or a group of people who share accommodation and meals for at least 6 months during the past 12 months. The first household was randomly selected at the center of the slum or non-slum area, and the next household was the one adjacent to the previous. In each area, the data collections were concluded when the total number of interviewed households reached 20. In each selected households, all individuals 15 year old or over were invited to the study.

### Variables

To measure healthcare utilization, we asked about visits to public health facilities in the past 12 months prior to the interviews. Some people reported using private health care services; however, in this study, we focus only on those who reported accessing public health care services, which dominate the health care provision in Vietnam. In addition, public health care facilities participate fully in the national SHI scheme. Public health facilities include commune health centers, district hospitals, provincial hospitals, and national hospitals. In this study, we focus on the four main diseases that account for 80% of the total mortality from NCDs: cardiovascular diseases (heart disease or stroke), diabetes, chronic respiratory diseases (chronic obstructive pulmonary disease and asthma), and cancer (malignant tumors) (23).

### Measurement of wealth index

Since data for household income are not reliable, especially in developing countries, the wealth index, which is a composite index composed of key asset ownership variables were used as a proxy indicator for socioeconomic status (24, 25). We collected information on living conditions, including construction materials used in dwellings (e.g. materials for roofs, walls, and floors), access to utilities and infrastructure (e.g. sanitation facilities and sources of water), and ownership of selected durable assets (e.g. TVs, radios, computers, Internet access, telephones, mobile phones, VCD/DVD players, refrigerators, washing machines, water heaters, motorbikes, and cars). We applied principal components analysis (PCA) to construct a wealth index for our study population, separately for the slum and non-slum areas (26). The variables with multiple categories, such as material used in dwelling construction, were broken down into a set of dummy variables. Simple descriptive statistical analysis was performed to check the relevant variables; we used the rule of thumb that any variable with a prevalence below 5% or higher than 95% should be excluded from the analysis.

### Indirect health care need standardization

An indirect method was used to compare the differences between actual need and need standardized distributions for the probability of public health care utilization during a year. Non-linear estimation was used to account for the large proportion of observations with no utilization of public health care services (27). A probit model with control variables was used to estimate the difference between need predicted use and actual use. In addition, we used both ordinary least squares (OLS) and probit models to estimate need standardized health care use with and without controls.

The method proposed by O’Donnell et al. was applied to standardize indirect health care need (3, 27). The linear model of the relationship between, on one hand, public health care utilization and, on the other hand, need and control variables can be estimated by the following equation.

1 $$y_{i}=\alpha +\sum_{j}\beta_{j}x_{ji}+\sum_{k}\gamma_{k}z_{ki}+\varepsilon_{i}$$

where *y*_*i*_ is a public health care utilization variable; *i* denotes the individual; and *α*, *β*, and *γ* are parameter vectors ɛ is the error. *x*_*j*_ are confounding variables that we want to standardize, and *z*_*k*_ are non-confounding variables that we do not want to standardize but instead control in order to estimate partial correlations with the confounding variables. We used the OLS model to estimate the predicted value for public health care utilization by the following equation.

2 $${\hat{y}}_{i}^{x}=\hat{\alpha }+\sum_{j}{\hat{\beta }}_{j}x_{ji}+\sum_{k}{\hat{\gamma }}_{k}z_{ki}$$

where ${\hat{y}}_{i}^{x}$ is the predicted value of public health care utilization, and $\hat{\alpha }$, ${\hat{\beta }}_{j}$, and ${\hat{\gamma }}_{k}$ are OLS parameters. Finally, estimates of indirectly standardized public health care utilization ${\hat{y}}_{i}^{IS}$ are given by the difference between actual public health care utilization (*y*_*i*_) and predicted public health care utilization ${\hat{y}}_{i}^{x}$, plus the overall sample mean $\bar{y}$:

3 $${\hat{y}}_{i}^{IS}=y_{i}-{\hat{y}}_{i}^{x}+\bar{y}$$

The non-linear model of the relationship between public health care utilization, *y*, which is binary, and need (*x*) and control (*z*) variables in terms of a general functional form, *G*, is presented as follows:

4 $$y_{i}=G\left(\alpha +\sum_{j}\beta_{j}x_{ji}+\sum_{k}\gamma_{k}z_{ji}\right)+\varepsilon_{i}$$

where *G* takes particular forms for the probit model. Then the standardized need was estimated using the following equation (3).

5 $${\hat{y}}_{i}^{IS}=y_{i}-G\left(\hat{\alpha }+\sum_{j}{\hat{\beta }}_{j}x_{ji}+\sum_{k}{\hat{\gamma }}_{k}{\bar{z}}_{k}\right)+\frac{1}{n}\times \sum_{i=1}^{n}G\left(\hat{\alpha }+\sum_{j}{\hat{\beta }}_{j}x_{ji}+\sum_{k}{\hat{\gamma }}_{k}{\bar{z}}_{k}\right)$$

where *n* is the sample size and *z* variables are set to their means ${\bar{z}}_{k}$ to obtain the predictions. As noted above, ${\hat{y}}_{i}^{IS}$ is the standardized public health care utilization.

### Marginal effects

Since the outcome variable, self-reported NCDs, was binary with the range of (0,1), we applied the probit model to extract marginal effects of each determinant on observed probabilities of the outcome variable. The marginal effects provide evidence of associations between determinants and the outcome variable (self-reported NCDs). The marginal effects with positive signs indicate that they have positive association with the probability of self-reported NCDs, while those with negative signs indicate negative associations. A large absolute value of a marginal effect presents a strong association.

### Health inequality analysis

According to egalitarian principles, health care should be distributed based on a need principle rather than people’s willingness and ability to pay. It is suggested that if health care is allocated appropriately, health equity will be promoted. However, need is a very difficult concept to define and measure (1, 3). In this study, we used demographic characteristic variables (age–sex dummy variables) and NCDs as a proxy measure for need (Table 1). In order to measure inequity, inequality in public health care utilization was standardized for differences in need. After standardizing this inequality, any residual inequality in utilization by socioeconomic status (based on the wealth index) is interpreted as HI, which would be pro-rich if its value is positive and pro-poor if its value is negative.

**Table 1:** Variables included in this study

Categories	Variables
Healthcare utilization	The probability of any public healthcare utilization
Healthcare need	Sex–Age, self-reported of non-communicable diseases
Control variables	Wealth index, education, occupation, health insurance

To evaluate HI in our model, we applied the same method used to estimate the concentration index (CI) of actual health care use and health care need (1, 3). The value of HI is measured as the difference between the CI of health care use and that of health care need. The CI is directly related to the concentration curve, which plots the cumulative percentage of the health care utilization variable (on the y-axis) and the cumulative percentage of socioeconomic variables ranked from poorest to richest (on the x-axis). The CI is calculated as twice the area between the concentration curve and the line of equality (the 45° line). The CI ranges between −1 and +1. The CI takes a value of 0 if health care utilization distribution is completely equal. It is negative when the concentration curve lies above the line of equality, which indicates greater concentration of the health care utilization variable among the poor. Meanwhile, it takes a positive value if the concentration curve lies below the line of equality, which indicates greater concentration of the health care utilization variable among the rich. The CI of health care use (C_M_) was calculated by the following equation.

6 $$C=\frac{2}{\mu }Cov(h,r)$$

where *µ* is the mean of the health outcome, *h* is health care utilization of the individual, and *r* is rank of the individual by wealth distribution (1). Similarly, if we replace *h* with the health care need of the individual, we can calculate the CI of health care needs (C_N_). The HI was calculated by subtracting *C*_*N*_ from *C*_*M*_. A positive value of HI indicates a distribution of health care utilization in favor of the rich, and vice versa.

7 $$HI_{WV}=C_{M}-C_{N}$$

where HI_WV_ is a horizontal index using the indirect standardization approach. The CI of public health care utilization can be decomposed into the contribution of determinants based on the linear function in Equation 1. The CI for *y* can be written as

8 $$C=\sum_{j}(\beta_{j}{\bar{x}}_{j}/\mu )C_{j}+\sum_{k}(\gamma_{k}{\bar{z}}_{k}/\mu )C_{k}+GC_{S}/\mu$$

If a non-linear model is used, then decomposition is possible only if some linear approximation to the non-linear model is made. One possibility is to use estimates of the partial effects evaluated at the means. That is, a linear approximation to Equation 4 is given by

9 $$y_{i}=\alpha^{m}+\sum_{j}\beta_{j}^{m}x_{ji}+\sum_{k}\gamma_{k}^{m}z_{ki}+\varepsilon_{i}$$

where $\beta_{j}^{m}$ and $\gamma_{k}^{m}$ are the partial effects of each variable treated as a fixed parameter and evaluated at the sample means, and *ɛ*_*i*_ is the error, which includes approximation errors. Then, the CI for *y* for this non-linear approach can be written as

10 $$C=\sum_{j}(\beta_{j}^{m}{\bar{x}}_{j}/\mu )C_{j}+\sum_{k}(\gamma_{k}^{m}{\bar{z}}_{k}/\mu )C_{k}+GC_{S}/\mu$$

where *µ* is the mean of y; ${\bar{x}}_{j}$ is the mean of *x*_*j*_; and ${\bar{z}}_{k}$ is the mean of *z*_*k*_, GC_ɛ_ is the generalized concentration index for the error term (ɛ) (3).

Data were analyzed using Stata statistical software version 12.1. The level of statistical significance was set to 0.05. Missing data were excluded from the data analysis.

### Ethical considerations

The protocol for this study has been approved by the Scientific and Ethical Committee in Biomedical Research, Hanoi Medical University. All human subjects in the study were asked for their consent, and they had provided their consent before data collection, and all had complete rights to withdraw from the study at any time without threats or disadvantages.

## Results

A total of 1,211 households were included in the study (600 and 611 households in the non-slum and slum areas, respectively). The non-response rate, about 3–5%, is quite similar in both slum and non-slum areas. Our inclusion criteria for participants are those who are 15 years old or older, and this generates a total of 3,815 people in our sample.

### Poor–rich distribution of public health care utilization and their determinants

Table 2 presents the proportion and CI for public health care utilization. The self-reported public health care utilization variables show that people in both slum and non-slum areas used mainly national hospitals. In general, 21.3% of people in slum areas reported visiting any public health care facility during the past 12 months while 26.7% of those in non-slum areas reported the same. The crude concentration indexes for all levels and any public health care utilization for the non-slum areas were negative, indicating that health care utilization was concentrated among the poor in the non-slum areas. In the slum areas, the crude concentration indexes for visiting district (C=−0.063) and provincial hospitals (C=−0.112) were negative, indicating that at district and provincial hospitals, health care utilization was concentrated among the poor. However, the crude concentration indexes for communal health centers (C=0.044) and national hospitals (C=0.114) were positive, indicating that at these levels, health care utilization was concentrated among the rich in the slum areas.

**Table 2:** Proportion and concentration indexes of public healthcare utilization during the past 12 months and the distributions of their determinants for slum and non-slum areas in Hanoi

	Slum areas		Non-slum areas	
	
Variables	Proportion (in decimal)	Concentration index	Proportion (in decimal)	Concentration index
Public healthcare utilization				
Communal health center	0.020	0.044	0.017	−0.157
District hospitals	0.035	−0.063	0.038	−0.020
Provincial hospitals	0.053	−0.112	0.073	−0.049
National hospitals	0.118	0.114	0.148	−0.101
Any public healthcare utilization	0.213	0.034	0.267	−0.074
Age–sex				
Males aged 15–29	0.137	0.016	0.090	0.121
Males aged 30–44	0.124	0.053	0.133	0.079
Males aged 45–59	0.109	−0.016	0.110	−0.014
Males aged 60+	0.094	0.069	0.137	−0.115
Females aged 15–29	0.135	0.055	0.125	0.068
Females aged 30–44	0.148	−0.041	0.135	0.061
Females aged 45–59	0.137	−0.083	0.118	−0.016
Females aged 60+	0.117	−0.029	0.152	−0.126
Wealth index				
Wealth quintile: 1 – lowest 20%	0.167	−0.833	0.161	−0.839
Wealth quintile: 2 – lower 20%	0.180	−0.486	0.214	−0.465
Wealth quintile: 3 – middle 20%	0.209	−0.098	0.197	−0.054
Wealth quintile: 4 – higher 20%	0.212	0.323	0.230	0.373
Wealth quintile: 5 – highest 20%	0.233	0.767	0.199	0.801
Education				
Education: primary school or less	0.187	−0.381	0.060	−0.270
Education: secondary school	0.281	−0.151	0.181	−0.119
Education: high school	0.282	0.099	0.271	0.008
Education: college/university or higher	0.250	0.345	0.487	0.073
Work status				
Work status: professionals, technicians, or social services	0.139	0.300	0.238	0.139
Work status: worker, farmer, or crafts worker	0.178	−0.029	0.124	−0.061
Work status: self-employed	0.289	−0.149	0.187	−0.039
Not in workforce: unemployed	0.053	−0.315	0.025	−0.253
Not in workforce: retired	0.204	0.073	0.310	−0.083
Not in workforce: other, such as not decided to work or student/pupil	0.138	0.060	0.116	0.120
Non-communicable diseases	0.079	−0.077	0.116	−0.169
Health insurance	0.658	0.109	0.826	0.013

The proportions for categories of determinants in Table 2 showed distributions of respondents across these categories. The crude concentration indexes showed the poor–rich distribution of the determinants. In the non-slum areas, people aged 60 years or older were strongly concentrated among the poor (C=−0.115 for males, C=−0.126 for females). In contrast, in the slum areas, people aged 60 years or older were concentrated among the poor for only females (C=−0.029), but among the rich for males (C=0.069). For those aged 45–59 years, both sexes were concentrated among the poor in both the non-slum and slum areas. The socioeconomic status inequality gradient can be seen clearly; people in lower wealth quintiles were concentrated more among the poor, while people in higher wealth quintiles were concentrated more among the rich. In addition, the socioeconomic gradient in education was clear. Those who graduated from secondary school or less were poorer, while those who graduated from high school or more were richer. Those who were manual workers, self-employed, or unemployed were concentrated among the poor. In the slum areas, about 8% of respondents who reported having NCDs were concentrated among the poor (C=−0.077). In non-slum areas, about 12% of respondents who reported having NCDs were concentrated among the poor (C=−0.169) in the non-slum areas. About 83% of people are covered by health insurance in the non-slum areas, but only 66% of people are covered by health insurance in the slum areas. Those with health insurance were concentrated among the rich in both non-slum (C=0.109) and slum areas (C=0.013).

### Marginal effects of determinants

Table 3 shows the marginal effects of each determinant on each public health care utilization variable. Increasing age in both men and women was significantly associated with increased probabilities of reporting public health care utilization, and the effects were consistently largest for respondents aged 60 years or older in both slum and non-slum areas. There was no association between wealth quintiles and the probability of reporting public health care utilization. However, NCDs and the presence of health insurance had strong positive associations with the probability of public health care utilization.

**Table 3:** Probability of determinants on any public healthcare utilization during the past 12 months in slum and non-slum areas in Hanoi (marginal effect using the probit model)

Variables	Slum areas	Non-slum areas
Age–sex		
Males aged 15–29	ref.	ref.
Males aged 30–44	−0.022	0.034
Males aged 45–59	0.101*	0.190**
Males aged 60+	0.272***	0.363***
Females aged 15–29	−0.066	−0.023
Females aged 30–44	0.032	0.065
Females aged 45–59	0.234***	0.362***
Females aged 60+	0.212***	0.464***
Wealth index		
Wealth quintile: 1 – lowest 20%	ref.	ref.
Wealth quintile: 2 – lower 20%	0.039	0.168
Wealth quintile: 3 – middle 20%	−0.008	−0.003
Wealth quintile: 4 – higher 20%	0.048	0.016
Wealth quintile: 5 – highest 20%	0.031	−0.003
Education		
Education: primary school or less	ref.	ref.
Education: secondary school	−0.005	−0.082*
Education: high school	0.009	0.019
Education: college/university or higher	−0.021	−0.008
Work status		
Work status: professionals, technicians, or social services	ref.	ref.
Work status: worker, farmer, or craft worker	0.030	0.028
Work status: self-employed	0.050	0.033
Not in workforce: unemployed	0.090	0.066
Not in workforce: retired	0.093*	0.052
Not in workforce: other, such as not decided to work or student/pupil	−0.008	−0.035
Non-communicable diseases		
No	ref.	ref.
Yes	0.268***	0.229***
Health insurance coverage		
No	ref.	ref.
Yes	0.113***	0.115***

Dependent variable for the probit model is a dichotomous indicator of whether a person has had any health care during the past 12 months or not, and indicates significance level as follows:

*** p≤0.001,

** 0.001<p≤0.01,

* 0.01<p≤0.05.

### Horizontal equity

In the slum areas, as Table 4 shows, the results of our estimation clearly suggested that the actual distribution observed was pro-rich (a higher mean among the wealthiest) and the need expected distribution was pro-poor in the slum areas (a higher mean among the poorest). This was because ‘need’, as proxied by demographics and NCDs, was concentrated more among the lower socioeconomic status groups in the slum areas. After using need standardization, the mean of the wealthiest 20% using public health care services was 0.217, which was 26% higher than the mean of the poorest 20% (0.172). On the contrary, for the non-slum areas, as Table 5 shows, we found that actual and need predicted public health care utilization were concentrated more among the poor. This means that in the non-slum areas, the poor were more likely to have the opportunity to use public health care services. Thus, we do not take our analysis further for the non-slum areas.

**Table 4:** Distributions of actual, need predicted, and need standardized healthcare utilization in slum areas in Hanoi

Probability of any public health care utilization							

				Need standardized			
		
	Probit with controls			With controls		Without controls	
	
Quintiles	Actual	Need predicted	Different=predicted−actual	Probit	OLS	Probit	OLS
Wealth quintile: 1 – lowest 20%	0.174	0.201	−0.027	0.172	0.172	0.176	0.176
Wealth quintile: 2 – lower 20%	0.223	0.213	0.011	0.210	0.210	0.207	0.207
Wealth quintile: 3 – middle 20%	0.203	0.196	0.009	0.207	0.206	0.205	0.205
Wealth quintile: 4 – higher 20%	0.255	0.196	0.060	0.259	0.259	0.259	0.259
Wealth quintile: 5 – highest 20%	0.208	0.190	0.018	0.217	0.217	0.217	0.217
Mean	0.213	0.199	0.014	0.213	0.213	0.213	0.213
Concentration index/*HI*_*WV*_	0.034	−0.019		0.052	0.050	0.050	0.050
Standard error	0.028	0.010		0.025	0.025	0.025	0.025
t-ratio	1.214	−1.889		2.033	1.998	1.995	2.011

OLS=Ordinary Least Square model; probit=probit model; HI_WV_=horizontal inequity index using the indirect standardization approach.

**Table 5:** Distributions of actual, need predicted, and need standardized healthcare utilization in non-slum areas in Hanoi

Probability of any public health care utilization							

				Need standardized			
				
	Probit with controls			With controls		Without controls	
	
Quintiles	Actual	Need predicted	Different=predicted−actual	Probit	OLS	Probit	OLS
Wealth quintile: 1 – lowest 20%	0.335	0.313	0.022	0.280	0.281	0.273	0.273
Wealth quintile: 2 – lower 20%	0.271	0.250	0.021	0.278	0.278	0.278	0.279
Wealth quintile: 3 – middle 20%	0.222	0.246	−0.024	0.234	0.234	0.236	0.236
Wealth quintile: 4 – higher 20%	0.262	0.239	0.022	0.280	0.280	0.281	0.282
Wealth quintile: 5 – highest 20%	0.243	0.237	0.006	0.263	0.262	0.265	0.265
Mean	0.267	0.257	0.010	0.267	0.267	0.267	0.267
Concentration index/*HI*_*WV*_	−0.075	−0.066		−0.011	−0.013	−0.004	−0.004
Standard error	0.022	0.010		0.019	0.019	0.019	0.019
t-ratio	−3.364	−6.456		−0.580	−0.667	−0.222	−0.234

OLS=Ordinary Least Squares; HI_WV_=horizontal inequity index using the indirect standardization approach.

For the slum areas, we extend our analysis to decompose the effects of need factors and non-need factors in the utilization of public health care services. Table 6 shows that the contribution of each need factor was negative, indicating that if utilization was determined by need factors alone, it would be pro-poor. After standardizing the percentage of contribution, the aggregate contribution of all need factors decreased the unstandardized CI by about 61%. When we examined the need factors in detail, the distribution of age–sex groups and NCDs pushed utilization in a pro-poor direction by about 40 and 21%, respectively. In addition, if needs were distributed equally, the direct effect of the wealth index and health insurance coverage on health care utilization would increase the unadjusted CI by approximately 37 and 54%, respectively, which pushed utilization in a pro-rich direction. At the same time, if needs were distributed equally, education and work status reduced the unadjusted CI by approximately 8 and 31%, respectively, thus pushed the unadjusted CI in a pro-poor direction.

**Table 6:** Decomposition of concentration index for public health care utilization in slum areas in Hanoi

Contributions to concentration index for any healthcare utilization						

	OLS			Probit partial effects		
	
	Absolute	Percentage	Standardized percentage	Absolute	Percentage	Standardized percentage
Need factors						
Age–sex groups	−0.010	−29.05	−34.02	−0.015	−43.47	−39.86
Non-communicable diseases	−0.008	−24.43	−28.61	−0.008	−22.78	−20.89
Subtotal	−0.018	−53.48	−62.63	−0.023	−66.25	−60.75
Non-need factors						
Wealth index	0.026	76.81	41.88	0.026	76.76	36.88
Health insurance coverage	0.036	106.59	58.12	0.038	113.17	54.38
Education	−0.003	−8.85	−10.36	−0.003	−8.55	−7.84
Work status	−0.007	−20.03	−23.46	−0.012	−34.25	−31.41
Subtotal	0.052	154.52	84.25	0.050	147.13	70.70
Residual	−0.001	−3.03	−3.55	0.006	18.18	8.74
Total	0.033			0.033		
Horizontal inequity index (HI_WV_)	0.052			0.056		

OLS=Ordinary Least Square model; probit=probit model; HI_WV_=horizontal inequity index using the indirect standardization approach.

## Discussion

The results of this study show evidence of inequities in public health care utilization that benefit better-off people in the slum areas in urban Hanoi in Vietnam. This is despite several policies of the government of Vietnam that support the poor and near-poor population and promote the implementation of health insurance for all people (14). In the slum areas, our analysis of the distribution of actual need, predicted need, standardized need, and the decomposition index all provide evidence of inequities in the use of medical care that benefit better-off people. In addition, our results indicate that the poor in the slum areas use less public health care services than expected based on their needs. This analysis of HIs in public health care utilization supplements our probit model analysis, which focuses on variables affecting public health care utilization, including sex–age, NCDs, and health insurance. However, the results show that public health care utilization benefits the poor in the non-slum areas, indicating that there is no evidence of an inequity problem in public health care utilization in the non-slum areas. Use of the equity analysis adopted by this study could provide important information for Vietnamese policy makers concerned with equity in public health care utilization, especially for the slum areas in Hanoi.

The probit model estimation shows some key factors that affect the utilization of public health care services. We find that the major predictors of service utilization in both slum and non-slum areas are sex–age, NCDs, and health insurance. This analysis provides evidence that older men and women are more likely to use health care services than younger people. In addition, self-reported NCDs are strongly related to increased public health care utilization. There is no difference by education (except the secondary school group in the non-slum areas) and work status (except the retired group in the slum areas). Although there is evidence suggesting that the rich use health care more than the poor (28, 29), this finding is not consistent across all wealth quintile groups.

We note some limitations of this study. First, the self-reported recall period of one year related to health care utilization in the questionnaires might suffer from the recall bias. Second, the cross-sectional nature of our study prevents interpretation of casual relationships. Therefore, a longitudinal study to monitor and measure health care utilization in terms of inequity in urban areas should be conducted to provide more robust evidence for policy development.

## Conclusion and recommendations

The findings of our study could help identifying targets for policies to improve equity in the use of public health care services in urban areas in Vietnam. We observed HI in health care utilization in favor of the rich in the slum areas, whereas horizontal equity was achieved in the non-slum areas. In particular, in the slum areas, decomposition of the CI demonstrated that self-reported NCDs contributed to push health care utilization in a pro-poor direction. Our results suggest that to achieve horizontal equity in the utilization of public health care services, policy should focus on preventive interventions for NCDs and on the poor population in slum areas. Moreover, even though SHI has been designed as a financial mechanism for reducing inequity in health care utilization; our results show that SHI contributes to pushing utilization in a pro-rich direction. Hence, appropriate policy should be considered to improve the health care access of people in slum areas by using SHI.

About half of the world’s population lived in urban areas by the end of 2008. Two thirds of the world’s population will live in urban areas in the next 30 years (30). Most of the global urban population will grow in the cities of developing countries, including the ASEAN countries (31). Rapid urbanization will create major challenges for the health care system, particularly when the burden of NCDs is increasing. Hence, setting up an appropriate system to monitor inequity in health care utilization, particularly on utilization related to NCDs, over time may help to understand the impact and implications of policies in the health care sector. These monitoring systems may help ASEAN countries keep track of their progress toward UHC.

## References

[1] Wagstaff A, van Doorslaer E, Paci P (1991). On the measurement of horizontal inequity in the delivery of health care. J Health Econ.

[2] van Doorslaer E, Wagstaff A, van der Burg H, Christiansen T, De Graeve D, Duchesne I (2000). Equity in the delivery of health care in Europe and the US. J Health Econ.

[3] O’Donnell O, World Bank, World Bank Institute (2008). Analyzing health equity using household survey data: a guide to techniques and their implementation.

[4] Vikum E, Bjorngaard JH, Westin S, Krokstad S (2013). Socio-economic inequalities in Norwegian health care utilization over 3 decades: the HUNT Study. Eur J Publ Health.

[5] Gundgaard J (2006). Income-related inequality in utilization of health services in Denmark: evidence from Funen County. Scand J Public Health.

[6] Bago d’Uva T, Jones AM, van Doorslaer E (2009). Measurement of horizontal inequity in health care utilisation using European panel data. J Health Econ.

[7] Goddard M, Smith P (2001). Equity of access to health care services: theory and evidence from the UK. Soc Sci Med.

[8] Vasquez F, Paraje G, Estay M (2013). Income-related inequality in health and health care utilization in Chile, 2000–2009. Pan American J Pub Health.

[9] Barraza-Llorens M, Panopoulou G, Diaz BY (2013). Income-related inequalities and inequities in health and health care utilization in Mexico, 2000–2006. Pan American J Pub Health.

[10] Ministry of Health of Viet Nam, Health Partnership Group (2008). Joint annual health review 2007.

[11] Ministry of Health of Viet Nam, Health Partnership Group (2013). Joint annual health review 2013: towards universal health coverage.

[12] Ministry of Health of Viet Nam, Health Partnership Group (2011). Joint annual health review 2011: strengthening management capacity and reforming health financing to implement the five-year health sector plan 2011–2015.

[13] Vietnam General Statistics Office (2011). Migration and urbanization in Vietnam: patterns, trends and differentials.

[14] Ministry of Health of Viet Nam, Health Partnership Group (2012). Joint annual health review 2012: improving quality of medical services.

[15] Thoa NT, Thanh NX, Chuc NT, Lindholm L (2013). The impact of economic growth on health care utilization: a longitudinal study in rural Vietnam. Int J Equity Health.

[16] General Statistics Office (2004). Vietnam living standard survey 2004.

[17] General Statistics Office (2006). Vietnam living standard survey 2006.

[18] General Statistics Office (2008). Vietnam living standard survey 2008.

[19] General Statistics Office (2011). Vietnam living standard survey 2010.

[20] General Statistics Office http://www.gso.gov.vn/default.aspx?tabid=387&idmid=3&ItemID=9860.

[21] UN-HABITAT (2006). State of the world’s cities 2006/2007.

[22] Lwanga SK, Lemeshow S (1991). Sample size determination in health studies: a practical manual.

[23] World Health Organization (2008). 2008–2013 Action plan for the global strategy for the prevention and control of noncommunicable diseases.

[24] Kolenikov S, Angeles G (2009). Socioeconomic status measurement with discrete proxy variables: Is principal component analysis a reliable answer?. Rev Income Wealth.

[25] Wagstaff A, Watanabe N (2003). What difference does the choice of SES make in health inequality measurement?. Health Econ.

[26] Vyas S, Kumaranayake L (2006). Constructing socio-economic status indices: how to use principal components analysis. Health Pol Plann.

[27] O’Donnell O, van Doorslaer E, Rannan-Eliya RP, Somanathan A, Adhikari SR, Harbianto D (2007). The incidence of public spending on healthcare: comparative evidence from Asia. World Bank Econ Rev.

[28] World Health Organization, Regional Office for South-East Asia (2009). Health inequities in the South-East Asia Region: selected country case studies.

[29] Commission on Social Determinants of Health (2008). Closing the gap in a generation: health equity through action on the social determinants of health: final report of the Commission on Social Determinants of Health.

[30] World Population Prospects: The 2010 Revision and World Urbanization Prospects: The 2011 Revision. http://esa.un.org/unup/unup/p2k0data.asp.

[31] World Health Organization (2010). Why urban health matters.

# Sub article: Policy processes underpinning universal health insurance in Vietnam

**DOI:** 10.3402/gha.v7.24928

**Published:** 2014-09-26

**Authors:** Bui T. T. Ha, Scott Frizen, Le M. Thi, Doan T. T. Duong, Duong M. Duc

**Affiliations:** 1 Department of Reproductive Health, Hanoi School of Public Health, Hanoi, Vietnam; 2 Department of Arts and Sciences, New York University Shanghai, Shanghai, China

**Keywords:** universal health insurance, universal health coverage, policy process, Vietnam

## Abstract

**Background:**

In almost 30 years since economic reforms or ‘renovation’ (*Doimoi*) were launched, Vietnam has achieved remarkably good health results, in many cases matching those in much higher income countries. This study explores the contribution made by Universal Health Insurance (UHI) policies, focusing on the past 15 years. We conducted a mixed method study to describe and assess the policy process relating to health insurance, from agenda setting through implementation and evaluation.

**Design:**

The qualitative research methods implemented in this study were 30 in-depth interviews, 4 focus group discussions, expert consultancy, and 420 secondary data review. The data were analyzed by NVivo 7.0.

**Results:**

Health insurance in Vietnam was introduced in 1992 and has been elaborated over a 20-year time frame. These processes relate to moving from a contingent to a gradually expanded target population, expanding the scope of the benefit package, and reducing the financial contribution from the insured. The target groups expanded to include 66.8% of the population by 2012. We characterized the policy process relating to UHI as incremental with a learning-by-doing approach, with an emphasis on increasing coverage rather than ensuring a basic service package and financial protection. There was limited involvement of civil society organizations and users in all policy processes. Intertwined political economy factors influenced the policy processes.

**Conclusions:**

Incremental policy processes, characterized by a learning-by-doing approach, is appropriate for countries attempting to introduce new health institutions, such as health insurance in Vietnam. Vietnam should continue to mobilize resources in sustainable and viable ways to support the target groups. The country should also adopt a multi-pronged approach to achieving universal access to health services, beyond health insurance.

Vietnam is a socialist country under the leadership of a single party (Communist Party of Vietnam – CPV). In 1986, Vietnam started the reform ‘*Doimoi*’ from planned economy toward a socialist oriented market economy with the aim of unleashing the strength, dynamism, and creativity of its people (1). The policy was fantastically successful, with GDP per capita in real terms increasing from 130 USD (1990) to 1911 USD in 2013 (2). Economic growth has contributed to improving the living standards and the health status of the population; it has, at the same time, facilitated increasing government investment in health care (3).

In the past 20 years, Vietnam has achieved good health outcomes, in many respects with results similar to those of high middle-income countries. Vietnam is among the countries that are on track to achieve different Millennium Development Goals, including reduction in child mortality rates (4, 5). However, there are increasing inequities of health among regions (6).

Recently, the concept of universal health coverage (UHC) was widely advocated by the WHO and other stakeholders. UHC is a system in which everyone in a society can avail the health care services they need without incurring financial hardship (7). There are three main core factors to consider in UHC: who is covered, which service is covered, and the level of financial contribution (8). The UHC required the comprehensive approach with health service delivery, health financing, and political economy and policy processes (9). Although UHC has become a major goal for health reform in many countries, its achievement typically requires an incremental and systematic policy approach.

Although the policies in Vietnam are not consistent with a comprehensive interpretation of UHC, the government started financing curative care through health insurance (HI) in 1992 (10). Over 20 years, four key policy documents on HI were in effect, that is, Decree 299/1992, Decree 58/1998, Decree 63/2005, and Law on HI (2008) and Decree 62/2009 guiding Law implementation. In 2013, the government published its roadmap to achieve universal health insurance (UHI) coverage levels of 70% by 2015 and 80% by 2020 (11).

Although UHI offers a powerful aspirational goal for Vietnam, there are many challenges associated with adopting, achieving, and sustaining it; balancing various political and economic influences is among the most important challenge. In 2012, the coverage of HI in Vietnam was in practice only 66.8% (10), and many shortcomings have been identified in low coverage, lack of basic service package, and insufficient health financing. There is a growing demand to understand the policy levels that can influence the attainment of UHI objectives. This study aims to enhance the HI policy-making processes in Vietnam. This study attempts to describe the UHI policy processes and influential political economy factors influencing UHC in Vietnam.

## Methods

The qualitative research methods (in-depth interview, focus group discussion (FGD), expert consultancy, and secondary data review) were applied in this study. The fieldwork was conducted in two provinces in 2013 (Hanoi and Haiduong) with four FGDs and 30 in-depth interviews. The purposive sampling (snowball technique) was used to identify the key informants (Table 1).

**Table 1:** Key informants

No.	Participants	Working agencies	No.	Methods
1	Policy designers/policy-makers	MOH, VSS	5	5 In-depth interviews
2	Politicians	Government Office, Central Party	2	2 In-depth interviews
3	International partner	WHO	1	1 In-depth interview
4	Administrators	Provincial Health Department and Social Insurance	6	6 In-depth interviews
5	Implementers	Hospitals (public and private), clinics, health insurance agencies	23	2 FGDs and 10 in-depth interviews
6	Users	Commune health centers, provincial and district hospitals, private hospitals	17	2 FGDs and 6 in-depth interviews
	Total		54	4 FGDs and 30 in-depth interviews

A review of 420 documents related to the main policies on HI was conducted, including articles, policies, training materials, media, research, and projects. The research team had sought all identified full-text studies in Vietnamese and English. The research reports could be unpublished, published, or written on HI and UHI in Vietnam from 1990 to 2013. Studies with no data or without published full-text were excluded. The research used electronic databases (PopMed, Ebscohost, Science-Direct) and printed reports of organizations (government or non-government), institutions, or research centers. The research team at the Hanoi School of Public Health, supported by a World Bank consultant, carried out the analysis.

All the interviews/FGDs were conducted in Vietnamese. All interviews/FGDs were recorded and transcribed. Content analysis was applied. The data were analyzed with the support of NVivo 7.0 (12). The conceptual framework, drawing on Walt and Gilson's policy triangle on content, context, and actors in health policy processes underpinned the analysis (13). Analysis was done in Vietnamese and the report was written in English.

Discussions with, and validation by, all research partners and key policy-makers in Vietnam were used to ensure the reliability and validity of findings. Ethical approval was obtained from the Institutional Review Board of Hanoi School of Public Health.

## Results

The results of the HI policy process are described in two main parts: the first HI policy, and the following policies. The first part presented the initiation and piloting of the HI policy. The following part described HI policy adoption, expansion, and roadmap to UHI policy (Fig. 1).

**Fig. 1:** Timeline of key health insurance processes in Vietnam.

### 1990s: Agenda setting and development of the first HI policy in the context of non-experience and non-precedence

#### Context

In Vietnam, before *Doimoi* 1986, the government provided and funded all health care activities, including preventive and curative care. Vietnam experienced socioeconomic crisis because of the collapse of the socialist system in the Soviet Union and Eastern Europe in the late 1980s, which resulted in the sudden cut of foreign aid which in turn affected the government's ability to fund all health care activities. There was deterioration in the health sector in the early 1990s and decline in funding for all public services (14). After *Doimoi*, Vietnam adopted a market economy policy while retaining socialist features in its government structure. The government promulgated policy on partial hospital fees to sustain the health care system in 1989 and the Ordinance on Private Medical and Pharmaceutical Practices in 1993. The Circular 14/1995/TTLB on partial user fees was introduced in 1994 (15). This pushed the health sector to find additional financial sources to run the health system.

At the same time, some key policies were developed such as the Law on People's health protection (1989) and the Constitution of 1992. The right of all people to health care was identified in the above policies providing the legal basis for integrating UHI with national support and resources.

#### Policy development/actors

The CPV had a strong leadership and stewardship toward HI in Vietnam, especially the Commission of Central Propaganda. There was much concern about the poor financial resources of the health system. Dr. Tran Khac Long from the Health Department at Central Propaganda was sent to the Ministry of Health (MOH) to respond to the health financial project. Dr. Long had reviewed the documents (Bismarck model in health care). The documents were from his training materials in East Germany. According to Dr. Long's proposal, HI is one of the options to organize health financing system in reaching UHC to many developed countries where the set of basic health care services is accessible to all, irrespective of income or social status (16).

This HI should be for people and by the people. This was very new – nobody understood about HI. Now, government must pay for HI, employer must pay for the employees and employees must pay. So, this was a very new concept. (HN-VSS-developer)

Between 1989 and 1992, voluntary non-commercial HI was piloted by Dr. Tran Khac Long from MOH and health managers in Quang Tri province and eight districts in the context of the public resources met only 30% of the health care needs and out-of-pocket (OOP) was high at 70%. The project was labeled ‘The State and People working together’. Children and elderly were the main target groups. The program demonstrated that poor local people could share the costs with the health care sector by contributing products such as rice and sugarcane as premium payment for purchasing equipment and medicines.

Lessons learned from the pilot project were used in the development of the first draft Ordinance on HI in 1991 led by the MOH. However, the draft was not approved by the National Assembly (NA) because of concerns over its financial feasibility. Later, Dr. Long and the MOH revised this content within Decree level (the level of Decree is lower than Ordinance, and does not have to be submitted to NA for approval) and submitted it to the office of the Prime Minister (PM). The PM supported the Decree and immediately approved the HI Decree in 1992, just 3 months after submission. The HI head office, which was established right after the Decree approval, played a key role in implementing the HI policy.

#### Content of Decree 299/1992

The HI was started compulsory for civil servants and pensioners as the easiest way to apply the policy. The premium for HI accounted for 3% of salary. The service package included both outpatient and inpatient services.

#### Policy implementation/actor involvement

Fund deficit was observed in 20 provinces during initial implementation of Decree 299/1992, especially in poor provinces and those with high number of pensioners. High expenditure on drugs and a high length of stay for inpatients was reported. These problems were partly resolved by MOH later, when policies on fees for services (Circular 14/1995) and ceiling fees (Inter-ministerial Circular No. 17/1997) were passed.

During the implementation of the Decree 299/1992, much support came from several international partners such as Intensive World Health Organization Cooperation, Swedish International Development Agency, Information System Securities Awareness, and International Labor Organization on strengthening capacity of MOH on HI delivery programs. The support was mainly related to improving human resource capacity such as training on HI, seminars on planning, financial management, and other related activities.

### From 1998 to present: UHI policy process: incremental processes with learning-by-doing approach

#### Context

In order to advance the market economy and increase the resources used for the public sector, the CPV and the government implemented several policies on social mobilization, decentralization, and autonomy. These policies allow the public health sector to raise the necessary resources to provide better equipment and infrastructure, and increase service availability in the health care system. However, these also contributed to increasing inequity of UHI coverage and distributing resources across the different pools.

In this time, the growth of private sectors was gradually increasing. The private beds accounted for 3% of total beds and 1.4% of total autonomous hospitals by 2012 (15).

The financial sources for health care are from different channels: general taxation, social HI, and out-of-pocket payment. There are two major public financial sources that supply funding to health care, namely the state budget allocated directly to service providers, through the MOH and the flow from the social HI fund. Of the total government budget, 93% is for recurrent spending and only 6.85% is meant for spending for development, including equipment and construction. Recently, the government has increased state budget on health from 5.22% (2005) to 8.7% (2010) and 9.4% (2012) (15). Economic growth, while instrumental in supporting the expansion of coverage, also has significant influence on the institutional arrangements for HI delivery. Commitment of the Vietnam government to ‘poor reduction’ during 2000–2010 also played a key role in the UHI coverage.

#### Content

The Vietnam government gradually expanded the service package of HI. The pathway that the Vietnam government applied since 1998 was one of providing HI for the whole population with a larger basic benefit package, funded through a range of financing mechanisms, with the poor and the ethnic people exempted from payment. The service package was gradually expanded (17). There was no limitation in the package covered by HI on drugs, tests, diagnostic imaging, or surgery and medical procedures (17, 18).

#### Policy process/actors involvement

The development of HI policies was identified as a response to the major shortcomings encountered during the implementation of previous policies, including low coverage, lack of basic service package, HI fund deficits, and lack of effective financing mechanisms. The government through the Health Care Fund for the Poor provided the premium payments for the poor and near poor (Decision No. 139/2002), which contributed sharply to increasing coverage. Before 1995, the coverage of HI was low. The coverage of HI reached 5.94% by 1994 (19). By 2012, HI coverage reached 66.8% (20).

By 2005, observing the rapid increasing HI coverage, the CPV set goals of UHI by 2010 (Resolution 46/2005). The roles of HI were recognized as important tools to ensure health care equity as well as important financial sources for health service delivery. The role of the private sector in the health care system was also recognized from 2005.

The lesson was HI could not be successful if it was implemented in a small scale. HI should be implemented in the whole nation. (HN-MOH-policymaker) There should be UHI. This is the mean to ensure the equity, efficiency and development. (HN-politician)

MOH played a significant role in the policy process. The department of HI (which was established in 2005 on the basis of separation from Vietnam health insurance office-VHI) was responsible for developing the policy/law. Several actors have been involved in the development of these policies, including the related ministries, government offices, provincial health departments, and international development partners (mainly World Bank, WHO, and UNICEF). Consultation took place through roundtable meetings and workshops. Users and implementers at the grassroots level were not invited for the debates. Problems from the previous implementation were discussed to be resolved in the agenda setting of the next policy process.

The debates and comments during the development process centered on several issues, including 1) complicated procedures for HI patients; 2) the need for reimbursement in several categories, such as infertility; 3) the need for having a basic, predetermined, benefit package; 4) concerns about the low quality of services at lower levels of the health system and at private facilities; 5) the name of the law; 6) the co-payment rate; and 7) premium rate.

According to key informants, constraints surfaced during the drafting process, including the lack of agreement among and varied perspectives of policy-makers, and the limited time for comments.

The NA members tended to demand lots of benefits, such as that the benefit package not be fixed and the premium reduced, and patients able to go directly to central level facilities to see specialists without being referred by the lower level. But this is not what we mean by ‘social HI’. (HN-international partner)

The department of HI of MOH finalized the draft policy according to comments received, and it was submitted to the government office and the NA for approval. MOH explained to NA members the reasons to revise or not revise the content based on evidence from the facts. The process centered on the development of Law on HI, with 25 versions of draft law before the final bill was approved by the NA on 14/11/2008.

#### Implementation

Funds deficit were evident since the implementation of first policy on HI (Decree 299/1992) and mainly associated with the fact that HI was voluntary, adverse selection problems, the lack of ceiling costs for HI services, the abuse of medical services without co-payment, an ambiguous medical service package, and the increasing costs of medical services.

Fee-for-service was still the predominant payment method. The capitation provider payment mechanism was piloted in four provinces in 2005 with the implementation of Decree 63/2005. Later, this method was expanded to the district level. However, there was much criticism over its inconsistent application among provinces, inappropriate guidelines for capitation payment, and fund deficits at the district level.

It was so hard to for health manager to implement HI policy at district level. Lack of detail guidelines for capitation payment makes us stress. (HD-FGD-manager at district level)

## Discussion

The UHI process in Vietnam has come a long way from its establishment in 1992 to the establishment of a roadmap to get to 70% coverage by 2015 and 80% by 2020. The implementation of UHI is in progress with challenges. The overall policy-making process is incremental with a ‘learning-by-doing’ approach, and reflects the influence of a number of political economy factors.

### Incremental policy processes with learning-by-doing approach

The first policy on HI (Decree 299/1992) was adopted at a time when there was very little policy capacity in the Vietnamese health system with respect to HI. The financial sources from HI were not considered as important and premium rate was set without any detailed financial forecasts. Most policies used some form of evidence, whether in the form of lessons learned from other countries, from pilot project evaluations, or from the results of initial rounds of policy implementation in HI programs, with revisions made to amend shortcomings on an ongoing basis. These small steps paved the way for some major policy changes (21). However, evidence from policy studies or implementation researches was rare. The advantage is that the policy would fit with the low economic development context of Vietnam. However, the disadvantage is non-scientific evidence may limit the radical change in policy content. UHI policies in Vietnam focus more on coverage than they do on the service package and financial protection. As a result, the implementation of UHI is in progress with challenges concerning the lack of essential health services, insufficient financing payment, and fund deficits. These shortcomings must be addressed if Vietnam is to achieve UHC in a comprehensive manner.

The findings clearly showed the processes of discussion, negotiation, contestation, and consensus among different stakeholders in the policy processes. At the central level, the relationship among ministries is horizontal. All ministries are under the stewardship of the NA, government, and CPV in agenda setting. Within each sector, there was a vertical relationship between the central, policy-making level and the implementation level. Overall, these actors, at both the central and the implementation levels, have strong voice and power that could influence the HI policy development and implementation. The voice of other actors, including media, international agencies, and users is still limited because of not being invited to the policy development process. Yet, the policy processes remained, for the most part, closed, with inputs solicited ‘by invitation only’.

### Influence of political economy factors, progressive universalism toward UHI

The HI processes in Vietnam reflect the strong influence of political context factors. The right of all people to health care was identified in a number of policies after *Doimoi* providing the legal basis for national support of UHI. CPV leadership plays a key role in the development of UHI.

There are two pro-poor pathways to achieving UHC within a generation. First, HI would cover basic health package/intervention to achieve convergence, health problems. The second pathway provided a larger benefit package, funded through a range of mechanisms, with the poor exempted from payments (22). Vietnam applied the first approach in the early stage of policy process and applied the second type of progressive universalism in the HI policies from 2005. The advantage of the second approach is that a wide range of health services can be offered, the non-poor are engaged in a prepaid mandatory scheme, and the poor can access health services easier without paying user fees. The situation of Vietnam is similar to that of Rwanda (22). Vietnam is moving to UHI through mandatory insurance and co-payment with exemption for the poor and ethnic people.

Similar to other countries that applied the second type of progressive universalism, Vietnam faced disadvantages (23). It was reported that there was increasing provision of unnecessary services to patients, higher out-of-pocket spending on hospital care, and higher spending per treatment episode, affecting the quality of services and increasing the risk of capitation fund deficit at lower level facilities due to autonomy reformed policies which were first introduced at the district and provincial levels since 2005 (18, 23, 24). These problems were solved via incremental actions such as capitation methods applied at the district and the commune levels, and accepted co-payment for advanced quality services/or treatment at provincial and central levels.

The economic recession in recent years has also led to a slowdown of government financial contributions to the health sector, especially in government bonds for investment in hospitals, and in the limited quality of and accessibility to services. This will affect the desirability and affordability of HI programs in the eyes of both enterprises and individuals, and slower expansion of coverage levels can be expected in the coming years. In summary, it can be seen that the government was looking for market-oriented ‘instruments’ of policy in the new context, while retaining or expanding equity goals.

## Conclusions

The review of the policy processes of UHI over 20 years has shown that Vietnam has attained considerable coherence in the process of developing a comprehensive HI system that contributes to a sustainable and equitable health sector. The results also suggest that policy processes of UHI are mainly taking an incremental, ‘learning-by-doing’ approach with the involvement of a complex array of actors.

The government lays more emphasis on increasing coverage than service package expansion and financial protection. Political economy factors have a significant influence on VHI-related policy processes. In the presence of: 1) decentralization and 2) a prevailing socialist ideology and practice of socialization of health services and autonomy, the disparities both in terms of outcomes and implementation processes have increased. Considerable challenges remain, as achieving high coverage in the informal sector and boosting the voluntary purchase of insurance will not be easy.

This study suggests that an incremental, learning-by-doing approach can be effective and necessary in early stage in the context of low economic development of Vietnam. For the future, Vietnam will need to mobilize resources in sustainable and viable ways to support target group expansion and financial protection, as it makes strides to achieve truly comprehensive HI coverage.

Besides, Vietnam should open opportunity for international actions to UHI process through policy and implementation research. The evidence from research can be used for effective designing and implementing of specific pathways for evolution in the policy for UHI implementation.

## References

[1] Doanh LD, Küsel C, Dr Maenner U, Meissner R (2008). Market economy with socialist orientation in Vietnam and the concept of the social and ecological market economy. The social and ecological market economy – a model for Asian development?.

[2] World Bank (2014) (2014). GDP per capita (USD). http://data.worldbank.org/indicator/NY.GDP.PCAP.CD.

[3] Communist Party of Vietnam (2011) Documents of the Communist Party, Congress 11.

[4] UN Inter-Agency Group for Child Mortality Estimation (2011). Levels and trends in child mortality.

[5] WHO, UNICEF, UNFPA, The World Bank (2012). Trends in maternal mortality: 1990 to 2010. WHO, UNICEF, UNFPA and The World Bank estimates 2012.

[6] Ministry of Health (2012). Health related millennium development goals Vietnam 2012.

[7] WHO (2005). Sustainable health financing universal coverage and social health insurance.

[8] Savedoff WD, de Ferranti D, Smith AL, Fan V (2012). Political and economic aspects of the transition to universal health coverage. Lancet.

[9] World Bank (2013). Global conference on universal health coverage for inclusive and sustainable growth: a global synthesis report.

[10] Ministry of Health, Health Partnership Group (2013). Joint annual health review 2013.

[11] Ministry of Health (2013). Approval of proposal on roadmap to achieving universal health insurance 2012–2015 and 2020.

[12] Ritchie J, Spencer L, Bryman A, Burgess R (1994). Qualitative data analysis for applied policy research. Analyzing qualitative data.

[13] Walt G, Gilson L (1994). Reforming the health sector in developing countries: the central role of policy analysis. Health Policy Plan.

[14] Ensor T (1995). Introducing health insurance in Vietnam. Health Policy Plan.

[15] Ministry of Health, Health Partnership Group (2012). Joint annual health review: improving quality of medical services.

[16] Carrin G (2002). Social health insurance in developing countries: a continuing challenge. International security review.

[17] Vietnam National Assembly (2008). Health Insurance Law.

[18] Ministry of Health, Health Partnership Group (2011). Joint annual health report: strengthening management capacity and reforming health financing to implement 5 year health sector plan 2011–2015.

[19] Vietnam Institution for Health Policy and Strategy (2007). Report on 15 years implementation of HI from 1992–2007. Vietnam Institution for Health Policy and Strategy.

[20] Ministry of Health, Health Partnership Group (2012). Roadmap to achieve Universal Health Insurance in Vietnam.

[21] Buse K, Mays N, Walt G (2005). Making health policy. Understanding public health.

[22] Jaminson DT, Summers LH, Alleyne G, Arrow KJ, Berkley S, Binagwaho A (2013). Global health 2035: a world converging within a generation. Lancet.

[23] Wagstaff A, Bales S (2012). The impact of public hospital autonomization: evidence from a quasi- natural experiment.

[24] London JD (2008). Reasserting the state in Vietnam health care and the logics of market-Leninism. Policy Soc.

# Sub article: Has decentralisation affected child immunisation status in Indonesia?

**DOI:** 10.3402/gha.v7.24913

**Published:** 2014-08-25

**Authors:** Asri Maharani, Gindo Tampubolon

**Affiliations:** 1 Medical Faculty, University of Brawijaya, Indonesia; 2 Institute for Social Change, University of Manchester, United Kingdom

**Keywords:** Fiscal decentralisation, immunisation status, Indonesia, multilevel model, multiple imputation

## Abstract

**Background:**

The past two decades have seen many countries, including a number in Southeast Asia, decentralising their health system with the expectation that this reform will improve their citizens’ health. However, the consequences of this reform remain largely unknown.

**Objective:**

This study analyses the effects of fiscal decentralisation on child immunisation status in Indonesia.

**Design:**

We used multilevel logistic regression analysis to estimate these effects, and multilevel multiple imputation to manage missing data. The 2011 publication of Indonesia's national socio-economic survey (Susenas) is the source of household data, while the Podes village census survey from the same year provides village-level data. We supplement these with local government fiscal data from the Ministry of Finance.

**Results:**

The findings show that decentralising the fiscal allocation of responsibilities to local governments has a lack of association with child immunisation status and the results are robust. The results also suggest that increasing the number of village health centres (*posyandu*) per 1,000 population improves probability of children to receive full immunisation significantly, while increasing that of hospitals and health centres (*puskesmas*) has no significant effect.

**Conclusion:**

These findings suggest that merely decentralising the health system does not guarantee improvement in a country's immunisation coverage. Any successful decentralisation demands good capacity and capability of local governments.

In the past 20 years, decentralisation has been considered by many policy-makers to be an important strategy to improve the performance of health systems, including those in South East Asian countries such as Thailand (1, 2), Malaysia (3), The Philippines (4), Vietnam (4), and Indonesia (5). The expectation of these policy-makers is that in the health sector, decentralisation will improve efficiency, service delivery innovation, quality, and equity in healthcare, which in turn will improve the health status of the population (6, 7). Under decentralisation, central government devolves responsibility for health service delivery to local governments, including the authority to carry out local planning, procurement of equipment, financing, and evaluation (8–11).

Based on the type of authority devolved, decentralisation is made up of: political, administrative, and fiscal (12, 13). Political decentralisation refers to the degree to which central government allows local governments to undertake the political functions of governance. Administrative decentralisation transfers the administration and delivery of public services from central to local governments, while fiscal decentralisation is designed to increase local government control of revenue. As fiscal decentralisation should result in expenditure being matched more closely to local needs and preferences, the expectation is that local government will increase the efficiency of public service provision while at the same time increasing the promotion of accountability (14). Furthermore, fiscal decentralisation allows local governments to raise revenue, for example, through their ability to tax or to receive grants (15). The focus in this paper is on fiscal decentralisation as this is the most important step in the overall decentralisation process (16).

Previous studies, both multi-country and single-country, have identified a positive association between fiscal decentralisation and health outcomes. For example, a study which used panel data from a number of low- and high-income countries found that fiscal decentralisation was inversely related to infant mortality rate and concluded that the marginal benefit obtained by fiscal decentralisation is greater for low-income countries (17). It also argued that economic development in low-income countries increases the institutional capacity of local authorities faster than that of central government. A number of single-country studies have presented similar results. For example, a study using a large panel data of Argentine provinces demonstrated a negative relationship between fiscal decentralisation and infant mortality rates (18). Another study using an index of fiscal decentralisation based on spending and revenue measures for rural villages in India concluded that decentralisation reduces infant mortality rates and that the effectiveness of fiscal decentralisation is commensurate with the degree of political decentralisation (16). It also mentioned the role which local authority capacity plays in the successful utilisation of a decentralised budget. Finally, comparable results highlighting the relationship between fiscal decentralisation and infant mortality rates have been presented in recent studies of China (19), Spain (20), and Colombia (21).

This evidence on the association between fiscal decentralisation and health outcomes tends to be based on aggregate analysis, with district and country as the units of analysis. However, it is well-known that such analysis risks the invalid transfer of aggregate results to individuals (22). This risk may result in biased inference due to loss of information when using ecological correlations as a replacement for individual correlations. We decided therefore to use a multilevel model, and our study contributes to the existing literature by distinguishing individual as well as local government determinants in our analysis. By accounting for this multilevel structure of individuals within districts, we were able to investigate whether the effect of local government conditions on individual health outcomes varies between local governments. This meant that the effect of fiscal decentralisation on individual health outcomes could thus be tested appropriately. Moreover, by combining contextual and individual determinants, we are able to examine the effect of local government fiscal capacity on the promotion of health status in Indonesia.

The aim of this study was to examine the consequences of fiscal decentralisation specifically on child immunisation status in Indonesia. The reason for this focus was firstly because immunisation is accepted as a proxy for similar public services, such as family planning and other preventive services. Furthermore, immunisation is the most cost-effective health intervention in terms of reducing both the morbidity of vaccine-preventable diseases and the child mortality rate (23, 24), increasing the significance of the general effects of decentralisation and health reform (25–27).

Evidence of the effect of decentralisation on immunisation status across countries presents various outcomes. In India, for example, a study in Kerala showed decentralisation resulted in improved access to immunisation programmes and increased Diphtheria, Pertussis, Tetanus (DPT) immunisation coverage (28). The reasons were found to be improved infrastructure (including facilities and equipment) in Kerala's healthcare institutions, and better accountability in the public healthcare system. In contrast, studies of Papua New Guinea have revealed a decrease in Bacille Calmette-Guérin (BCG) immunisation coverage among children under 1 year following decentralisation. Similarly, an immediate evaluation of decentralisation in Tanzania's Expanded Programme on Immunization (EPI) found services at district level to be of poor quality. Reasons for this included inadequate cooperation between central and local policy-makers, demoralised health service providers, a reduced number of supervision visits by EPI staff, and the improper maintenance of vaccine temperature (29). Interestingly, a cross-country study found that decentralisation has different effects in low- and middle-income countries. Decentralised low-income countries were found to have higher measles and DPT3 immunisation coverage than centralised ones, while in contrast decentralised middle-income countries have lower immunisation coverage for the same period (7).

The potentially negative effect of decentralisation on Indonesia's immunisation status has been discussed by international organisations working in health and immunisation, such as USAID and GAVI (30, 31). They have commented on the stagnation of immunisation coverage in Indonesia during the previous decade, suggesting that decentralisation has contributed to it. Before decentralisation, the government of Indonesia paid considerable attention to improve coverage of basic childhood immunisation against polio, measles, diphtheria, tetanus, pertussis, and tuberculosis. In 1977, it officially initiated EPI activities which provided basic, free immunisation for all children. Unlike most of Indonesia's maternal and child health services, this national immunisation programme was not fully decentralised. Instead the responsibility for the supply and cold chain maintenance of vaccines was retained by central government, while that for the provision of the health facilities, health professionals, and equipment needed to carry out vaccination was devolved to district governments (32). This division of responsibility has led in some cases to uncertain programme ownership (possibly exacerbated by differing priorities at local level) and has almost certainly played a part in the stagnation of immunisation coverage since decentralisation (30).

A study examining 10 districts of Java found that there has been no improvement in DPT3 immunisation coverage since decentralisation, despite the significant increase in public health expenditure. One reason for this failure is the limited analytical and planning capacity of local government representatives, who were not provided with the education and training needed to plan and implement their new areas of responsibility. The failure of decentralisation to improve child immunisation in Indonesia is not in dispute; the need to address this is urgent, and the first step is to examine the consequences of decentralisation on immunisation status.

Indonesia constitutes a particularly interesting case, not only because of the size of the country but also because of its remarkable progress in creating a decentralised system of government in a relatively short period of time. Starting in 2001, Indonesia devolved responsibilities from central to district government in almost all government administrative sectors (including health) with the aim of improving efficiency, quality, and equity of public service provision (4). Evaluating the consequences of decentralisation in Indonesia also provides lessons for other Southeast Asian countries, especially those with similar reform and reform backgrounds. Like other Southeast Asian countries, decentralisation of the health sector in Indonesia was launched in the late 1990s (before general decentralisation in 2001) following the 1997 East Asian financial crisis (4), and has wider implications throughout Southeast Asia, whose countries face a similar epidemiological challenge of tropical infectious diseases among children.

Two studies in particular have found that health sector decentralisation in Indonesia has failed to achieve its aim, and they highlight several plausible explanations for this failure (33, 34). The first explanation is that local governments only have real discretion for less than 30% of their health expenditure (33), a figure which is low compared to the average of local expenditure autonomy (58%) experienced in other developing countries (14). Another explanation is the limited capacity of local governments, which are given responsibility for funds after decentralisation but not the skills needed to utilise them appropriately (34, 35). Unlike Thailand (possibly the most successful example of decentralisation in Southeast Asia) (1), local authorities in Indonesia are not required to demonstrate sufficient capacity and commitment before receiving greater autonomy under decentralisation.

There are a number of studies which evaluate the consequences of decentralisation in Indonesia. However, their usefulness is limited by the fact that they only cover some of Java's districts. There are nearly 500 districts across Indonesia, and those outside Java tend to be poorer. Omitting districts on remote islands means these studies capture only a partial picture of the country. This adds urgency to the need to evaluate the effects of decentralisation in all districts in Indonesia, and specifically its association with child immunisation. Our study has used data sourced from multiple surveys (contextual, household, and individual) in 497 districts.

## Data and methods

### Data

This study combines data from various sources. The Indonesian national socio-economic survey (*Survei sosial ekonomi nasional*, or Susenas) in 2011 was the main source of household-level data. It provided information on a child's immunisation status as well as the characteristics of the mother and the socio-economic status of the household. Alongside Susenas, we assembled data from the 2011 national village census (*Potensi desa*, or Podes) and government fiscal information. Podes provided information on population and the number of health facilities in all villages within a district, the aggregate of which is calculated for each district. We included health facilities which provide immunisation for children: hospitals, public health centres (*Puskesmas*), and integrated health services posts (*Posyandu*).^1^ The government fiscal data was obtained from the Ministry of Finance. We linked the Susenas data to the other data sources using district codes. Taken together this data captures the nested structure of households by district.

### Immunisation status measure

The key outcome variable is complete immunisation status among children aged 12–23 months. We extracted the data on immunisation status from Susenas, in which parents are asked whether their children received each of the basic immunisations or not and the number of doses received for each. Although every immunised child receives an immunisation card recording the date of immunisation and how many they have received, the parent was not obliged to show this card to the Susenas researcher. The data were created based on the answers of the parents. We define complete immunisation status based on a child receiving each of the immunisations in the national EPI schedule (36).^2^ Children above 2 years old are not included in this study to avoid confusion with the immunisation booster schedule.

**Table 1:** Schedule of Indonesia routine immunisation

Age of administration	Antigens		
0 month	BCG	HB0	OPV0
2 months	DPT1	HB1	OPV1
3 months	DPT2	HB2	OPV2
4 months	DPT3	HB3	OPV3
9 months	Measles		

We measured child immunisation data as a binary variable (1=received complete basic immunisation; 0=not received complete basic immunisation) – complete basic immunisation is important to protect children from vaccine-preventable diseases. Incomplete immunisation (e.g. a child receiving only two shots of DPT immunisation from a series of three) means that immunity is not completely formed. The Indonesia government emphasises the importance of complete basic immunisation to eradicate these diseases and to reduce child mortality rate (37).

### Fiscal decentralisation measure

We measured fiscal decentralisation using the ratio of local public expenditure on health to total local public expenditure, and found that this measurement reflects responsible governance at the local level. The most common measure of fiscal decentralisation is the local share of total government expenditure (16, 17, 19). However, this measure conveys only a limited reflection of fiscal decentralisation, as it fails to consider the control which local authorities have over funds raised locally or other local potential resources (21, 38). A study in Colombia extended these measures by using the ratio of locally controlled health expenditure to total health expenditure. However, this measure is less suitable for the case of decentralisation in Indonesia, where local governments received funds from central government in a bulk called the balancing fund (*dana perimbangan*). It includes a general grant (*dana alokasi umum*), shared taxes, natural resource revenue shares, and a special allocation grant channel (*dana alokasi khusus*) (39). Although the transfers from central government to local government remain the dominant means of financing, earmarking is gone, and local governments have the authority to allocate the funds for each public service sector, including health. We therefore decided to use a fiscal decentralisation measure which represents the resources used to finance the health sector over all resources for which local governments have authority and also discretion on how to use these resources. We consider this measure more useful, as it captures the willingness of local governments to allocate their funds for the health sector.

### Household-level determinants

Determinants at household level consist of birth attendants, mothers’ employment status, mothers’ age, mothers’ education, and household socio-economic status. We created a dummy variable for birth attendants (1 for a child whose birth was attended by health professional – physicians, midwives and nurses – and 0 for a child whose birth was not). Employment of mothers is measured using a dummy variable (1 for employed and 0 for unemployed). We classify mothers’ age into three levels: ≤20 years, 21–30 years and>30 years, and measure their education according to the highest level of education attained, differentiated into three levels: primary, secondary, and tertiary education. Household socio-economic status is measured using household expenditure over 1 year. Household expenditure variable is entered as a log-transformed continuous variable to make the distribution more symmetric and to reduce the effect of outliers.

### District-level determinants

We used a number of determinants which measure variation in local health provision to examine contextual effects. First, we took the number of health facilities per 1,000 population to measure the availability of healthcare providers, especially in regard to immunisation services (hospitals, health centres, and village health posts). We also used the proportion of urban population, population density, and gross domestic product (GDP) per capita as district-level determinants. Similar with household expenditure variable, we entered GDP per capita as a log-transformed continuous variable.

## Methods

Our study used a multilevel logistic regression model (which we believe to be most appropriate because it considers the nested structure of households within districts), and estimated the association of fiscal decentralisation with child immunisation status in Indonesia, treating the dependent variable as binary (complete immunisation or not). The first level comprised household characteristics and district characteristics made up the second level. Considering households *i* nested in districts *j*, the model is:

$$E_{ij}*=\gamma_{00}+\Sigma \gamma_{0j}W_{j}+\Sigma \beta_{ki}X_{ij}+u_{0j}+\varepsilon_{ij}$$

with:

*E*_ij*_=logit(*P*(*E*_*ij*_=1)),

*W*_*j*_ is a set of district characteristics,

*X*_*ij*_ is a set of household characteristics,

*u*_0*j*_ are the random intercept varying over district γ_00_ with mean zero and variance $\sigma_{00}^{2}$,

ɛ_*ij*_ is normally distributed with mean zero and variance $\sigma_{\varepsilon }^{2}$.

### Missing data

Where there were missing data, we obtained multilevel multiple imputed values, which avoided the potential bias which can arise when incomplete data is mishandled (when cases are deleted, or when indicator variables are used for missing data). This also made full use of the observed data, since missing data appeared at both household and district level. Multilevel multiple imputation under missing-at-random assumption was used to estimate missing data for complete immunisation status and covariates (40). We used all predictors taken together to impute the missing values and analyse the imputed data.

## Results

We begin by describing immunisation status and characteristics of both households and districts, and then present the results of the multilevel analysis of predictors of child immunisation status. The descriptive statistic (see Table 2) shows that almost half of the children in the survey did not receive complete immunisation. This means that more than a decade after decentralisation, Indonesia is missing the WHO immunisation target of 80% and thus failing to provide basic primary care services. However, this national figure masks huge variation across districts. Fig. 1 highlights this, showing that more than four out of every five children in 57 districts were covered with complete immunisation, while in 50 other districts less than one in every five children was covered. A sense of the importance of area variation in immunisation coverage can be gained from the map in Fig. 2, which highlights geographical disparities across districts and compares district attainment of DPT3 immunisation coverage in Indonesia to that of selected countries in Southeast Asia region. Overall, DPT3 coverage in Indonesia is far below that of Thailand and Singapore (often presented as examples which have over the last two decades performed well compared to other countries in the region), and performed slightly worse than the Philippines and Laos (Laos performed well below other Southeast Asia countries before the 1990s, but by 2011 it had improved significantly and performed better than Indonesia). Within Indonesia, we observed an immense variation of DPT3 coverage between districts. The three districts of Kupang, Gorontalo, and Jembrana achieved a notable public services performance, with the same DPT3 coverage as Thailand and Singapore, while at the other end of the scale, almost all the children included in the Susenas survey in Mappi, Aceh Timur, Yapen, and Nagan Raya missed complete immunisation. This wide gap of achievement between districts necessitates analysis at district and individual levels, not at national level.

**Fig. 1:** Spatial distribution of immunisation coverage among districts in Indonesia.

**Fig. 2:** DPT3 coverage in Indonesia and selected comparators (1985–2011) and comparison with Indonesia district attainment (2011).

**Table 2:** Descriptive statistics on household and district characteristics

	Mean (%)	SD	Missing (%)
Household characteristics			
Complete immunisation status			0.17
Child receive complete immunisation	53.36%	23.07	
Child not receive complete immunisation	46.64%		
Residential areas			0
Rural	61.55%		
Urban	38.45%		
Birth attendants			0
Non-health professional	24.31%		
Health professional	75.69%		
Mothers’ age			0
20 years	6.18%		
21–30 years	52.69%		
> 30 years	41.13%		
Mothers’ education			4.19
Primary/no education	60.56%		
Secondary	28.06%		
Higher	11.39%		
Mothers’ employment status			0.01
Unemployed	59.69%		
Employed	43.01%		
Household income (IDR 1,000)	2406.27	2456.86	0
District characteristics			
Local health expenditure as a proportion of total expenditure (%)	9.53	3.29	1.50
Hospitals/1,000 population	0.03	0.03	1.99
Health centres/1,000 population	0.23	0.20	1.99
Village health posts/1,000 population	1.39	0.60	1.99
Proportion of urban population	0.39	0.30	0
Population density	1058.98	2525.54	0
GDP per capita (IDR 1,000)	19986.8	32881.2	0
Number of children	23,766		
Number of districts	497		

A similar variation occurs in the percentage of local government expenditure allocated for healthcare, and indicates different levels of concern for the health sector (Fig. 3). Five districts prioritise health and allocate more than one-fifth of their expenditure for health, while some districts allocate less than 5%. An indication of the different capacities of local authorities to manage their health budget is shown by their utilisation of it. Less than half of all districts used all of their health budget. More than a 100 districts used less than half of their health budget and three districts leave more than 70% of their health budget unused. Details of the utilisation of this budget are shown in financial flows of local governments (Table 3). On average, most of local government revenue (86.57%) was transferred from central government. Local governments use more than 75% of the money on salaries and other operational expenditure (52.38 and 24.89%, respectively), while the expenditure for investment (facilities and infrastructure) comprises only less than 25% of total local government expenditure. This is expenditure which has the potential to contribute to an improved public health outcome, although as is clear from the literature, this is not guaranteed. Modelling the association of fiscal decentralisation with immunisation status was done next.

**Fig. 3:** Health budget and expenditure across districts in Indonesia 2011.

**Table 3:** Financial flows at district level 2010 (in percentage)

	Mean	SD	Min	Max
Revenue				
Own-source revenue	6.58	8.03	0.19	68.69
Transfer from central government	86.57	9.85	25.82	99.6
Transfer from provincial government	3.69	3.16	0	24.43
Revenue from other sources	3.15	4.25	0	21.45
Expenditure				
Salary	52.38	14.23	0	79.81
Other operational expenditure	24.89	6.87	3.89	59.12
Investment	22.39	10.81	0	58.62
Other expenditure	0.32	2.72	0	59.84

Multilevel logistic regression analysis (Table 4) was carried out using three models. The first model included only household-level determinants, while the second and the third models included both household- and district-level determinants. The main district-level determinant included in the second model is local health expenditure as a proportion of total local expenditure, while in the third model determinants are the number of hospital, health centre and village health post per 1,000 population. We used two different models to avoid double counting since local governments also spend their money on these three types of health facility. In addition of these determinants, we included proportion of urban to total population, population density, and log GDP per capita.

**Table 4:** Determinants of child's immunisation status

	Model 1	Model 2	Model 3
Household-level variables			
Residential areas			
(Rural)			
Urban	0.12 (0.04)‡	0.12 (0.04)‡	0.13 (0.04)‡
Birth attendants			
(Non-health professional)			
Health professional	0.43 (0.04)‡	0.42 (0.04)‡	0.42 (0.04)‡
Mothers’ age			
(≤20 years)			
21–30 years	0.12 (0.06)†	0.13 (0.06)†	0.12 (0.06)†
> 30 years	0.13 (0.07)†	0.13 (0.07)†	0.13 (0.07)†
Mothers’ education			
(Primary or less)			
Secondary	0.18 (0.04)‡	0.18 (0.04)‡	0.19 (0.04)‡
Higher	0.30 (0.06)‡	0.29 (0.06)‡	0.31 (0.06)‡
Mothers’ employment status			
(Unemployed)			
Employed	−0.04 (0.03)	−0.03 (0.03)	−0.04 (0.03)
Log households income	0.18 (0.03)‡	0.18 (0.03)‡	0.18 (0.03)‡
District-level variables			
Local health expenditure as a proportion of total expenditure (%)		2.03 (1.57)	
Hospitals/1,000 population			−0.65 (2.24)
Health centres/1,000 population			−0.43 (0.28)
Village health posts/1,000 population			0.54 (0.09)‡
Proportion of urban population		0.52 (0.23)†	0.74 (0.27)‡
Population density		−0.00 (0.00)‡	−0.00 (0.00)†
Log GDP per capita		−0.13 (0.07)*	−0.12 (0.07)*
Between district variance	1.09	1.05	1.01
ICC	0.25	0.24	0.23
Median odds ratio	2.71	2.65	2.61

Note: Reported are marginal effects (standard error). Sig.:

* significant at 10% or less

† significant at 5% or less

‡ significant at 1% or less.

Results from the first model showed that living in urban areas, the presence or otherwise of birth attendant, mothers’ education level, and households’ income are all statistically significant at 1%. Among these household-level determinants, it seems that the effect of having a professional birth attendant is the most influential, with children in this category having 43% higher probability to receive complete immunisation than children whose birth were not, holding all other determinants constants. Turning to other determinants, children who live in urban areas and those of better-off families are more likely to be immunised. Mothers’ characteristics also play an important role in their child immunisation status. Mothers who have only completed primary education or less are less likely to immunise their children than those with a higher level of education, while teenage mothers have a lower probability of immunising their children than older mothers. However, the effect of mothers’ employment status is small and far from statistically significant. Overall, these estimates remain consistent in each of the three models.

In the second model, the results indicate that there is insufficient evidence to reject the null hypothesis that local health expenditure as a proportion of total expenditure is not correlated with immunisation status among children. We check the plausibility of threshold effect by re-parameterising the local health expenditure proportion as tertiles and quintiles (Appendix 1). The test of joint significance indicates that both the tertiles (*χ*^2^=3.21, df=2, *p*=0.2) and the quintiles (*χ*^2^=4.93, df=4, *p*=0.29) of the local health expenditure as a proportion of total expenditure has no significant effect on immunisation status.

In the third model, the results show that increasing the number of village health post by one per 1,000 of the population improves the probability of children receiving complete immunisation by 54%. However, adding a hospital and a health centre has no significant effect. The effects of proportion of urban to total population, population density and the wealth of the district (as shown by GDP) remain consistent in the second and third models. Children living in a district with a higher proportion of urban population have a higher probability of having full immunisation. In contrast, those who live in densely populated districts have a lower probability of receiving immunisation, although the effect is minuscule in size.

As we used a multilevel logistic regression model in this study, we explain the effect between levels using median odds ratio (MOR) (41, 42). The MOR compares two children from two randomly chosen districts. In the first model, for two children with the same household-level determinants, the MOR of the child living in a district with a higher propensity of receiving immunisation to the child living in the district with a lower propensity is 2.71. This is a high odds ratio (41), suggesting that the heterogeneity is substantial. Including district-level determinants in the second and third models reduces the unexplained heterogeneity between districts to MORs of 2.65 and 2.61, respectively, which are still high. Thus, the propensity of children to receive complete immunisation varies a great deal between districts. Furthermore, the results of analysis using multilevel multiple imputed data are reasonably similar, in that they exclude all individuals with missing values (available in Appendix 2). This sensitivity analysis shows that the results are robust.

## Discussion

Indonesia launched decentralisation in 2001, devolving greater authority to local government with the aim of improving the efficiency, quality and equity of healthcare services, with the expectation that this would increase the health status of the population (4, 43). This study evaluates the consequences of fiscal decentralisation on child health by assessing childhood immunisation status across districts in Indonesia. In contrast with findings from other countries (16, 18–21), our results show that fiscal decentralisation has no statistically significant association with child immunisation outcomes. To shed more light on the failure of fiscal decentralisation in Indonesia to achieve its aim, we looked at the flow and utilisation of local government expenditure. Local governments rely on transfers from central government, which account for 87% of all their revenue (Table 3). However, the bulk of local government expenditure is spent on salaries (54%) (the central government has control over district health personnel). This means that local governments only have discretion on over 30% of central government transfer, plus any revenue they are able to raise themselves. Model 2 however showed that increasing this discretion has no bearing on child health outcomes (Table 4).

We thus turned to a different explanation, one of capability. Implementation of decentralisation does not necessarily mean that the decentralised entities can manage the system they are presented with. Several studies in Indonesia highlight the importance of local authority capability, especially in regard to planning, budgeting, and utilising their budget successfully. A study in 10 districts in West Java and East Java provinces discovered that the absence of leadership and vision among bureaucrats at local level meant they continued to implement the old system after decentralisation, rather than responding to the health problems in their area (34). Furthermore, a study of Southeast Sulawesi province reported a district allocating a mere 2% of its budget to the health sector, and that the local authorities in this sector have no planning and budgeting capability. At the same time, none of the budget was allocated for capacity building (44). Similar facts have been presented in studies of West Sumatra (45), Jambi (46), and West Kalimantan provinces (47).

Our results revealed that the ability of local governments to utilise their budget varies enormously, with more than half (57%) of the districts failing to absorb their entire health budget (Fig. 3). Even worse, three districts utilised less than 30% of their budget. Under these circumstances, it is unlikely that local government programmes will perform well. We found a wide variation in immunisation coverage, with some districts performing better and exceeding the WHO cut-off (80%), and others performing much worse. Such variation is difficult to reconcile since all district governments exercised similar discretion over expenditure after decentralisation. We concluded that this difference emphasises the importance of local government capability to manage their budget according to local needs.

There are several ways in which local government could utilise the health budget to improve health status. Increasing the number of village health posts in districts, for example, since immunisation status is found to be positively associated with the number of village health posts per 1,000 population. The district government of Jembrana is recognised as an example of one which has deployed most of its budget and has provided successful innovation in its health services following decentralisation. In 2003 it launched the *Jaminan Kesehatan Jembrana* health insurance scheme, which provides free primary healthcare services for all its citizens, on top of which, to improve the equity of access to healthcare, it provides free secondary and tertiary healthcare services for poor residents (48). Our study shows that immunisation status among the children of Jembrana district a decade after decentralisation was considerably high with 93% coverage, comparable to that of Singapore and Thailand (Fig. 2).

The importance of providing health facilities to improve healthcare is supported by household level findings. Children living in rural areas and poor households are less likely to be covered by complete immunisation, despite the government providing free immunisation services for all children. The real cost of accessing healthcare renders households with low economic status and in rural areas unable to access immunisation services, as transport and opportunity costs are not borne by the government. These costs impose a greater burden on poor households, and negatively affect their healthcare-seeking behaviour (49). This household-level finding supports the district-level findings, namely that a more even distribution of village health posts as one of immunisation providers improves immunisation coverage. Better distribution of immunisation providers decreases the distance to health providers which in turn increases immunisation status among children due to lower financial costs and shorter time needed to get to these providers. Previous studies in Nanggroe Aceh Darussalam revealed that the increasing local budget allocation for health sector has a positive impact on physical infrastructure budgets (50). However, a considerable amount (40%) of budget for the health sector was spent on public hospitals, which mainly provide curative care services (51). Our study finds that among the three types of health facilities (hospital, health centre and village health post), only village health post has significant and positive association with child immunisation status. Village health post provides promotive and preventive healthcare services and located in villages, which is more affordable than other health facilities. The budget for the health sector should be allocated more to increase the number of this type of health facility to improve immunisation coverage.

The main limitation of this study is that the analysis used cross-sectional datasets. Further study using data from several years, both before and after decentralisation, would better capture the consequences of fiscal decentralisation for health outcomes. As this study used multisource data and not all data sources are available annually (for instance, Podes data only available 3 years), a multi-year study needs to consider other data sources. Moreover, the data we used to discuss child immunisation status was based purely on the verbal responses of parents who were not obliged to show an immunisation card, and whose answers regarding the completeness of their children's immunisation may have been influenced by recall bias. Future data collection is needed to improve measurement of individual past experiences.

Despite these limitations, our findings have several important implications. Firstly, this research indicates that districts continue to vary both in terms of immunisation coverage and also in terms of the extent to which local governments take advantage of the opportunities offered by fiscal decentralisation. While earlier studies focus on variation across countries, this study finds variation across districts and within one country, with the suggestion that the consequence of decentralisation on health status are more accurately assessed when districts and children are used as units of analysis. Secondly, this study extends the previous fiscal decentralisation measurements by referring only the resources allocated to healthcare services. One advantage of this measurement is that it better reflects responsible governance at the local level.

## Conclusions

Fiscal decentralisation is often promoted as a strategy to improve the performance of healthcare services, which in turn improve health outcomes, including immunisation status among children. However, the evidence across countries has not been definitive. This study has found that the transfer of fiscal authority to local governments is not a panacea of the problems of how to improve child immunisation status in Indonesia. Merely increasing the health budget at district level is not adequate. A new understanding is made possible here by investigating the regional disparities of public health programmes. The immense variation of immunisation coverage across districts suggests that lessons can be learned from the better-performing districts. Perhaps most significantly, in addition to increasing the discretion of local governments over decentralised funds, for fiscal decentralisation to be successful it demands a higher capability of local governments in order to deliver efficient and equitable public health services.

## Footnotes

1 The integrated health service post is a centre for children under five, women, and pre- and postnatal healthcare.

2 Children aged 12–23 months should receive one dose of BCG, three doses of polio, three doses of DPT, three doses of hepatitis B and one dose of measles vaccine (Table 1). The DPT, polio, and measles immunisation boosters are recommended after the child reaches 2 years. By their first birthday children should have completed the schedule, and another year is added to cover for possible delays.

## Appendix

**Appendix 1:** Determinants of child's immunisation status: proportion local health expenditure as tertile and quintile

	Model 4	Model 5
Household-level variables		
Residential areas		
(Rural)		
Urban	0.12 (0.04)‡	0.12 (0.04)‡
Birth attendants		
(Non-health professional)		
Health professional	0.40 (0.04)‡	0.40 (0.04)‡
Mothers’ employment status		
(Unemployed)		
Employed	−0.03 (0.03)	−0.02 (0.03)
Mothers’ age		
(≤20 years)		
21–30 years	0.13 (0.06)†	0.13 (0.06)†
>30 years	0.14 (0.07)†	0.13 (0.07)‡
Mothers’ education		
(Primary or less)		
Secondary	0.18 (0.04)‡	0.18 (0.04)‡
Higher	0.30 (0.06)‡	0.30 (0.06)‡
Log households income	0.18 (0.03)‡	0.18 (0.03)‡
District-level variables		
(Local health expenditure as a proportion of total expenditure (lowest tertile))		
Local health expenditure as a proportion of total expenditure (middle tertile)	0.16 (0.12)	
Local health expenditure as a proportion of total expenditure (highest tertile)	0.19 (0.12)	
(Local health expenditure as a proportion of total expenditure (lowest quintile))		
Local health expenditure as a proportion of total expenditure (second quintile)		−0.01 (0.16)
Local health expenditure as a proportion of total expenditure (middle quintile)		−0.00 (0.16)
Local health expenditure as a proportion of total expenditure (fourth quintile)		0.24 (0.15)
Local health expenditure as a proportion of total expenditure (highest quintile)		0.06 (0.16)
Proportion of urban population	0.54 (0.23)†	0.54 (0.23)†
Population density	−0.00 (0.00)‡	−0.00 (0.00)‡
Log GDP per capita	−0.14 (0.07)	−0.13 (0.07)
Between district variance	1.04	1.04
ICC	0.24	0.24
MOR	2.65	2.65

Note: Reported are marginal effects (standard error). Sig.:

* significant at 10% or less

† significant at 5% or less

‡ significant at 1% or less.

**Appendix 2:** Determinants of child's immunisation status: before and after multiple imputation

	Before multiple imputation			After multiple imputation		
		
	Model 1	Model 2	Model 3	Model 1	Model 2	Model 3
Intercept	0.05 (0.02)‡	0.02 (0.01)‡	0.01 (0.01)‡	0.04 (0.02)‡	0.02 (0.01)‡	0.01 (0.01)‡
Household-level variables						
Residential areas						
(Rural)						
Urban	1.12 (0.04)‡	1.13 (0.05)‡	1.13 (0.05)‡	1.13 (0.04)‡	1.12 (0.05)‡	1.12 (0.05)‡
Birth attendants						
(Non-health professional)						
Health professional	1.53 (0.06)‡	1.52 (0.06)‡	1.53 (0.06)‡	1.57 (0.06)‡	1.57 (0.06)‡	1.56 (0.06)‡
Mothers’ employment status						
(Unemployed)						
Employed	0.96 (0.03)	0.97 (0.03)	0.96 (0.03)	0.94 (0.03)	0.94 (0.03)	0.94 (0.03)*
Mothers’ age						
(≤20 years)						
21–30 years	1.13 (0.07)†	1.14 (0.07)†	1.13 (0.07)†	1.14 (0.07)†	1.14 (0.07)†	1.14 (0.07) †
>30 years	1.14 (0.07)†	1.14 (0.07)†	1.14 (0.07)†	1.14 (0.07)†	1.14 (0.07)†	1.14 (0.07)†
Mothers’ education						
(Primary or less)						
Secondary	1.19 (0.04)‡	1.19 (0.04)‡	1.20 (0.04)‡	1.19 (0.04)‡	1.19 (0.04)‡	1.19 (0.04)‡
Higher	1.36 (0.08)‡	1.35 (0.08)‡	1.36 (0.08)‡	1.35 (0.08)‡	1.35 (0.08)‡	1.35 (0.08)‡
Log households income	1.20 (0.04)‡	1.20 (0.04)‡	1.20 (0.04)‡	1.22 (0.04)‡	1.20 (0.04)‡	1.22 (0.04)‡
District-level variables						
Local health expenditure as a proportion of total expenditure (%)		7.68 (12.03)			8.56 (14.40)	
Hospitals/1,000 population			0.52 (1.16)			0.44 (1.02)
Health centres/1,000 population			0.65 (0.18)			0.64 (0.18)
Village health posts/1,000 population			1.71 (0.16)‡			1.88 (0.18)‡
Proportion of urban population		1.68 (0.39)†	2.10 (0.59)‡		1.91 (0.46)‡	2.47 (0.71)‡
Population density		0.99 (0.00)‡	0.99 (0.00)†		0.99 (0.00)‡	0.99 (0.00)†
Log GDP per capita		0.87 (0.06)*	0.88 (0.06)*		0.93 (0.71)	0.92 (0.07)*
Between district variance	1.09	1.05	1.01	1.20	1.20	1.10
ICC	0.25	0.24	0.23	0.27	0.27	0.25
MOR	2.71	2.65	2.61	2.85	2.84	2.72

Note: Reported are marginal effects (standard error). Sig.:

* significant at 10% or less

† significant at 5% or less

‡ significant at 1% or less.

## References

[1] Hawkins L, Srisasalux J, Osornprasop S (2009). Devolution of HealthCenters and Hospital Autonomy in Thailand: A Rapid Assessment. Report from HSRI and World Bank.

[2] Jongudomsuk P, Srisasalux J (2012). A decade of health-care decentralization in Thailand: what lessons can be drawn?. WHO South-East J Public Health.

[3] Merican MI, bin Yon R (2002). Health care reform and changes: the Malaysian experience. Asia Pac J Public Health.

[4] Lieberman SS, Capuno JJ, Minh HV (2005). Decentralizing health: lesson from Indonesia, the Philippines, and Vietnam.

[5] Ministry of Health (1999). Health development plan towards healthy Indonesia 2010.

[6] Bossert TJ, Beauvais JC (2002). Decentralization of health systems in Ghana, Zambia, Uganda and the Philippines: a comparative analysis of decision space. Health Policy Plan.

[7] Khaleghian P (2004). Decentralization and public services: the case of immunization. Soc Sci Med.

[8] Mills A (1994). Decentralization and accountability in the health sector from an international perspective: what are the choices?. Publ Admin Dev.

[9] Gilson L, Mills A (1995). Health sector reforms in sub-Saharan Africa: lessons of the last 10 years. Health Policy.

[10] Bossert T, Chitah MB, Bowser D (2003). Decentralization in Zambia: resource allocation and district performance. Health Policy Plan.

[11] Peckham S, Exworthy M, Greener I, Powell M (2005). Decentralizing health service: more local accountability or just more central control?. Publ Money Manage.

[12] Schneider A (2003). Decentralization: conceptualization and measurement. Stud Comp Int Dev.

[13] Falleti TG (2004). A sequential theory of decentralization and its effects on the intergovernmental balance of power: Latin America cases in comparative perspective.

[14] Shah A (1994). A Reform of intergovernmental fiscal relations in developing and emerging market economies.

[15] Regmi K, Naidoo J, Greer A, Pilkington P (2009). Understanding the effect of decentralisation on health services: the Nepalese experience. J Health Organ Manage.

[16] Asfaw A, Frohberg K, James KS, Jütting J (2007). Fiscal decentralization and infant mortality: empirical evidence from rural India. J Dev Areas.

[17] Robalino DA, Picazo OF, Voetberg A (2001). Does fiscal decentralisation improve health outcomes? Evidence from a cross-country analysis. Policy Research Working Paper No. 2565.

[18] Habibi N, Huang C, Miranda D, Murillo V, Ranis G, Sarkar M (2001). Decentralization in Argentina. Policy Research Working Paper No. 2565.

[19] Uchimura H, Jutting JP (2009). Fiscal decentralization, Chinese style: good for health outcomes?. World Dev.

[20] Cantarero D, Pascual M (2008). Analysing the impact of fiscal decentralization on health outcomes: empirical evidence from Spain. Appl Econ Lett.

[21] Soto VE, Farfan MI, Lorant V (2012). Fiscal decentralisation and infant mortality rate: the Colombian case. Soc Sci Med.

[22] Subramanian SV, Jones K, Kaddour A, Krieger N (2009). The logic in ecological: I. The logic of analysis. Soc Sci Med.

[23] Brenzel L, Claquin P (1994). Immunization programs and their costs. Soc Sci Med.

[24] World Health Organization (2013). Global Vaccine Action Plan 2011–2020.

[25] Feiden R, Firsova S, Gedik G, Huseynov S, Lisitsin Y, Seitkazieva N (1999). Immunization and health sector reform in the Kyrgyz Republic. Report of a WHO-led mission.

[26] Feiden R, Nielsen OF (2001). Immunization and health reform: making reforms work for immunization: a reference guide.

[27] Khaleghian P, Gupta MD (2004). Public management and the essential public health functions. World Bank Policy Research Working Paper 3220.

[28] Thomas MB, Rajesh K (2011). Decentralisation and interventions in health sector: a critical inquiry into the experience of local self governments in Kerala.

[29] Semali IAJ, de Savigny D, Tanner M (2005). Health sector reform and decentralisation in Tanzania: the case of the Expanded Programme on Immunization at district level. J Health Pop Dev Countries.

[30] USAID (2009). Improving child health in Indonesia.

[31] LLP CEPA (2012). Evaluation of GAVI support to civil Society organisations: country evaluation report: Indonesia.

[32] Rokx C, Schieber G, Harimurti P, Tandon A, Somanathan A (2009). Health financing in Indonesia: a reform road map.

[33] Heywood P, Harahap NP (2009). Public funding of health at the district level in Indonesia after decentralization – sources, flows and contradictions. Health Res Policy Syst.

[34] Heywood P, Choi Y (2010). Health system performance at the district level in Indonesia after decentralization. BMC Int Health Hum Rights.

[35] Heywood P, Harahap NP (2009). Human resources for health at the district level in Indonesia: the smoke and mirrors of decentralisation. Hum Resour Health.

[36] Semba RD, de Pee S, Berger SG, Martini E, Ricks MO, Bloem MW (2007). Malnutrition and infectious disease morbidity among children missed by the childhood immunization program in Indonesia. Southeast Asian J Trop Med Public Health.

[37] Ministry of Health (2010). Regulation of Ministry of Health No.482/Menkes/SK/IV/2010 on universal child immunization national movement.

[38] Meloche JP, Vaillancourt F, Yilmaz S (2004). Decentralization or fiscal autonomy what does really matter?. Effects on growth and public sector size in European transition countries.

[39] World Bank (2003). Decentralizing Indonesia: a regional public expenditure review overview report. Report No.26191-IND.

[40] Asparouhov T, Muthén B (2010). Multiple imputation with Mplus. Statmodels.

[41] Larsen K, Merlo J (2005). Appropriate assessment of neighbourhood effects on individual health: integrating random and fixed effects in multilevel logistic regression. AmJ Epidemiol.

[42] Merlo J, Chaix B, Ohlsson H, Beckman A, Johnell K, Hjerpe P (2006). A brief conceptual tutorial of multilevel analysis in social epidemiology: using measures of clustering in multilevel logistic regression to investigate contextual phenomena. J Epidemiol Community Health.

[43] Silver C, Azis IJ, Schroeder L (2001). Intergovernmental transfers and decentralisation in Indonesia. Bull Indones Econ Stud.

[44] Sukarna LA, Budiningsih N, Riyarto S (2006). Analysis of health office readiness in allocation health budget in the decentralised era. Jurnal Manajemen Pelayanan Kesehatan.

[45] Kani A, Herawati DMD, Trisnantoro L (2012). Evaluation of planning and budgeting at Pesisir Selatan district health office, West Sumatera. Jurnal Manajemen Pelayanan Kesehatan..

[46] Durachman Sunartono, Hendrartini J (2007). Process of performance-based fund planning at the health office. Jurnal Manajemen Pelayanan Kesehatan.

[47] Harmana T, Adisasmito WB (2006). Factors associated with local health financing from local government budget of Pontianak district 2006. Jurnal Manajemen Pelayanan Kesehatan.

[48] World Bank (2006). Making services work for the poor: nine case studies from Indonesia.

[49] Bhuiya A, Bhuiya I, Chowdhury M (1995). Factors affecting acceptance of immunization among children in rural Bangladesh. Health Policy Plan.

[50] Abdullah A, Stoelwinder J (2007). Decentralization and health resource allocation: a case study at the district level in Indonesia. World Health Popul.

[51] World Bank (2008). Investing in Indonesia's health: challenges and opportunities for future public spending.

# Sub article: Obesogenic television food advertising to children in Malaysia: sociocultural variations

**DOI:** 10.3402/gha.v7.25169

**Published:** 2014-08-19

**Authors:** See H. Ng, Bridget Kelly, Chee H. Se, Karuthan Chinna, Mohd Jamil Sameeha, Shanthi Krishnasamy, Ismail MN, Tilakavati Karupaiah

**Affiliations:** 1 Dietetics Program, School of Healthcare Sciences, Faculty of Health Sciences, National University of Malaysia, Kuala Lumpur, Malaysia; 2 Early Start Research Institute, Faculty of Social Sciences, University of Wollongong, Wollongong, Australia; 3 Epidemiology and Biostatistics Unit, Department of Social and Preventive Medicine, Faculty of Medicine, University of Malaya, Kuala Lumpur, Malaysia; 4 Nutrition Program, School of Healthcare Sciences, Faculty of Health Sciences, National University of Malaysia, Kuala Lumpur, Malaysia; 5 Nutrition & Dietetics Department, Faculty of Health Sciences, UiTM, Puncak Alam, Malaysia

**Keywords:** content analysis, food marketing, television, sugar-sweetened drink, obesogenic environment

## Abstract

**Background:**

Food advertising on television (TV) is well known to influence children's purchasing requests and models negative food habits in Western countries. Advertising of unhealthy foods is a contributor to the obesogenic environment that is a key driver of rising rates of childhood obesity. Children in developing countries are more at risk of being targeted by such advertising, as there is a huge potential for market growth of unhealthy foods concomitant with poor regulatory infrastructure. Further, in developing countries with multi-ethnic societies, information is scarce on the nature of TV advertising targeting children.

**Objectives:**

To measure exposure and power of TV food marketing to children on popular multi-ethnic TV stations in Malaysia.

**Design:**

Ethnic-specific popular TV channels were identified using industry data. TV transmissions were recorded for each channel from November 2012 to August 2013 (16 hr/day) for randomly selected weekdays and weekend days during normal days and repeated during school holidays (*n=*88 days). Coded food/beverage advertisements were grouped into core (healthy), non-core (non-healthy), or miscellaneous (unclassified) food categories. Peak viewing time (PVT) and persuasive marketing techniques were identified.

**Results:**

Non-core foods were predominant in TV food advertising, and rates were greater during school holidays compared to normal days (3.51 *vs* 1.93 food ads/hr/channel, *p<*0.001). During normal days’ PVT, the ratio of non-core to core food advertising was higher (3.25 food ads/hr/channel), and this more than trebled during school holidays to 10.25 food ads/hr/channel. Popular channels for Indian children had the lowest rate of food advertising relative to other ethnic groups. However, sugary drinks remained a popular non-core product advertised across all broadcast periods and channels. Notably, promotional characters doubled for non-core foods during school holidays compared to normal days (1.91 *vs* 0.93 food ads/hr/channel, *p<*0.001).

**Conclusions:**

This study highlights non-core food advertising, and predominantly sugary drinks are commonly screened on Malaysian TV channels. The majority of these sugary drinks were advertised by multinational companies, and this observation warrants regulatory attention.

Food marketing is an important environmental and contextual factor influencing eating behaviours and is a worldwide public health concern (1). Within marketing, food promotion is a form of communication designed to increase the recognition, appeal and/or consumption of specific food products (2). In this communication environment, television (TV) is a major source of children's exposure to food advertisements (1, 3). TV advertising has long been recognised as an effective medium to reach out to children by food industry and is a primary promotional channel for food marketers (4, 5). Exposure and the power of marketing are two important elements to assess the impact of food marketing on children, as emphasised by the World Health Organization (WHO) (1). ‘Exposure’ is defined as the number of times a viewer is exposed to a message, whereas ‘power’ is defined as a food advertiser's technique to target young and impressionable consumers through the use of promotional characters and premium offers (1, 6). Although content analyses of TV food marketing in developed countries have provided information on the use of the elements of exposure and power in advertisements targeting children (7), such data in developing countries are still scarce.

Gorn and Goldberg (8) were the first to experimentally demonstrate that daily exposure to televised candy advertisements could influence children choosing candy over fruits. The United States National Health Examination Survey indicated that for both young and teenage children, the amount of time spent watching TV was linked to prevalence of obesity (9). Subsequently, systematic reviews have provided modest evidence showing food marketing generates positive beliefs, affects nutrition knowledge, and influences children's food preferences and food consumption patterns, as well as strong evidence that marketing enhances purchase requests to parents (4, 10– 12) . It is proposed that the impact of food promotion on children in developing countries may be greater compared to those in developed countries (13). Such children may be less familiar with advertising and less practiced in navigating commercial messages. The growing middle classes in emerging market economies such as China, India, and many Southeast Asian countries provide unparalleled growth opportunities for global multinational food companies who need to generate new growth after saturation in developed markets (14).

Indeed, it is noted that the world's food system is not a competitive marketplace of small or local producers but driven by multinational food companies (15). It has been documented that in developed countries, marketing strategies employed by multinational food companies target young people to become lifelong consumers and influence household purchases (4, 16). The excessive consumption of often energy-dense ultra-processed foods is blamed for the rising obesity epidemics and incidence of non-communicable diseases (NCDs) in Western nations (17). Of concern is that such marketing strategies, when transplanted to developing countries, would also ultimately result in these health issues if expansion of trade, foreign direct investments, and transnational food corporations proliferate in emerging economies (18). Yet, public health professionals have responded slowly to such nutritional threats in developed countries and even slower in developing countries (15). In a transitional society such as Malaysia which has witnessed economic expansion in the last three decades, overall childhood obesity prevalence in 2006 was reported to be 19.9% in Malaysia, but prevalence patterns by ethnicity were indicated to be 26.6% in Chinese, 26.1% in Indian, and 18.9% in Malay communities (19).

The obesogenic environment in Malaysia is poorly defined. Multinational food companies’ signatories to the International Food and Beverage Alliance (IFBA) have a presence in Malaysia (20). But the specific regulatory criteria promoted by IFBA appear to be permissive in the type of foods suitable for advertising to children (*authors’ opinion)*. A preliminary study in Malaysia conducted in 2006–2007 highlighted that a large proportion of TV advertising (56%) promoted foods high in fat, refined sugars, and salt (21). Concurrent to the time period of this study, Malaysian government guidelines restricted advertising and sponsorship by fast-food companies during children's TV programs (22). In this study, the majority of food advertisements broadcasted on local TV channels were snack foods, dairy products, confectionary, biscuits, and fast food. The limitation of this study was advertising data were provided by participating TV stations rather than adapting live telecast recordings as an independent approach. The method for data analysis changed with a recent study employing live recording in Singapore (5) and evaluating persuasive marketing techniques used by the food industry in Australia (6). Given the evolution of assessments over time in TV food marketing, a need is suggested to adopt new methodologies to effectively evaluate the local TV food marketing scenario.

This study aimed to measure exposure and power of TV food marketing to children in Malaysia, which is a multi-ethnic, developing country. We expect that the outcomes from this study will contribute to existing evidence on the obesogenic environment in three ways: 1) it will explore variations in advertising patterns with seasonal variation (normal days *vs* school holidays) which is recognised as an evidence gap (21, 23) and peak versus non-peak viewing time; 2) it will explore differences in marketing techniques used to target different cultural groups as highlighted by previous researchers (5, 24); and 3) it will explore the use of persuasive techniques in food advertising.

## Methods

### TV channel identification

Popular channels were identified based on ethnic-specific viewership data generated by Nielsen's Television Audience Measurement (TAM) (25). The channels deemed popular were determined through TV viewer rating. Ratings for a 1-week period (9–15 October 2011) identified the most popular channels for children aged 4–14 years based on three major ethnic groups – Malay, Chinese, and Indian. Viewership share (%) of channels was defined as the proportion of individuals’ viewership per channel compared to the total viewership for all channels for same time period (25). Based on these criteria for popular viewership, selected channels were free-to-air (FTA) and satellite TV (Pay-TV) channels that had a household penetration of more than 50%. Selected Pay-TV channels were Astro Cartoon Network (CTWK), Astro Hua Hee Dai (HHD), Astro Wah Lai Toi (WLT), Astro SUN TV (Sun TV), Astro Adithya and Astro Vellithirai (VT). FTA channels excluded were Al-Hijrah and TV1. The majority of the selected channels were not exclusive to children viewership except for the CTWK channel. However, as per TAM data, these channels were still drawing the largest child audience (data not shown). Overall 103 TV channels were assessed by Nielsen's TAM ratings to identify the ethnic-specific popular channels to be used in the data sampling.

### Data sampling

The TV sampling method has been described elsewhere (26, 27). Transmissions from live TV channels were recorded onto hard discs. In brief, TV transmissions were recorded using a personal video recorder (PVR, Kworld Analog TV Card II, Taipei, Taiwan) and software (Windows Media Centre) between 06:00 and 22:00 hours daily (16 hr/day) on randomly selected days falling between November 2012 to August 2013. For each channel, transmissions were recorded for two weekdays and two weekend days in a week during normal days and this cycle was repeated during the school holiday seasons (*n=*88 days). Normal days were defined as schooling days in Malaysia which excluded public holidays or large sporting competitions. This 10-month recording period excluded the Muslim month of *Ramadhan* and *Syawal* which otherwise has been noted to cause variation in advertising exposure (21). The Hindu festive season of *Diwali* also falls outside of the recording period. However, *Christmas* and *Chinese New Year* did fall within the stated 10-month recording period. In Malaysia, *Christmas* is only a single public holiday, and there was no change to advertising patterns observed. *Chinese New Year* has a greater impact on TV advertising patterns, particularly for the popular Chinese TV channels related to a significantly large Chinese population in Malaysia. Therefore, recording transmission data was stopped 2 weeks before Chinese New Year.

### Data coding

Recorded TV transmissions were visually screened for advertising content to identify advertisements which were subsequently coded as per protocol outline in Fig. 1. This protocol is based on criteria described elsewhere (26). Each advertisement was coded for channel identity, date, program details, time slot at which a particular advertisement was broadcasted, and the nature of the product advertised (e.g. retail food and drink, channel promotion, education). All advertisements for retail food/beverage products, supermarkets, and restaurants were further coded into 36 food codes (Table 1) that were each assigned to one of three food categories (core, non-core, and miscellaneous foods). Core foods are nutrient dense and low in discretionary energy and can be recommended to be consumed daily, while non-core foods are high in undesirable nutrients such as high fat, refined sugars, and salt (26). Modifications were made related to food products classification relevant to the Malaysian food supply. For example, sweetened or flavoured milk would be classified as non-core food, whereas non-sweetened milk beverages would be considered as core food. If more than one food product was advertised, the first shown product or the most dominant food product was coded. All food and beverage advertisements were further evaluated for the use of persuasive techniques, including: 1) promotional characters (e.g. cartoons, celebrity endorsers) and 2) premium offers (e.g. giveaways, competitions, contests, vouchers, and rebates).

**Fig. 1:** Process algorithm of data coding. Ad: advertisement. *Code of product, e.g. retail food and drink, channel promotion, education etc. ^†^Protocol with 36 food codes developed based on previous international methodology for TV food advertising (26).

**Table 1:** Seasonal variation in 11 popular TV channels targeting children

		Rate of food advertising (food ads/hr/channel)		
		
	Overall mean (food ads/hr/channel)	Normal days^a^	School holidays	*p*
Non-core foods^b^	2.73	1.93	3.53	<0.001
Sugar-sweetened drinks	0.80	0.43	1.16	
Sweet breads/cakes/muffins/buns/biscuits, glutinous rice balls/cakes/pudding, high-fat savoury biscuits, pies, pastries	0.29	0.29	0.29	
Fast food (not only healthier options advertised)	0.28	0.26	0.29	
Savoury snack foods (added salt or fat) – chips, dried spicy peas, fruit chips, savoury crisps, extruded snacks, popcorn (exclude plain), salted or coated nuts, other fried snacks	0.20	0.13	0.27	
Chocolate and candy	0.17	0.15	0.19	
Flavoured/fried instant rice and noodle products	0.16	0.13	0.19	
Ice cream, iced confection, and desserts	0.15	0.10	0.20	
Flavoured or dairy products with added sugar and alternatives	0.13	0.07	0.19	
High-sugar and/or low-fibre breakfast cereals (>20 g sugars/100 g or <5 g dietary fibre/100 g)	0.13	0.07	0.18	
Meat and meat alternatives processed/preserved in salt	0.11	0.14	0.08	
High-fat/salt meals – frozen, packaged meals (>6 g saturated fat/serve, >900 mg sodium/serve)	0.09	0.06	0.12	
Sweet snack foods – jelly, sugar-coated dried fruits or nuts, nut/seed based bars and slices, sweet rice bars, and tinned fruit in syrup	0.09	0.05	0.13	
Fruit juice/drinks (<98% fruit)	0.08	0.03	0.13	
Other high-fat/salt products – high-fat savoury sauces (>10 g fat/100), soups (>2 g fat/100 g; all dehydrated)	0.07	0.04	0.10	
Alcohol	0.00	0.00	0.00	
Core foods^c^	0.42	0.45	0.39	0.073
Plain milks and yoghurts, cheese, and alternatives	0.13	0.16	0.10	
Breads, rice, and rice products without added fat, sugar, or salt	0.08	0.09	0.07	
Low-sugar, high-fibre breakfast cereals (<20 g sugar/100 g and >5 g fibre/100 g)	0.06	0.07	0.06	
Healthy snacks – <600 kJ/serve, <3 g saturated fat/serve, and <200 mg sodium/serve	0.06	0.05	0.06	
Oils high in mono- or polyunsaturated fats, and low-fat sauces (<10 g fat/100 g)	0.04	0.05	0.03	
Fruits and fruit products without added fats, sugars, or salt	0.02	0.02	0.03	
Meat and meat alternatives	0.01	0.01	0.02	
Water	0.01	0.00	0.02	
Vegetables and vegetable products without added fats, sugars, or salt	0.00	0.00	0.00	
Low-fat/salt meals: meals (≤6 g saturated fat/serve, ≤900 mg sodium/serve), soups (<2 g fat/100 g, exclude dehydrated), sandwiches, mixed salads	0.00	0.00	0.00	
Baby foods (exclude milk formulae)	0.00	0.00	0.00	
Miscellaneous foods/food-related^d^	0.75	0.74	0.76	0.396
Vitamin/mineral or other dietary supplements, and sugar-free chewing gum	0.28	0.27	0.30	
Recipe additions (including soup cubes, oils, dried herbs, and seasonings)	0.16	0.19	0.14	
Baby and toddler milk formulae	0.16	0.11	0.21	
Tea and coffee	0.07	0.08	0.07	
Fast-food restaurant (no foods or beverages advertised)	0.05	0.05	0.04	
Supermarkets (non-core foods advertised)	0.02	0.04	0.00	
Supermarkets (no foods or beverages advertised)	0.00	0.00	0.00	
Fast food (only healthier options advertised)	0.00	0.00	0.00	
Local restaurant	0.00	0.00	0.00	
Supermarkets (only core and healthy foods advertised)	0.00	0.00	0.00	
Ratio of non-core:core	6.54	4.32	9.11	

a Normal days: schooling days exclude national holidays, large sporting competitions, special event and public holiday in Malaysia.

b Food that is relatively high in undesirable nutrients such as high fat, refined sugars, and salt.

c Food that is recommended to be consumed daily to meet nutrient requirements.

d Food that is added to flavour meals (e.g. recipe additions); supplements; milk formula for baby and toddlers; tea and coffee (plain); fast food (with no non-core foods); or local restaurant and supermarkets.

Advertisements were coded by three researchers. To ensure consistency in data coding, an inter-coder reliability test was carried out between researchers based on 1) the number of food advertisements recorded and 2) the food code recorded, using an 1-hour random identical sample of TV data (28). The inter-coder reliability was 100% for both the number of food advertisements recorded, and food coding too.

### Data interpretation

Coded datasets were cleaned and food codes were validated by three professionals (SCH, MJS, KS) with nutrition and dietetic knowledge. Differences in professional opinion were analysed and resolved by an expert panel (BK, TK). Children's peak viewing time (PVT) was defined as the period of the day when ≥25% of the maximum children's audience were likely to be watching TV (26) as defined by Nielsen TAM data for weekday (from 19.00 to 22.00 hours) and weekend (from 15.00 to 16.00 hours and 19.00 to 22.00 hours) periods (29). Viewing time outside the defined PVT was non-peak viewing time (NPVT). Aggregation of each sample was performed to determine the count of advertisements in an hour as described by previous research (30). The average number of food advertisements per hour (rate) was calculated for each food category (core, non-core, and miscellaneous). Rates of core and non-core food advertising were assessed for normal days versus school holidays, for peak versus non-peak viewing times, and also to observe if usage of persuasive techniques in food marketing influenced these rates.

### Statistical analysis

As the rates of food advertisements over time did not fulfil normality assumptions, non-parametric analyses (Mann-Whitney U test) was used to examine seasonal differences between rates of core and non-core food advertising. Kruskal-Wallis test was applied to detect differences between ethnic channels for both normal days and school days. Post hoc analysis for pairwise comparisons between channels was carried out using Dunn Test with Bonferroni correction. A *p-*value threshold of 0.05 was used to determine statistical significance for all data analysis. The statistical analysis was conducted using IBM Statistical Package for Social Sciences, version 19.0 (IBM SPSS Statistics Inc., Chicago, IL).

Figure 2 summarises the flow process of content analysis carried out for this study.

**Fig. 2:** Flow chart of content analysis. For ethnic-specific popular channels, three popular channels were for Malay whilst five popular channels were Chinese and four popular channels were Indian. *However, one TV channel was common to both Malay and Indian viewership. Hence, overall analysis was carried out based on only 11 channels. ^‡^Proportion of individuals’ viewership per channel compared to the total viewership for all channels for same time period. ^†^Protocol developed based on previous international methodology for TV food advertising (26).

## Results

From 103 TV channels identified through Nielsen data, 11 popular channels were related to ethnicity. Malay popular channels were TV2, TV3, and TV9 whilst NTV7, 8TV, HHD, WLT, and CTWK were Chinese and Sun TV, VT, and Adithya were Indian popular channels. One channel (TV2) was common to both Malay and Indian ethnic groups. A mean rate of 3.90 food ads/hr/channel across the 11 children's popular channels in Malaysia was established. Of the 1,408 hours of TV broadcasting that were analysed, a total of 32,194 advertisements were identified of which 5,494 were for food (17.1%).

### Food advertising patterns: normal days versus school holidays

Table 1 provides distribution data for rates of food advertising as differentiated between normal days and school holidays for non-core, core, and miscellaneous food categories. The greatest frequency of food advertising rates associated with non-core foods, irrespective of normal days or school holidays. The rate of non-core food advertising was significantly higher on school holidays compared to normal days (3.53 *vs* 1.93 food ads/hr/channel; *U=*205,492; *p<*0.001) whilst the advertising rate was not significantly different (*p>*0.05) between these days for core or miscellaneous foods. Differences in the rates of non-core food advertising over these periods were attributed to sugar-sweetened drinks, for which the rate of advertising trebled during school holidays (0.43 *vs* 1.16 food ads/hr/channel, *p<*0.001). Similar patterns of exposure for sugar containing snacks like sweet breads, cakes, biscuits (both 0.29 food ads/hr/channel, *p>*0.05), fast foods (0.26 *vs* 0.29 food ads/hr/channel, *p>*0.05), chocolate and candy (0.15 *vs* 0.19 food ads/hr/channel, *p>*0.05) and savoury snack foods (0.13 *vs* 0.27 food ads/hr/channel, *p<*0.01) were detected for both normal days and school holidays. Notably, alcohol was the only non-core product found not advertised at any time, and this is probably attributed to religious restriction in a Muslim dominant country. The mean rates of advertising for core food categories (0.42 food ads/hr/channel) and miscellaneous foods (0.75 food ads/hr/channel) were lower compared to the non-core food rate (2.73 food ads/hr/channel). For every core food advertisement shown, there were nearly four non-core food advertisements shown during normal days, and this figure increased to nine during school holidays. As there was a significant difference in advertising pattern by seasonal variation, the following analyses were conducted separately for normal days and school holidays.

### TV food advertising during children's peak versus non-peak viewing times

Rates of food advertising were consistently higher during children's PVT across all food categories (Table 2). The intensity of non-core food advertising was highest during children's PVT for both normal days (2.62 *vs* 1.74 food ads/hr/channel; *U=*34,504; *p<*0.001) and school holidays (4.53 *vs* 3.26 food ads/hr/channel; *U*=33,276; *p<*0.001). The ratio of non-core:core food advertising during children's PVT was 3.25 during normal days and 10.25 during school holidays. Additionally, there were consistently greater non-core food and low-core food exposures observed in both children's PVT and NPVT during school holidays, resulting in higher non-core to core ratios during these periods (10.25 *vs* 8.74 food ads/hr/channel). These patterns were also reflected when the channels were analysed based on ethnicity. For Malay channels, the non-core foods intensified during school holidays irrespective of PVT or NPVT periods.

**Table 2:** Rate of food advertising during normal days and school holidays as defined by viewing time and ethnic nature of channels

	Rate of food advertising (food ads/hr/channel)					
	
	Normal days			School holidays		
	
	PVT	NPVT	*p*	PVT	NPVT	*p*
Overall popular channels (*n=*11)						
Non-core foods	2.62	1.74	<0.001	4.53	3.26	<0.001
Core foods	0.81	0.35	<0.001	0.44	0.37	0.114
Miscellaneous foods	1.47	0.54	<0.001	1.28	0.61	<0.001
Ratio of non-core:core	3.25	5.01		10.25	8.74	
Malay popular channels (*n=*3)^a^						
Non-core foods	3.12	2.03	0.013	7.36	5.84	0.052
Core foods	0.83	0.30	0.001	0.48	0.48	0.897
Miscellaneous foods	0.98	0.66	0.155	1.07	0.57	0.002
Ratio of non-core:core	3.74	6.78		15.45	12.17	
Chinese popular channels (*n=*5)						
Non-core foods	3.71	2.58	0.005	5.03	3.61	0.003
Core foods	1.03	0.52	<0.001	0.67	0.53	0.080
Miscellaneous foods	2.03	0.66	<0.001	1.91	0.82	<0.001
Ratio of non-core:core	3.61	4.99		7.49	6.83	
Indian popular channels (*n=*4)^a^						
Non-core foods	0.75	0.42	0.044	2.34	1.28	<0.001
Core foods	0.39	0.12	<0.001	0.05	0.04	0.527
Miscellaneous foods	0.80	0.19	0.001	0.41	0.27	0.032
Ratio of non-core:core	1.91	3.65		43.64	36.57	

PVT: peak viewing time of children; NPVT: non-peak viewing time of children.

For ethnic-specific popular channels, three popular channels were for Malay whilst five popular channels were Chinese, and four popular channels were Indian.

a However, one TV channel was common to both Malay and Indian viewership. Hence, overall analysis was carried out based on only 11 channels.

### Food advertising exposure by ethnicity

Generally, Indian channels had the lowest rate of food advertising relative to Malay and Chinese channels as indicated in Table 3. During normal days, Chinese and Malay channels broadcasted similar rates for non-core food advertising (2.83 and 2.27 food ads/hr/channel, respectively) but Indian channels had a significantly lower rate (0.49 food ads/hr/channel; *p<*0.001). In contrast, exposure to non-core food advertising on Malay channels increased almost three times to 6.17 food ads/hr/channel during school holidays and was significantly higher (*p<*0.001) compared to Chinese (3.92 food ads/hr/channel) and Indian (1.51 food ads/hr/channel) channels. Additionally, sugar-sweetened drinks remained as the most commonly advertised food product in the non-core food category across all ethnics’ popular channels (data not shown). Patterns of exposure to core and miscellaneous food advertisements during normal days and school holidays did not differ and remained relatively low for all ethnic groups’ popular channels compared to non-core food advertising rates.

**Table 3:** Food advertising exposure as per seasonal variation by TV channel ethnicity

	Rate of food advertising (food ads/hr/channel)							
	
	Normal days				School holidays			
	
	Malay channels (*n=*3)^a^	Chinese channels (*n=*5)	Indian channels (*n=*4)^a^	*p*	Malay channels (*n=*3)^a^	Chinese channels (*n=*5)	Indian channels (*n=*4)^a^	*p*
Non-core foods	2.27^a^	2.83^b^	0.49^a^^b^	<0.001	6.17^a^^b^	3.92^bc^	1.51^a^^bc^	<0.001
Core foods	0.42^a^^b^	0.63^bc^	0.18^a^^bc^	<0.001	0.48^a^	0.56^b^	0.04^a^^b^	<0.001
Miscellaneous foods	0.73^a^	0.96^b^	0.32^a^^b^	<0.001	0.68^a^	1.06^b^	0.30^a^^b^	<0.001

For ethnic-specific popular channels, three popular channels were for Malay whilst five popular channels were Chinese, and four popular channels were Indian. Values in the same row sharing same superscript letters are significantly different. Dunn test: *p<*0.01.

a However, one TV channel was common to both Malay and Indian viewership.

### Persuasive techniques

The most common persuasive technique used in TV food advertising was the use of promotional characters, and this trend was common to all ethnics channels selected. As indicated in Fig. 3, the rate of promotional characters used in food advertising was more relevant to non-core foods than core foods or miscellaneous food products. In contrast, the use of premium offer in food advertising was consistently low across both time periods. However, premium offers were more often associated with non-core foods.

**Fig. 3:** Persuasive techniques as per seasonal variation and TV channel ethnicity. (a) Promotional characters used in food advertisements. (b) Premiums offered in food advertisements. For ethnic-specific popular channels, three popular channels were for Malay whilst five popular channels were Chinese and four popular channels were Indian. One TV channel was common to both Malay and Indian viewership. Hence, overall analysis was carried out based on only 11 channels. Within each figure, values sharing same superscripts are significantly different between normal days and school holidays.

## Discussion

This study identified higher rates of unhealthy food advertising occurred during school holiday periods compared to normal days for children. This is a marketing strategy because children would naturally have more free time to watch TV as suggested by Boyland et al. (31). Food advertising was pervasive throughout school holidays across all ethnic channels and mainly promoted sugar-sweetened drinks. A systematic review and meta-analysis has established a link between sugar-sweetened beverage consumption and increased risk of childhood obesity (32). A study in the United States indicated that every incremental increase of 100 advertisements of sugar-sweetened drinks was associated with a 9.4% increase in children's consumption of soft drinks (33). Thus, our finding on the high rate of sugary drink advertising is a serious concern in Malaysia if this is going to reflect an increased consumption of soft drinks by children.

Consistent with a previous study conducted in Malaysia in 2006 (21), the current findings indicated non-core foods were the most advertised food products. However, we detected a change in the type of food products most frequently advertised. Based on content analysis of TV food advertising data, Karupaiah et al. (21) identified unhealthy snacks as the most dominant advertised food products in contrast to the sugar-sweetened drinks reported in our study. This increment in advertising of sugar-sweetened drinks is also reported in Spain, India, and other Asia Pacific countries with this product being the most frequently advertised beverage on TV (29, 34, 35). From our observation of TV media, the increment in advertising for sugary drinks since 2006 (21) mostly originates from multinational companies, such as Coca-cola, Nestle, and Pepsico, as opposed to local brands.

Current Malaysian government guidelines restricting advertising and sponsorship by fast-food companies during children's TV programs apply during children's program targeting 4–9 year olds and when children's TV viewership exceeds 4% (22). Despite these restrictions, fast foods were one of the three most popular advertised non-core foods. We also noted that during children's PVT, the rate of fast-food advertisements was higher compared to NPVT (0.41 *vs* 0.24 food ads/hr/channel). This indicates current government regulations are limited in their ability to protect children from fast-food advertising on TV. The impact of this guideline is also limited by the lack of provisions related to other unhealthy food and beverages, particularly sugar-sweetened drinks. As demonstrated by viewership audience data in our study, the large majority of children watch TV outside of the targeted children's programs for which guidelines apply, with PVT occurring during the evening periods.

An alternative approach to monitoring and evaluating food advertising is by using children audience composition data as a standardised method to capture significant proportions of children exposed to marketing campaigns (1). A recent study suggested PVT reflected actual exposure of children to non-core food advertising (23). Based on our findings, the exposure of non-core foods was consistently higher during PVT compared to NPVT for children. The IFBA is now committed only to advertising products that meet specific nutritional criteria based on accepted national and international evidence and/or applicable national and international dietary guidelines in 2008 (20). However, our findings contradict a report from this alliance which found TV food and beverages advertising compliance was 99.2% (20). A reason for this discrepancy is that the IFBA code of practice only applies during TV viewing times when children make up at least 50% of the audience, which does not often occur in the real-world scenario (7). Further, the IFBA code is more permissive of foods that are actually inappropriate to be advertised to children, and is therefore relaxed about advertising of these foods. For instance, cereals containing sugar up to 35 g/100 g are deemed acceptable to be marketed to children, whereas expert opinion for healthy eating is that permitted foods should not exceed 20 g sugar/100 g (7).

We noted distinct differences in food advertising rates on Indian channels relative to Malay and Chinese channels. Most of the popular channels for Indian children were filtered satellite channels originating from India. Such foreign-origin advertisements are modified/removed as these are not relevant to the local market in Malaysia. It is also possible that these channels are less targeted by food companies, given the relatively low population numbers of this ethnic group in Malaysia compared to other ethnic groups (Malays or Chinese) as well as their lower household expenditures for these products (36). Conversely, non-core food advertising was found to be prevalent in Malay and Chinese channels and remarkably higher in Malay channels during school holidays. The non-core food advertising exposure pattern does not align with the reported patterns of childhood obesity by ethnicity in Malaysia (Chinese 26.6%>Indian 26.1%>Malay 18.9%) (19). However, obesity is well known to be a complex issue with multiple determinants (37). By using a mathematical simulation model, it was projected that TV food advertising contributed to 15–40% of obesity prevalence in the United States, and an absence of unhealthy food advertising on TV therefore could yield a reverse shift of proportion from overweight children to normal weight (38).

Promotional characters were commonly used as persuasive marketing technique for non-core foods in Malaysia, and this marketing practice was prevalent during school holidays compared to normal days. This is consistent with previous research which highlight that persuasive marketing techniques were mainly used in non-core food relative to core food advertisements (6, 39). On further examination of the non-core food category, we found sugar-sweetened drinks, breakfast cereals, extruded snacks, ice cream, and instant noodles were more likely to be using the persuasive techniques (data not shown). Promotional characters included branded cartoons or celebrities or famous actors who were company spokespersons for the non-core food products. The advertising impact of this technique is well documented to show associations with brand recognition, positive attitudes towards food products, and even brand loyalty at an earlier age (6, 40). Further, enhanced consumption of high-carbohydrate and high-fat foods by overweight and obese children is associated with the use of promotional characters during food advertising (41). Repetition of promotional characters from food advertising could transfer the positive effects related to characters (42) and even more, credibility of celebrities in their own field would be mistaken and further extended to the product they are endorsing (43).

Our results are constrained by the fact that this is a cross-sectional study. However, the selection of TV channels is valid to represent TV exposure patterns generally as the data includes normal days, weekdays, and weekend days, and at the same time provides data for all ethnic-specific popular TV channels. A major strength of this study is to provide a wider scope of understanding content analysis of food advertising on Malaysian TV channels by including seasonal variation as a factor and an improved model for classifying food codes previously standardised by researchers in a multicentre international study (35). It is important to note that in this study, the selection of TV channels was specific to ethnicity and ranked by children's preference as determined by Nielsen's TAM data. Further, persuasive techniques of marketing used in TV food advertising were assessed for the first time in Malaysia.

Based on our findings it is apparent that stringent regulation of TV food advertising is critical during PVT, which relates to children's viewership. The prior implementation of a content code that bans alcohol advertising in Muslim-majority Malaysia, which was successfully reflected in a zero rate detection of alcohol advertisements on TV, indicates that advertising content restrictions are possible. In contrast, our results also showed that current regulations in Malaysia are not able to protect children entirely from high rates of non-core food advertising on TV. Lastly, as highlighted by public health professionals, a standardised set of definitions for classifying TV food products according to health values, and specifying children's peak viewing period will enable children to be better protected, either directly or indirectly from exploitation by TV food marketing.

## Conclusions

This study revealed children's high exposure to non-core food advertising on Malaysian TV channels. Non-core food advertisements were shown four times more frequently during normal days than core foods, and the non-core food advertising rate doubled during school holidays. Food advertising exposure varied among channels popular with ethnic groups, suggesting policy to regulate advertising should factor ethnicity in the future. The high rate of advertising for sugary drinks warrants a further regulatory action by government to limit these advertisements.

## References

[1] World Health Organization (2012). A framework for implementing the set of recommendations on the marketing of foods and non-alcoholic beverages to children.

[2] World Health Organization (2010). Global strategy to reduce the harmful use of alcohol.

[3] OFCOM (2004). Childhood obesity – food advertising in context: children's food choices, parents’ understanding and influence, and the role of food promotion. http://stakeholders.ofcom.org.uk/binaries/research/tv-research/report2.pdf.

[4] Cairns G, Angus K, Hastings G, Caraher M (2013). Systematic reviews of the evidence on the nature, extent and effects of food marketing to children. A retrospective summary. Appetite.

[5] Huang L, Mehta K, Wong ML (2011). Television food advertising in Singapore: the nature and extent of children's exposure. Health Promot Int.

[6] Kelly B, Hattersley L, King L, Flood V (2008). Persuasive food marketing to children: use of cartoons and competitions in Australian commercial television advertisements. Health Promot Int.

[7] Galbraith-Emami S, Lobstain T (2013). The impact of initiatives to limit the advertising of food and beverage products to children: a systematic review. Obes Rev.

[8] Gorn GJ, Goldberg ME (1982). Behavioral evidence of the effects of televised food messages on children. J Consum Res.

[9] Dietz WH, Gortmaker SL (1985). Do we fatten our children at the TV set? Television viewing and obesity in children and adolescents. Pediatrics.

[10] World Health Organization (2009). The extent, nature and effects of food promotion to children: a review of the evidence to December 2008.

[11] Keller SK, Schulz PJ (2010). Distorted food pyramid in kids programmes: a content analysis of television advertising watched in Switzerland. Eur J Public Health.

[12] Livingstone S, Helsper E (2004). Advertising foods to children: understanding promotion in the context of children's daily lives. A review of the literature for the Market Research Department of the Office of Communications (OFCOM).

[13] World Health Organization (2006). The extent, nature and effects of food promotion to children: a review of the evidence.

[14] Hawkes C (2002). Marketing activities of global soft drink and fast food companies in emerging markets: a review.

[15] Stuckler D, Nestle M (2012). Big food, food systems, and global health. PLoS Med.

[16] Consumers International (2008). The junk food trap: marketing unhealthy food to children in Asia Pacific 2008.

[17] Moodie R, Stuckler D, Monteiro C, Sheron N, Neal B, Thamarangsi T (2013). Profits and pandemics: prevention of harmful effects of tobacco, alcohol, and ultra-processed food and drink industries. Lancet.

[18] Kearney J (2010). Food consumption trends and drivers. Philos Trans R Soc B Biol Sci.

[19] Naidu BM, Mahmud SZ, Ambak R, Mohd Sallehuddin S, Abdul Mutalip H, Saari R (2013). Overweight among primary school-age children in Malaysia. Asia Pac J Clin Nutr.

[20] Accenture (2012). 2012 Compliance monitoring report for the International Food & Beverage Alliance on global advertising on television.

[21] Karupaiah T, Chinna K, Loi HM, Lim SM, Noor MI (2008). What's on Malaysian television? – a survey on food advertising targeting children. Asia Pac J Clin Nutr.

[22] World Obesity (2010). Malaysia policy overview. World Obesity Federation.

[23] Smithers LG, Lynch JW, Merlin T (2014). Industry self-regulation and TV advertising of foods to Australian children. J Paediatr Child Health.

[24] Lobstein T (2013). Research needs on food marketing to children. Report of the StanMark project. Appetite.

[25] Nielsen Television Audience Measurement (2011). TV viewership report from 9.10.2011 till 15.10.2011.

[26] Kelly B, Halford JC, Boyland EJ, Chapman K, Bautista-Castano I, Berg C (2010). Television food advertising to children: a global perspective. Am J Public Health.

[27] Kelly B, King L, Baur L, Rayner M, Lobstein T, Monteiro C (2013). Monitoring food and non-alcoholic beverage promotions to children. Obes Rev.

[28] Zuppa J, Morton H, Mehta K (2003). Television food advertising: counterproductive to children's health? A content analysis using the Australian Guide to Healthy Eating. Nutr Diet.

[29] Kelly B, Hebden L, King L, Yang X, Yang Y, Gengsheng H (2014). Children's exposure to food advertising on free-to-air television: an Asia-Pacific perspective. Health Promot Int.

[30] King L, Hebden L, Grunseit A, Kelly B, Chapman K (2012). Building the case for independent monitoring of food advertising on Australian television. Public Health Nutr.

[31] Boyland EJ, Harrold JA, Kirkham TC, Halford JCG (2011). The extent of food advertising to children on UK television in 2008. Int J Pediatr Obes.

[32] Malik VS, Pan A, Willett WC, Hu FB (2013). Sugar-sweetened beverages and weight gain in children and adults: a systematic review and meta-analysis. Am J Clin Nutr.

[33] Andreyeva T, Kelly IR, Harris JL (2011). Exposure to food advertising on television: associations with children's fast food and soft drink consumption and obesity. Econ Hum Biol.

[34] Romero-Fernández MM, Royo-Bordonada MA, Rodríguez-Artalejo F (2012). Evaluation of food and beverage television advertising during children's viewing time in Spain using UK nutrient profile model. Public Health Nutr.

[35] Ghimire N, Rao A (2013). Comparative evaluation of the influence of television advertisements on children and caries prevalence. Glob Health Action.

[36] Department of Statistics Malaysia (2012). Report on household expenditure survey. http://www.statistics.gov.my/portal/download_household/files/Expenditure/Laporan_PIR_Report_on_HES_2009-10.pdf.

[37] Butland B, Jebb S, Kopelman P, McPherson K, Thomas S, Mardell J (2007). Foresight – tackling obesities: future choices – project report. https://www.gov.uk/government/uploads/system/uploads/attachment_data/file/287937/07-1184x-tackling-obesities-future-choices-report.pdf.

[38] Veerman JL, Van Beeck EF, Barendregt JJ, Mackenbach JP (2009). By how much would limiting TV food advertising reduce childhood obesity?. Eur J Public Health.

[39] Boyland EJ, Harrold JA, Kirkham TC, Halford JCG (2012). Persuasive techniques used in television advertisements to market foods to UK children. Appetite.

[40] Neeley S, Schumann D (2004). Using animated spokes – characters in advertising to children. J Adv.

[41] Boyland EJ, Halford JCG (2013). Television advertising and branding. Effects on eating behaviour and food preferences in children. Appetite.

[42] Mizerski R (1995). The relationship between cartoon trade character recognition and attitude towards product category in young children. J Market.

[43] Kaiser Family Foundation (2007). Food for thought. Television food advertising to children in the United States.

# Sub article: Human resources for health: task shifting to promote basic health service delivery among internally displaced people in ethnic health program service areas in eastern Burma/Myanmar

**DOI:** 10.3402/gha.v7.24937

**Published:** 2014-09-29

**Authors:** Sharon Low, Kyaw Thura Tun, Naw Pue Pue Mhote, Saw Nay Htoo, Cynthia Maung, Saw Win Kyaw, Saw Eh Kalu Shwe Oo, Nicola Suyin Pocock

**Affiliations:** 1 Community Partners International, Mae Sot, Thailand; 2 Burma Medical Association, Mae Sot, Thailand; 3 Health Information System Working Group, Mae Sot, Thailand; 4 Back Pack Health Worker Team (BPHWT), Mae Sot, Thailand; 5 Mae Tao Clinic, Mae Sot, Thailand; 6 Karen Department of Health and Welfare (KDHW), Mae Sot, Thailand; 7 Department of Global Health and Development, London School of Hygiene and Tropical Medicine, London, UK

**Keywords:** health system strengthening, health workforce, task shifting, internally displaced people, Burma/Myanmar

## Abstract

**Background:**

Burma/Myanmar was controlled by a military regime for over 50 years. Many basic social and protection services have been neglected, specifically in the ethnic areas. Development in these areas was led by the ethnic non-state actors to ensure care and the availability of health services for the communities living in the border ethnic-controlled areas. Political changes in Burma/Myanmar have been ongoing since the end of 2010. Given the ethnic diversity of Burma/Myanmar, many challenges in ensuring health service coverage among all ethnic groups lie ahead.

**Methods:**

A case study method was used to document how existing human resources for health (HRH) reach the vulnerable population in the ethnic health organizations’ (EHOs) and community-based organizations’ (CBHOs) service areas, and their related information on training and services delivered. Mixed methods were used. Survey data on HRH, service provision, and training were collected from clinic-in-charges in 110 clinics in 14 Karen/Kayin townships through a rapid-mapping exercise. We also reviewed 7 organizational and policy documents and conducted 10 interviews and discussions with clinic-in-charges.

**Findings:**

Despite the lack of skilled medical professionals, the EHOs and CBHOs have been serving the population along the border through task shifting to less specialized health workers. Clinics and mobile teams work in partnership, focusing on primary care with some aspects of secondary care. The rapid-mapping exercise showed that the aggregate HRH density in Karen/Kayin state is 2.8 per 1,000 population. Every mobile team has 1.8 health workers per 1,000 population, whereas each clinic has between 2.5 and 3.9 health workers per 1,000 population. By reorganizing and training the workforce with a rigorous and up-to-date curriculum, EHOs and CBHOs present a viable solution for improving health service coverage to the underserved population.

**Conclusion:**

Despite the chronic conflict in Burma/Myanmar, this report provides evidence of the substantive system of health care provision and access in the Karen/Kayin State over the past 20 years. It underscores the climate of vulnerability of the EHOs and CBHOs due to lack of regional and international understanding of the political complexities in Burma/Myanmar. As Association of Southeast Asian Nations (ASEAN) integration gathers pace, this case study highlights potential issues relating to migration and health access. The case also documents the challenge of integrating indigenous and/or cross-border health systems, with the ongoing risk of deepening ethnic conflicts in Burma/Myanmar as the peace process is negotiated.

Burma/Myanmar is ranked 149 out of 168 countries on the Human Development Index (HDI). It has been characterized as a fragile state due to its governance record and ongoing conflict in many parts of the country (1). The successive military regimes that held power from 1962 to 2011 were engaged in ongoing conflicts with ethnic minority groups in many parts of the country. During that period, regime policies resulted in forced labor, forced relocation, torture, killings, and deliberate destruction of food supplies (2), largely targeted at ethnic minorities (3). The eastern Burmese border bore the largest burden of internally displaced people (IDPs) (4). During this time, there was no official government effort to provide health care for affected civilians in ethnic-controlled areas of the Karen/Kayin State (Fig. 1) (5).

**Fig. 1:** Location of Karen/Kayin State, eastern Burma/Myanmar.

Despite recent commitments to increase government spending on the health sector, the projections for health spending remains below 0.76% of gross domestic product (GDP) for 2012/2013 (6). Based on the 2009 World Health Organization (WHO) report, external donor support is also low (US$11.20 per capita) compared to regional counterparts like Cambodia and Laos, which are receiving US$22.20 and US$12.90 per capita, respectively (7). As a result, access to basic health services remains very poor in both ethnic-controlled and government-controlled areas of this region (3, 8, 9).

Due to the restricted national and international efforts (10, 11) to aid Burma/Myanmar’s IDPs, ethnic health organizations (EHOs) such as the Karen Department of Health and Welfare (KDHW) and community-based health organizations (CBHOs) such as the Burma Medical Association (BMA) and Back Pack Health Workers Team (BPHWT) were created to fill this gap. These entities play a crucial role in delivering essential health care to the IDPs in locations where, without these organizations, there would be none^1^ .

However, similar to many other challenging contexts, the health workers shortage is one of the main constraints of the Burma/Myanmar health system, as noted by Risso-Gill (2013). Nationally, the level of qualified human resources in Burma/Myanmar is an average of 1.4 doctors, nurses, and midwives (combined) per 1,000 persons (12), which is below the WHO critical shortage threshold of 1.7 health workers per 1,000 population^2^(13). This human resource shortage is even more amplified in the eastern ethnic-controlled border regions due to geographical remoteness, rural poverty, civil conflict, and limited access to education. As a result, *task shifting* was adopted as an innovative strategy to redistribute specialized primary health care (PHC) tasks that are usually administered by doctors and nurses to an active network of community-based medics, maternal health workers (MHWs), and community health workers (CHWs) (14, 15). More information about the tasks of each health worker to delivery PHC at village and village tract levels will be described in the ‘Findings’ section of this article.

The PHC service is operationalized collaboratively between the KDHW, BMA, and BPHWT in different townships and villages. The program routine data revealed that about 50% of the health services in the Karen State were delivered by the BPHWT mobile teams (Box 1). The other 50% were delivered by either KDHW and/or BMA clinics. Generally, the KDHW works in areas that are controlled by the Karen National Union (KNU), whereas the BMA and BPHWT are not tied to any political affiliation^3^ . For KNU areas, there are generally coordination and agreement between the three organizations, even though the target populations could overlap specifically for the coverage areas of BPHWT and KDHW mobile health teams. For the BPHWT teams, they would work only in areas where the community leaders see the need to be part of the BPHWT service coverage. Field staff from these organizations collect population data (Table 1) from their service area on an annual basis.

**Table 1:** Study area of ethnic townships: their names by government official and target population

No	Township	Government official name	Target population
1	Kawkareik	Kawkareik	45,356
2	Bu Tho	Hpapun	20,190
3	Lu Thaw	Hpapun	32,510
4	Na Bu	Myawaddy	34,697
5	Billin	Billin	23,494
6	Kyainseikgyi	Kyainseikgyi	14,440
7	Win Yee	Ye	30,516
8	Hlaing Bwe	Hlaingbwe	13,742
9	Htaw Ta Htoo	Htantabin	1,194
10	Dweh Loe	Hpapun	14,322
11	Kyauk Kyi	Kyaukkyi	6,792
12	K’ser Doh	Myeik	5,601
13	Shwe Kyin	Shwekyin	6,260
14	Ler Muh Lah	Myeik	4,614
Total target population			253,728

***Box 1*. EHOs and CBHOs in Karen/Kayin State**

The *Burma Medical Association* (BMA) was founded in 1991 by a group of health professionals from Burma/Myanmar. The BMA is an independent non-profit organization. Doctors, nurses, and other health professionals representing multiple ethnic groups gathered in Manerplaw, the former headquarters for the Karen National Union (KNU), to establish a forum for promoting health and human rights among displaced people from Burma/Myanmar. For the past 22 years, the BMA has been the leading body for health policy development and capacity building for the provision of quality health care services in ethnic areas of Burma/Myanmar.

The *Back Pack Health Worker Team* (BPHWT) was established in 1998 by Karenni, Mon, and Karen health workers to provide health care to internally displaced people living along the eastern border of Burma/Myanmar who have been affected by many decades of civil war. The BPHWT aims to improve health through the delivery of primary health care (PHC) and public health promotion. They provide medical care, community health education and prevention, maternal and child health care, and water and sanitation programs in the targeted field areas. Integrated through these PHC programs are health information and documentation and capacity-building programs.

In 1991, the KNU established the *Karen Department of Health and Welfare* (KDHW) to provide PHC to all people living in Karen/Kayin State. From 1991 to 1997, the KDHW administered the hospitals and clinics in all seven districts of Karen State, but the State Peace and Development Council (SPDC) offensive of 1997 decimated most of that health care infrastructure. In response, the KDHW organized the first mobile health clinic in 1998. Together with the Committee for Internally Displaced Karen People (CIDKP) and the BPHWT, the KDHW established additional mobile health clinics each year.

This article aims to advocate for the government and donors to acknowledge and build on the gains of the EHOs and CBHOs in health system strengthening, and not to risk alienating the local worker and ethnic communities. Although numerous studies had been conducted concerning health workers in eastern Burma/Myanmar on various topics, such as malaria knowledge (16), medic’s experiences of trauma and mental health (17), health workers’ strategies for addressing security and ensuring access to vulnerable ethnic communities (18), and perspectives from MHWs on delivering community-based care (19), this article is the first known attempt to document how the ethnic system of task shifting during the conflict period has effectively contributed toward the development of a strong ethnic health workforce to satisfy the essential health needs of the population in the EHOs’ and CBHOs’ service areas. It represents an empirical effort to map the health workforce (in their numbers, services, and training content) that is currently operating in Karen State.

## Data and methods

A case study approach was undertaken to document the structure of the health workforce in Karen State, and to compile comparable data related to the HRH, including their level of training and the health services that they delivered. This study is based on information collected through a rapid-mapping exercise that was conducted in 2012 with clinic-in-charges in 110 clinics in 14 Karen townships (Fig. 2). A one-paged survey questionnaire asking for information such as the number of health workers working in the clinics, their training, and the services provided by the clinics was used for the rapid mapping exercise.

**Fig. 2:** Study area of ethnic townships.

Additional information on various levels of training and their outlines was obtained through a desk review of seven main organizational policy and training documents, which reflected the development of the HRH and were easily accessible. Ten informal context interviews were conducted with clinic-in-charges during their biannual meeting in Mae Sot. Interviews were facilitated by three health information system (HIS) staff. The initial draft of this article was forwarded for review and feedback by the five EHO and CBHO leaders who were integrally involved in the HRH development through policy, planning, or training. Two of them provided substantial guidance for the direction of this article.

No formal approval was obtained from an Institutional Review Board (IRB) as none exists in Karen State. However, the mapping exercise were reviewed and approved by individual EHOs and CBHOs as well as district-in-charges prior to data collection. The leaders also gave specific permission to the Health Information Systems Working Group (HISWG)^4^ to analyze the data collected from the rapid mapping. Additionally, there is no sensitive or intrusive information related directly to the population. The mapping was focused on HRH, their training, and the types of services provided to the community.

The issues investigated included five categories of interest: 1) density of the HRH, 2) systematic and rigorous training, 3) PHC service delivery, 4) level of cooperation and systematic approaches in task shifting, and 5) perceptions of task shifting among HRH. These themes were developed on the basis of lessons from the international literature, as well as the areas in which the EHOs and CBHOs are ready to engage in dialogues with the Burmese government, to mainstream HRH training and service delivery.

## Findings

### Density of HRH

Despite the lack of qualified health workers like doctors and nurses, the EHOs and CBHOs have been serving the population at the border through a lower-level cadre of health workers like medics, MHWs, and CHWs. Table 2 presents the assessment data from the rapid-mapping exercise where HRH at the aggregate level in the Karen State suggests no critical shortage of health workers, with a regional average of 2.8 medics, MHW, and CHW (combined) per 1,000 population (Table 2).

**Table 2:** Basic health workers’ densities in target population in Karen/Kayin state (authors’ calculations)

		Health workforce				Density per 1,000 population			
			
	Target population	Medic	MHW	CHW	Combined	Medic	MHW	CHW	Combined
BMA	42,358	25	23	59	107	0.6	0.5	1.4	2.5
KDHW	112,521	108	73	254	435	1.0	0.6	2.3	3.9
BPHWT	98,849	181	–	–	181	1.8	–	–	1.8
Total (14 townships)	253,728	314	96	313	723	1.2	0.4	1.2	2.8

The clinics and mobile teams work together in the community, with the clinics providing secondary care, performing deliveries, and providing specialized care. From a provider’s perspective^5^, it is projected that every mobile team (BPHWT) has roughly 1.8 health workers for a population of about 1,000 people, whereas each clinic (BMA or KDHW) has about 2.5–3.9 health workers for a population of about 1,000 people (Table 2). Although this density per 1,000 population for eastern Burma/Myanmar is higher than the national average, it should be noted that there is more division of labor in task shifting due to the vertical programming pushed out by donors through pooled funding such as the Three Millennium Development Goals (3MDG) (20), as well as due to the geographical remoteness of some of these areas.

### Systematic and rigorous training

Another critical element in task shifting is to ensure that the cadres that will be taking on the tasks are appropriately trained. Each of these health workers has gone through a set of systematic training, including an annual refresher. Biannually, they are also updated on the most current internationally approved case definitions and treatment protocols (21).

The EHOs and CBHOs trained the first cohort of medics in 1979. The curriculum has been revised several times and is now a 10.5-month training program. To date, a total of 27 cohorts have graduated and are working in the communities. The CHW program was first initiated in 1981 and was based on the curriculum set by the WHO. The training consists of an initial 6 months of theory followed by a 3-month practicum. In response to a shortage of skilled birth attendants in the Karen State, the EHOs and CBHOs established an 8-month MHW program, recruiting and training local women to deliver regular to complex care. This is to complement the pool of trained traditional birth attendants (TTBAs) and traditional birth attendants (TBAs) who have undergone 6- and 1-week trainings, respectively, with the EHOs/CBHOs.

International non-governmental organizations (INGOs) like the International Rescue Committee (IRC) and Community Partners International (CPI) work with the EHOs and CBHOs to build capacities in the areas of health workers’ training, HIS, and public health. They also coordinate with other CBHOs and the Mae Tao Clinic (MTC) (22), a community hospital in western Thailand serving the Burmese migrant and internal displaced people, in managing outbreak of diseases and referral of patients, as well as for medical training. There are also a number of ongoing training programs focusing on specific need areas like malaria and maternal and child health (MCH).

### Primary health care service delivery

To establish a framework for a sustainable health system by strengthening the health service delivery approaches in current EHO and CBHO areas, the tasks of medics, MHWs, and CHWs are clearly articulated in the basic package of health services for the Karen State:

Medical care: Involves medics who diagnose and treat the six common illnesses of malaria, acute respiratory infection (ARI), anemia, worm infestation, diarrhea, and dysentery. The medics are equipped with rapid diagnostic tests for malaria, as well as antimalarials, antibiotics for pneumonia and dysentery, and a variety of other essential medicines. They are trained in emergency care for injuries, and they have some referral sources for more complicated diseases.

MCH: MHWs are trained to use clean delivery kits, provide antenatal care (ANC) and postnatal care (PNC) to encourage safe deliveries and healthy infants, provide delivery and postpartum care, as well as distribute family planning (FP) methods. They are also trained in providing emergency obstetric care (EmOC), immunizations, and management of child health.

Community health education and prevention: Besides supporting the medics in diagnosing and treating the six common diseases, CHWs will also coordinate school health promotion activities, train village health workers (VHWs), and hold community health education workshops. Prevention activities include biannual deworming and vitamin A for children, as well as coordinating with village heads regarding the construction and maintenance of water systems and latrines.

### Level of cooperation and systematic approach to task shifting

In terms of organization, as mentioned in this article, the EHOs and CBHOs collaborate closely with each other and share responsibilities in a consensus-seeking manner.

The KDHW accordingly organizes coordination meetings every six month, in conjunction with the regular KDHW program meetings, field workshops, field operational meetings, and village workshops. – KDHW health worker

The clinic-in-charges from 15 field areas organized field meetings every 6 months, which included coordinated activities with ethnic health departments,local community based organizations, school teachers and leaders. – BMA health worker

From the informal interviews and protocols reviewed, it was apparent that EHOs and CBHOs have adopted a systematic approach in task shifting with the local delivery of health services (Fig. 3):

1. Planning and coordination between EHOs and CBHOs based on principles of health equity;
2. Training of medics (10 months), MHWs (8 months), and CHWs (6 months);
3. Support and monitoring once every 6 months with a review of log records and data;
4. Referral to high-level care; as well as
5. Assessment and evaluation of health workers’ performance, field consultation, and population-based surveys.

**Fig. 3:** Systems approach in task shifting by EHOs.

### Perception of task shifting among health workers

Health workers were generally positive about task shifting and the participatory system, which are necessary to succeed in the eastern Burma/Myanmar context and to ensure equity in access to health care with an emphasis on primary care and community self-reliance.

It depends on local people and local organizations. If they say their place is safe, we go there. We build makeshift tents with banana leaves and tarpaulin, and we give medical treatment. We can give medical treatment in some villages where the ethnic armed groups are in control. – BPHWT health worker

The incidence of diarrhea and cholera has decreased. Local community organizations have greater trust in our team … our approach is to go straight to the people and not to wait for the people to come to us. – BMA/BPHWT board member

## Discussion

Since voluntarily transitioning from a strict military regime to a quasi-civil government in the 2010 general election, Burma/Myanmar has undergone major political changes, indicating strong signals of willingness to reengage with the international community. However, decades of underinvestment and neglect of public services have resulted in a fragile and weak health system, which is reflected in poor health outcomes (23, 24). Whereas other parts of Burma/Myanmar may see improvements in development and access to health services as the economic and political framework of the country is reordered, it is likely that IDP communities in the Karen State will continue to be excluded from mainstream health services and may suffer further conflict as a result of the nation’s overall transition (5). A different strategy is needed for eastern Burma/Myanmar. This article outlines the ethnic service delivery model together with the strengths of its HRH, who are accepted by the population in this region.

### Task shifting ensures equitable access – health for all

The availability and accessibility of health facilities, goods, and services are critical under the Alma Ata right-to-health framework (25). Given the adequate health worker density and the systematic setup of training and refresher courses for the health workers in Karen State, task shifting has not demonstrated a detrimental effect on quality (26). Although this article did not seek to establish the direct effect of task shifting on population health status, it does show that a large number of people are receiving some level of vital health care in places where, otherwise, there would be none. This is coupled with the fact that EHOs and CBHOs generally administer clinical protocols that are recognized as effective at reducing morbidity and mortality in similar settings worldwide (27–31). For example, a relative comparative of proxy indicators from the household surveys conducted in 2008 and 2013 in the eastern Burma/Myanmar region showed indicative improvements in the population health status. For example, only 20% of children received vitamin A pills in 2008, but the percentage increased to 58.2% in 2013. Also, the prevalence of *Plasmodium falciparum* (PF) malaria was reduced from 6.9% in 2008 to 2.2% in 2013 (32, 33). This information clearly reflects the quality and importance of the health services provided by the existing health workforce. In the short and mid-run, task shifting increases the density of HRH reaching out to the underserved and hard-to-reach population.

### Task shifting and its relation to national HRH

Task shifting could be positioned as the entry point for policy engagement with the government. Task shifting to lower cadres of health workers among the EHOs and CBHOs only pertains to PHC provision. As Burma/Myanmar moves forward, PHC coverage will not be sufficient. Any arrangements to refer patients to either ethnic or national health systems will need to be tracked so that higher-level systems bottlenecks could also be addressed. If this issue of inadequate qualified health workers is not addressed, it could potentially reverse the success that task shifting has had to date.

In the long run, there is likely to be an increased demand for qualified health workers for the following reasons: 1) with a broader outreach to underserved and remote populations, health workers would connect those who are otherwise limited in access to qualified health workers; and 2) early detection of medical problems in underserved populations will thereafter increase demand on the formal health care system in the long run.

The EHOs and CBHOs are ready to discuss these topics with the Burmese government with the aim to improve national health system capacity, to promote investments across multiple disease areas and catalyze global improvements in health and survival, and to ensure that the ethnic populations that have not been served by the national health system will not be left behind.

### Recognition of parallel health systems

To support the health system strengthening of the Karen State, it is acknowledged that improved coordination among different EHOs and CBHOs is needed to reduce funding fragmentation. However, current challenges are the top-down and central-focus framework for implementing reform structures in the Burma/Myanmar health sector, with initiatives from the government to work with INGOs, EHOs, and CBHOs. By not acknowledging and building on the gains of the EHOs and CBHOs in health system strengthening, and by failing to acknowledge the meeting of health care needs through devolutive and distributive measures such as task shifting, top-down efforts risk duplicating services, engaging in inefficient funds distribution, and, most of all, alienating the local worker and ethnic communities.

This article broadly described the decentralized, community-based PHC system managed by EHOs and CBHOs with some secondary care offered by clinics. The referral mechanism is likely to be the weakest link between the parallel systems of the ethnic and government authorities (see Fig. 3). Pragmatic engagement between the two systems will extend the health care services closer to the communities where they are needed. In the long run, this engagement reduces costs on the national health system, and it ensures equity in access to health care with emphases on primary care and community reliance.

### Health systems reform in Burma/Myanmar and its relation to ASEAN integration

With the ongoing discussions on Association of Southeast Asian Nations (ASEAN) integration relating to migration and human mobility, it is crucial for the government, EHOs, and CBHOs to adapt their service delivery models to remain relevant. It is noteworthy to mention that for populations living in rural or remote areas with insufficient density of health workers, those who are closer to the borders will be likely to be accessing the providers across the Thailand border. Such diversion of care may increase inequities as those who lack resources often cannot afford travel costs.

A one-size-fits-all strategy and action plan will not work in the complex political and social contexts of the ASEAN region (34). In countries like the Philippines (35, 36) and Indonesia (37), whose governments have supported decentralization and the devolution of health services to rural and ethnic areas since 1991 and 2001, respectively, capacity and coordination challenges were reported with limited improved health outcomes. In Cambodia, however, the effectiveness of contracting non-state actors (NSAs) to deliver and manage services has been hampered by the widespread lack of transparency, the government’s failure to negotiate contracts openly, and the tendency of government officials to bypass laws and administrative processes in awarding contracts (38).

In the context of Burma/Myanmar, inequities in the coverage of health services are paralleled by similar disparities in the distribution of human and physical resources. Batley and Mcloughlin (39) mentioned that the most acute constraint on service provision specifically in conflict and postconflict situations is their dependence on not only the government’s capacity but also the capacity and willingness of NSAs, which will influence the potential for successful engagement.

As ASEAN integration gathers pace (40), whether the ethnic health systems will receive due recognition and practicing privileges is yet to be known. This article highlights the size, formality, and level of organization of the HRH in eastern Burma/Myanmar, and the EHOs’ and CBHOs’ willingness to engage with the government. This article attempts to serve as an important starting point for designing mutually beneficial forms of engagement in the area of HRH.

### Limitations and strengths of the study

We encountered a number of data limitations. First, although definitions and levels of training were similar across the organizations, how health workers operate in the field varies slightly. Second, organizations also adapted their service delivery models and medicine lists according to the funding requirements set by the donors. Finally, we had chosen to focus on analysis of data from HISWG and of data that are available based on the EHOs’ and CBHOs’ knowledge and experience of deploying health workers and providing services in conflict and postconflict periods.

This case study contributes to the limited literature on the role of task shifting in transitional and postconflict contexts like eastern Burma/Myanmar, where NSAs sought to be mainstream players in health system strengthening as well as service provision and management (39). This article does not seek to compare its findings with the health workforce of the government.

## Conclusion

Burma/Myanmar today is undergoing an unprecedented path of political and economic reforms. Improving access to and quality of care are actions of high priority among EHOs and CBHOs. Despite a landscape of chronic conflict until late 2010, this report provides evidence of the substantive system of health care provision and access over the years in eastern Burma/Myanmar. The constraints on funds flow and the level of engagement by external actors in this area during the last few years are imposing limitations on the delivery of basic services to the population in this region. This article underscores the climate of vulnerability of the EHOs and CBHOs due to a lack of regional and international understanding of this area’s political complexities as the media spotlight shines on the country’s economic and political reordering. Funding is highly fragmented with project-based services and programs, as challenges are typically addressed in a silo manner. The health workers are able to ensure basic health care to already vulnerable populations, but at the core of this has been a risk of loss of support in providing access to care.

## Footnotes

1 Burma/Myanmar is divided into zones, black zones, where free fire is allowed and the townships or villages are under ethnic administration; brown zones, where resistance groups are under control and the governance is of mix (ethnic and government) administration; and white zones where there is no fighting which means it is under government administration.

## Footnotes

2 The authors noted that WHO ratio of health care workers to population does not necessarily translate into “adequate” HRH. This paper does not set out to formally define the criteria that ensure ‘adequate’ services, e.g. is the mean a sufficient parameter to describe access to care?

## Footnotes

3 Other political organizations operating in the Karen/Kayin State could be the Democratic Karen Benevolent Army (DKBA) [formerly known as Democratic Karen Buddhist Army] and Border Guard Force (BGF). It should also be noted that both BMA and BPHWT operate beyond Karen/Kayin State, specifically BPHWT which also operates in Karenni, Mon States; Kayan, Shan Palaung, Pa-O and Lahu regions; as well as most recently Kachin and Arakan (Rakhine) states.

## Footnotes

4 The HISWG is a collaboration of seven (7) multi-ethnic EHOs and CBHOs as members [Back Pack Health Worker Team (BPHWT), Burma Medical Association (BMA), Karen Department of Health and welfare (KDHW), Karenni Mobile Health Committee (KnMHC), Mae Tao Clinic (MTC), Mon National Health Committee (MNHC) and Shan State Development Foundation (SSDF)], working together since 2002 to strengthen the health system for Eastern Burma/Myanmar, specifically through information/data sharing and management.

## Footnotes

5 It is noted that from the patient/villager perspectives, medical care might not be always available.

## References

[1] Assessing State Fragility (2012). A country indicators for foreign policy report. Relief Web.

[2] Ending the waiting game: strategies for responding to internally displaced people in Burma (2006). Refugees International.

[3] Chronic emergency: health and human rights in eastern Burma (2006). Back Pack Health Worker Team.

[4] Poverty, displacement and local governance in South East Burma/Myanmar (2006). The Border Consortium (TBC).

[5] Jolliffe K (2011). Dilemmas of Burma in transition. Armed non – state actors and displacement. Forced Migration Rev.

[6] Ministry of Health, Myanmar (2013). Health financing in Myanmar. Health in Myanmar.

[7] WHO (2009). Health expenditure.

[8] Ministry of Health, Myanmar (2013). Organization of health service delivery.

[9] Diagnosis: critical http://www.backpackteam.org/?p=730.

[10] Sipress A, Nakashima E In Burma, a Setback on AIDS.

[11] Stover E, Suwanvanichkij V, Moss A, Tuller D, Lee TJ, Whichard E The gathering storm: infectious diseases and human rights in Burma.

[12] WHO (2010). Aggregated data of health workforce density per 1000 by country.

[13] Kanchanachitra C, Lindelow M, Johnston T, Hanvoravongchai P, Lorenzo FM, Huong NL (2011). Human resources for health in southeast Asia: shortages, distributional challenges, and international trade in health services. Lancet.

[14] WHO (2008). Task shifting: rational redistribution of tasks among health workforce teams: global recommendations and guidelines.

[15] WHO Systems support for task – shifting to community health workers.

[16] Lwin MM, Sudhinaraset M, San AK, Aung T (2014). Improving malaria knowledge and practices in rural Myanmar through a village health worker intervention: a cross – sectional study. Malar J.

[17] Lim AG, Stock L, Shwe Oo EK, Jutte DP (2013). Trauma and mental health of medics in eastern Myanmar’s conflict zones: a cross – sectional and mixed methods investigation. Confl Health.

[18] Footer KHA, Meyer S, Sherman SG, Rubenstein L (2014). On the frontline of eastern Burma’s chronic conflict – listening to the voices of local health workers. Soc Sci Med.

[19] Teela KC, Mullany LC, Lee CI, Poh E, Paw P, Masenior N (2009). Community – based delivery of maternal care in conflict – affected areas of eastern Burma: perspectives from lay maternal health workers. Soc Sci Med.

[20] Isabelle Risso-Gill I, McKee M, Coker R, Piot P, Legido-Quigley H Health system strengthening in Myanmar during political reforms: perspectives from international agencies. Health Policy Plan.

[21] Mahn M, Maung C, Oo EKS, Smith L, Lee CI, Whichard E (2008). Multi – level partnerships to promote health services among internally displaced in eastern Burma. Glob Public Health.

[22] Mae Tao Clinic Mission statement.

[23] WHO World Health Statistics 2012.

[24] WHO Countdown to 2015 launches the 2012 report: fewer maternal and child deaths.

[25] UN Committee on Economic, Social and Cultural Rights (CESCR), General Comment No. 14: The Right to the Highest Attainable Standard of Health (Art. 12 of the Covenant), E/C.12/2000/4.

[26] WHO Systems support for task – shifting to community health workers.

[27] Heyman SN, Ginosar Y, Shapiro M, Kluger Y, Marx N, Maayan S (1997). Diarrheal epidemics among Rwandan refugees in 1994. Management and outcome in a field hospital. J Clin Gastroenterol.

[28] Caulfield LE, de Onis M, Blössner M, Black RE (2004). Undernutrition as an underlying cause of child deaths associated with diarrhea, pneumonia, malaria, and measles. Am J Clin Nutr.

[29] West KP (2004). Vitamin A deficiency as a preventable cause of maternal mortality in undernourished societies: plausibility and next steps. Int J Gynaecol Obstet.

[30] Hutagalung R, Paiphun L, Ashley EA, McGready R, Brockman A, Thwai KL (2005). A randomized trial of artemether – lumefantrine versus mefloquine – artesunate for the treatment of uncomplicated multi – drug resistant Plasmodium falciparum on the western border of Thailand. Mala J.

[31] WHO Guidelines for the treatment of malaria. Second edition.

[32] Parmar PK, Benjamin-Chung J, Smith LS, Htoo S, Laeng S, Lwin A (2014). Health and human rights in eastern Myanmar prior to political transition: a population-based assessment using multistaged household cluster sampling. BMC Int Health Hum Right.

[33] Parmar P, Barina C, Low S, Tun KT, Otterness C, Mhote PP Community “Health and human rights in eastern Myanmar after the political transition: a population – based assessment using multistaged household cluster sampling.”.

[34] Chongsuvivatwong V, Phua KH, Yap MT, Pocock NS, Hashim JH, Chhem R (2011). Health and health – care systems in southeast Asia: diversity and transitions. Lancet.

[35] Herrera MEB, Roman FL, Alarilla MCI Overview of health sector reform in the Philippines and possible opportunities for public – private partnerships. Asian Institute of Management (AIM). http://www.aim.edu/files/download/131.

[36] WPRO Philippines. http://www.wpro.who.int/asia_pacific_observatory/hits/series/phl/en/.

[37] Simatupang R (2009). Evaluation of decentralization outcomes in Indonesia: analysis of health and education sectors. Econ Diss.

[38] Rondinelli D Enhancing the public administration capacity of fragile states and postconflict societies: parallel and partnership approaches.

[39] Batley R, Mcloughlin C (2010). Engagement with non – state service providers in fragile states: reconciling state – building and service delivery. Dev Pol Rev.

[40] ASEAN Secretariat (2009). Initiative for ASEAN Integration (IAI) and Narrowing the Development Gap (NDG) (2009–2015). http://www.asean.org/communities/asean--economic--community/category/initiative--for--asean--integration--and--narrowing--the--development--gap.

# Sub article: Medical tourism in Malaysia: how can we better identify and manage its advantages and disadvantages?

**DOI:** 10.3402/gha.v7.25201

**Published:** 2014-09-10

**Authors:** Meghann Ormond, Wong Kee Mun, Chan Chee Khoon

**Affiliations:** 1 Cultural Geography Chair Group, Wageningen University, Wageningen, The Netherlands; 2 Faculty of Business and Accountancy, University of Malaya, Kuala Lumpur, Malaysia; 3 Centre for Population Health, Department of Social and Preventive Medicine, Faculty of Medicine, University of Malaya, Kuala Lumpur, Malaysia

**Keywords:** medical tourism industry, statistical data, destination countries, healthcare commodification, public–private health care investment, entrepreneurial state, Southeast Asia

## Abstract

Following the identification of medical tourism as a growth sector by the Malaysian government in 1998, significant government sector and private-sector investments have been channeled into its development over the past 15 years. This is unfolding within the broader context of social services being devolved to for-profit enterprises and ‘market-capable’ segments of society becoming sites of intensive entrepreneurial investment by both the private sector and the state. Yet, the opacity and paucity of available medical tourism statistics severely limits the extent to which medical tourism's impacts can be reliably assessed, forcing us to consider the real effects that the resulting speculation itself has produced and to reevaluate how the real and potential impacts of medical tourism are – and should be – conceptualized, calculated, distributed, and compensated for. Contemporary debate over the current and potential benefits and adverse effects of medical tourism for destination societies is hamstrung by the scant empirical data currently publicly available. Steps are proposed for overcoming these challenges in order to allow for improved identification, planning, and development of resources appropriate to the needs, demands, and interests of not only medical tourists and big business but also local populations.

Proponents generally envision how medical tourism – ‘all the activities related to travel and hosting a tourist who stays at least one night at the destination region, for the purpose of maintaining, improving or restoring health through medical intervention’ (1) – can be used by destinations to attract foreign exchange, mitigate health worker brain-drain, and improve health care and tourism infrastructure (2). Correspondingly, medical tourism has been actively embraced by governments and private-sector actors in a growing number of lower- and middle-income countries as a potentially powerful economic growth engine. Meanwhile, critics generally warn that medical tourism may harm destinations by stimulating private health care development unresponsive to locals’ needs and resources (3). Yet, although more scholars, governments, and medical bodies in source countries are calling attention to how medical tourism may adversely impact both source and destination societies (4), there has been relatively little outspoken resistance within destination countries to it.

Those engaging in contemporary debate over the current and potential benefits and adverse effects of medical tourism for destination societies generally turn to sorely inadequate government-reported medical tourism statistics – widely acknowledged to deploy opaque definitions and creative counting practices – to support their arguments (5). These statistics render medical tourism's actual volumes and contributions so difficult to gauge that the many estimates and claims made by those on either side of the debate should be treated with caution (6).

In Malaysia, one of Southeast Asia's most prominent medical tourism destinations, national medical tourism statistics derive from the reported numbers of all foreign patients treated by Malaysian Healthcare Travel Council–endorsed medical facilities and their associated revenue. These are published only at the national level, do not include all medical facilities, and do not recognize or measure medical tourism's diverse subnational direct and indirect economic and social impacts. Furthermore, available data indiscriminately encompass all registered patients with a foreign passport, which by default also encompass expatriates, migrants, business travelers, and holiday-makers for whom health care may not be the main motive for their stay (7–9). The opacity and paucity of available figures, therefore, severely limits the extent to which we can reliably quantify medical tourism's impacts in Malaysia. This quantitative void forces us both to consider the real effects that the speculation this void has itself produced and to reevaluate how the real and potential impacts of medical tourism are – and should be – conceptualized, calculated, distributed, and compensated for.

In this brief debate piece, we use the lenses of our diverse engagements with the Malaysian government, medical tourism industry, and health care providers and users to relate how medical tourism statistics have been used in Malaysia and reflect on the limitations of these framings. We then identify steps to more productively advance the discussion about the challenges and benefits of medical tourism.

## Proponents’ perspective

The Malaysian government identified medical tourism as a growth sector during the 1997–98 Asian financial crisis, when significant numbers of Indonesians began to turn to Malaysian private hospitals for affordable, quality health care. In the following decade, private hospitals – concentrated mainly in Penang, Melaka, Selangor, Sarawak, and Johor – worked alongside and through their respective state governments; private hospital associations; and the Malaysian Ministries of Health, Tourism, and Trade and Industry. They sought to attract not only neighboring Indonesians – characterized by high volumes yet low per patient expenditure – but also higher-spending medical tourists from further afield (e.g. Australia, Bangladesh, China, India, Japan, Nepal, the United Kingdom, the United States, and the Middle Eastern countries) (7, 10, 11). The Malaysian government's investment tax allowance further spurred private health care facilities promoting medical tourism to invest in internationally recognized accreditation schemes (e.g. Joint Commission International and Malaysian Society for Quality in Healthcare) and state-of-the-art medical equipment in order to develop technology-intensive private health care facilities and ensure ‘world-class’ care standards considered necessary to attract medical tourists (12, 1).

With the 2010 launch of the Economic Transformation Program (ETP), intended to transform Malaysia into an upper middle-income country with a knowledge-based economy, interest in harnessing medical tourism's economic potential grew. The ETP earmarked health care as one of the country's 12 National Key Economic Areas (NKEAs) deemed to have the potential to spur growth (13). Part of the health care NKEA, medical tourism is intended to generate MYR 9.6 billion^1^ in revenue and MYR 4.3 billion in gross national income and to require 5,300 more medical professionals by 2020 (14). For-profit hospitals are expected to invest MYR 335 million in hospital infrastructure in order to be prepared for 1.9 million foreign patients annually by 2020 (14). Despite the specificity of these targets, however, scant empirical data are publicly available to evaluate whether these targets are being met and, indeed, even the basis for such projections.

Medical tourism is believed to be contributing to the national economy. Government-reported revenue from medical tourism in Malaysia amounted to MYR 683 million – 9% above its 2013 target (15). Although this added only 0.1% to Malaysia's MYR 985 billion gross domestic product (GDP) in 2013 (16), medical tourism's year-on-year double-digit growth is being used to attract foreign investment and joint ventures in the Malaysian health care industry (e.g. the 2013 Ramsay Sime Darby Healthcare joint venture (17)). Large Malaysian health care conglomerates also plan to reap economic gains from medical tourism despite its current limited contribution. For example, although medical tourism contributed only 4% (MYR 67 million) to KPJ Healthcare Bhd's 2013 overall revenue, KPJ expects this to rise to 25% by 2020 by more intensively promoting its Malaysian facilities (15, 18).

Although growing numbers of Malaysian health care facilities are actively promoting medical tourism, some 95% of Malaysian private hospitals’ clientele is reported to be Malaysian (19). Private and corporatized hospitals’ medical tourism revenue, therefore, is viewed as helping to not only sustain but also upgrade these facilities to local private health care users’ benefit, providing Malaysians with alternatives to crowded public health care provision. Investment in medical tourism infrastructure is furthermore considered to generate demand for goods and services in allied sectors (e.g. clinical research and development, pharmaceuticals, and medical equipment) (14). Transport, retail, commercial care, and hospitality sectors can also benefit from spending by medical tourists and their companions, generating medical- and non-medical jobs and spurring the growth not just of large but also small and medium enterprises (1, 10). Local businesses in Kuching, Melaka, and Johor Bahru, for example, are seen to be flourishing with the influx of cross-border Indonesian and Singaporean medical tourists. However, to date, no empirical evidence is available on the multiplier effect of medical tourism on other sectors and local economies. Such data would help to elucidate the effectiveness of government investment in the growth of the medical tourism industry.

## Critics’ perspective

Malaysia's development as a medical tourism destination has unfolded within a context of health care corporatization and privatization that has profoundly transformed the country's health care landscape and horizons (20, 21). Critics see medical tourism as an expression of health care commodification, highlighting the Malaysian state's multiple roles as funder and provider of public-sector health care, regulator, and pre-eminent investor in commercial health care.

Medical tourism is embedded in a broader political economy in which social services have been devolved to for-profit enterprise and ‘market-capable’ segments of society have become sites of intensive entrepreneurial investment by both the private sector and the state (7). Critics note, for example, that, although private hospitals account for approximately 30% of all hospital admissions (22), government-linked companies at both federal and provincial levels currently control more than 40% of commercial hospital beds in Malaysia (23). Among the country's most prominent hospitals endorsed for medical tourism are for-profit hospitals belonging to the Johor State Government–owned KPJ chain and the IHH Healthcare Bhd–owned Pantai and Gleneagles chains. IHH, the world's second-largest listed health care operator based on market capitalization, is majority-owned by the Malaysian government's sovereign investment arm, Khazanah (24). Both KPJ and IHH command ever-larger slices of the Asian health care market both through their acquisition of regional hospitals (e.g. KPJ's acquisitions in Indonesia and Bangladesh) and their promotion of medical tourism in Malaysia.^2^

This novel situation is perceived to be rife with conflicts of interest and divergent priorities (7, 20, 21). Although health care is not inscribed in the Malaysian constitution as a right, Malaysian nationals have become accustomed to *de facto* entitlement to publicly provided and highly subsidized health care since decolonization in 1957. Citizens may or may not avail themselves of this universalist entitlement, yet even those who do not do so still benefit from its second-order effects. The availability of publicly provided health care (of a certain quality) acts as a restraining price bulwark that helps to keep private health care charges within a more affordable range. With the state's increasing stakes in commercial health care however, will there be a benign neglect of the public sector as the state encourages those who can afford it to migrate to the private sector for their healthcare needs? This could further entrench a two-tier health care system, with deluxe priority care for the better-off (including ‘medical tourists’) and a rump, underfunded public sector for the rest (25, 26).

Might Malaysians, however, benefit indirectly from profits accruing to the public purse from medical tourism and other for-profit healthcare investments? The Malaysian national oil company Petronas’ total equity is approximately ten times that of IHH. Comparing the MYR 87.8 million that IHH paid in corporate taxes to the Malaysian government in FY2011 with the MYR 66.3 billion in taxes and dividends generated by Petronas in FY2012 suggests modest returns on IHH's healthcare investments (27, 28). As for where revenue derived from medical tourism goes (e.g. special taxation regimes, economic leakages), little is known, and discussions about corporate accountability are absent.

## Conclusions

The debate over the gains and adverse effects of ‘medical tourism’ is far from Malaysia-specific. Rather, it is a concern shared by medical tourism destinations more generally (as in Israel (29) and Costa Rica (30)). However, given the great margin of uncertainty over basic data about medical tourism, conclusions on both sides are unavoidably speculative. Indeed, it is possible to assume – as we have shown here – diametrically opposed positions on the issue with little prospect of resolution.

There is much work to be done in order to better grasp medical tourism's actual impacts on destinations. This first requires us – as policymakers, industry actors, scholars, citizens, and consumers – to acknowledge not only medical tourism's imbrication in a broader range of transnational care pursuits and provision but also medical tourism's articulation in a broader (and increasingly global) political economy of health care. This would allow us to start asking more astute questions about the ways in which different stakeholders conceptualize ‘medical tourism’ and to begin to measure variables that enable analyses that transcend disembodied claims about growth (31).

This also requires us to acknowledge that medical facilities and the diverse communities in which they are inserted receive different volumes and types of foreign patients that may or may not be ‘medical tourists’, with their own unique needs, wants, socioeconomic and political statuses, and spending patterns. To better respond to this *de facto* diversity, the knowledge we produce about medical tourism must be useful to identify, plan for, and develop resources appropriate to the needs, demands, and interests of not only medical tourists and big business but also local populaces.

## Footnotes

1 The current exchange rate is USD 1=MYR 3.2.

## Footnotes

2 Currently, however, non-government-linked companies (e.g. Island Hospital and Penang Adventist Hospital) attract the lion's share of medical tourism to Malaysia.

## References

[1] Musa G, Doshi D, Wong KM, Thirumoorthi T (2012). How satisfied are inbound medical tourists in Malaysia? A study of private hospitals in Kuala Lumpur. J Trav Tour Mark.

[2] Bookman M, Bookman K (2007). Medical tourism in developing countries.

[3] Sengupta A (2011). Medical tourism: reverse subsidy for the elite. Signs.

[4] Snyder J, Dharamsi S, Crooks VA (2011). Fly-By medical care: conceptualizing the global and local social responsibilities of medical tourists and physician voluntourists. Global Health.

[5] Connell J (2011). Medical tourism.

[6] Wong KM, Musa G, Hall CM (2012). Medical tourism in Asia: Thailand, Singapore, Malaysia, and India. Medical tourism: the ethics, regulation, and marketing of health mobility.

[7] Ormond M (2013). Neoliberal governance and international medical travel in Malaysia.

[8] Malaysia Healthcare Travel Council (2013). Statistics. http://www.mhtc.org.my/en/statistics.aspx.

[9] Ministry of Health Malaysia (2014). Health facts 2014. http://www.moh.gov.my/images/gallery/publications/HEALTHFACTS2014.pdf.

[10] Ormond M, Sulianti D (2014). More than medical tourism: lessons from Indonesia and Malaysia on South-South intra-regional medical travel. Curr Issues Tour.

[11] Malaysia Healthcare Travel Council (2013). The first Medical Tourism Concierge and Lounge in Malaysia. http://www.mhtc.org.my/en/press-release.aspx.

[12] Johnston R, Crooks VA, Snyder J, Kingsbury P (2010). What is known about the effects of medical tourism in destination and departure countries? A scoping review. Int J Equity Health.

[13] Pemandu (2010). Economic transformation programme: a roadmap for Malaysia. http://etp.pemandu.gov.my/download_centre.aspx.

[14] Pemandu (2010). Creating wealth through excellence in healthcare. http://www.moh.gov.my/images/gallery/ETP/NKEAPenjagaanKesihatan.pdf.

[15] Pemandu (2013). ETP annual report, 2013. Healthcare. http://etp.pemandu.gov.my/annualreport2013/upload/ENG/14_NKEA12_ENG_HC.pdf.

[16] Bank Negara Malaysia (2014). National accounts (GDP) at current prices. http://www.bnm.gov.my/index.php?ch=statistic_nsdp.

[17] Lee L (2013). Ramsay JV aims to have at least 12 hospitals in three to five years. The Star.

[18] Teo R (2014). KPJ sees increasing contributions from Malaysian, medical tourism sectors. The Borneo Post.

[19] To Your Health [radio programme] (2014). The Breakfast Grille.

[20] Chan CK (2010). Re-inventing the welfarist state? The Malaysian health system in transition. J Contemp Asia.

[21] Chee HL (2010). Medical tourism and the state in Malaysia and Singapore. Glob Soc Policy.

[22] Ministry of Health Malaysia (2013). Health facts 2013. http://www.moh.gov.my/images/gallery/publications/HEALTHFACTS2013.pdf.

[23] Chan CK (2014). The Malaysian health system in transition: the ambiguity of public and private.

[24] IHH (2014). Overview. http://ihh-healthcare.com/hospital-overview.php.

[25] Chan CK, Ooi KB, Goh BL (2010). Re-thinking the state and healthcare in Penang. Pilot Studies for a New Penang.

[26] Chan CK (2006). What's new in the Arusha statement on *New Frontiers of Social Policy?*. Glob Soc Policy.

[27] IHH (2012). IHH undertakes first ever concurrent IPO in Malaysia and Singapore. http://ihh.anticsstudios.com/files/press-release/EnglishPressRelease_IHHIPOLaunch_3July.pdf.

[28] Petronas (2012). Petronas annual report 2012. http://www.petronas.com.my/investor-relations/Pages/annual-report.aspx.

[29] Zehavi A, Zer N (2013). A public road to globalization: public services and customer internationalization in Israel. Admin Soc.

[30] Lee CA (2012). Health care at a crossroads: medical tourism and the dismantling of Costa Rican exceptionalism.

[31] Pocock NS, Phua KH (2011). Medical tourism and policy implications for health systems: a conceptual framework from a comparative study of Thailand, Singapore and Malaysia. Global Health.

# Sub article: Disasters, resilience, and the ASEAN integration

**DOI:** 10.3402/gha.v7.25134

**Published:** 2014-09-10

**Authors:** Don Eliseo Lucero-Prisno

**Affiliations:** 1 Department of Public Health, Xi'an Jiaotong-Liverpool University, Suzhou, China; 2 Faculty of Management and Development Studies, University of the Philippines (Open University), Los Baños, Philippines

The recent havoc caused by typhoon Haiyan in central Philippines provides an example of the grim reality of the impact of disasters in the Association of South-East Asian Nations (ASEAN) region. This is personal to me as many of my relatives and friends died and many more had their properties all washed away. You see, I was born in Tacloban and grew up in the city. It pained me to see my 87-year-old grandmother, a veteran of many typhoons, struggling to survive amid the stench of death and destruction. No doubt, the President of the Philippines was prompted to echo the need to strengthen the framework of cooperation in managing disasters among the ASEAN countries, being one of the best approaches to address urgent issues and to build resilience.

Needless to say, many efforts have already been initiated and continued in reducing the vulnerability of the region to the risk of disasters in the context of sustainable development. This includes the establishment of the ASEAN Committee on Disaster Management (ACDM) in 2003, which the ASEAN body elevated to a full-fledged committee. The ASEAN also promulgated the Agreement on Disaster Management and Response (AADMER) which is a legal instrument that binds all member countries in promoting regional cooperation and collaboration so as to lessen disaster losses and having a joint emergency response to disasters. This document is a manifestation of the commitment of the ASEAN to the Hyogo Framework for Action 2005–2015 supported by 168 countries (1).

Many observers, including the Secretary-General of the ASEAN, believe that the tipping point in the vigorous supranational policy approach on disaster management was instigated by the Indian Ocean tsunami disaster in 2004. The scale of the devastation of the tsunami was so massive that people, not only from the region, but even those from beyond, realized that disasters are realities that could strike anytime, anywhere. ASEAN's rhetoric was hinged on six Rs – reduce disaster risks, rebound quickly, reinvigorate leadership, renovate the plan, respond better, and revive the ASEAN's sense of community. Many of these narratives have been echoed time and time again. This echo gets louder as disaster strikes. What is thought provoking in this rhetoric is the idea of constant reflection; thus, the key terms – ‘renovate’, ‘reinvigorate’, ‘better response’, and ‘revive’. If there is a constant need for changing interventions and approaches, does this mean that previous actions have remained insufficient?

This is probably the main impetus to push the discussions in the ASEAN and go beyond the level of consensus building and move vigorously away from rhetoric and pronouncements. As urgent actions are required in disasters, readily available resources and decisions should be at the disposal at the supranational level. This beckons for the need to have a strong coordinating body that can easily deploy immediate interventions at any geographical location. A substantial amount of relief fund should be readily available for immediate disposal and disbursement anywhere. This would wean away the region from too much reliance on donors that normally arrive after the critical period of search and rescue phase and comes in with their own philosophies and approaches. A cooperative framework would definitely benefit the countries that need most help, which apparently are the countries most affected by disasters. A good framework is also imperative for a regional relief fund to make it substantially sufficient to be significant in delivering impact.

A supranational framework and body would, however, face many challenges, as ASEAN countries are diverse in many different ways. The ASEAN, however, is cognizant of the disparities in economic and financial capabilities of the countries necessary to build and sustain the activities in building disaster management capabilities. To bring the lesser-equipped countries on par with the others, the organization included in their 2009–2015 strategic framework the assistance of countries such as Cambodia, Laos, Myanmar, and Vietnam in enhancing their capabilities in disaster responses, and search and rescue by organizing training courses and workshop; provision of support through equipment and infrastructure for search and rescue and disaster responses; and providing more capacity building in disaster management and emergency response.

History has shown that time and time again, the ASEAN region will see more disasters. The region is prone to numerous hazards, big and small, that result in the accumulation of many losses of lives and properties. Its geographical location makes it vulnerable being placed in between two big oceans – the Pacific and Indian oceans – resulting in many typhoons, floods, landslides, and storm surges. Earthquakes, tsunamis, and volcanic eruptions are common occurrences as the region lies in between a number of tectonic plates. There are also forest fires and a number of much-publicized epidemics such as SARS, H1N1, and H9N7 that have caused havoc and hardships among the populations affected (2). MERS and the Ebola virus are a major scare as they pose threats to the region given the number of ASEAN migrant workers in the affected parts of the world.

According to the report of the ASEAN Disaster Risk Management Initiative (2), from 1970 to 2009 there were 1,211 reported disasters that comprised floods (36%), cyclonic storms (32%), earthquakes and tsunami (9%), epidemics (8%), landslides (7%), volcanic eruptions (4%), droughts (3%), and forest fires (1%). There were 414,927 deaths out of these reported disasters. The economic impact of all these disasters is so immense affecting the personal level to the regional level. There is also erosion of the health system, which renders governments unable to cope with the massive and quick rise of health needs of the population.

For example, the recent Haiyan disaster in the Philippines on 8 November 2013 resulted in more than 6,000 deaths, with 1,700 still missing, and 27,000 injured. The devastation has affected 14 million people, including some 5 million children. A total of 3.9 million people were forced from their homes. The United Nations and aid groups called to raise US$791 million to assist those affected (3). Reports show that the impact of the typhoon would have been minimized had there been better management of the risks prior, during and after the typhoon. Deaths were mostly due to the surge of water as people were caught unaware (4).

The ASEAN region also faces the challenges of ‘emerging’ disasters. These new ‘forms’ undoubtedly beg for a cooperative approach. For example, Malaysia Airlines Flight 370 may have directly affected few lives; however, its psychological impact on tourists and travelers was quite significant. The search for the plane and the people on board was an example why a multi-country effort was essential. Then Malaysia Airlines Flight 17 was another ‘global disaster’ that provided an impetus for a strong ASEAN stand. Albeit political, a unified voice of 10 countries with its innocent citizens killed can push swift actions at the international level. The same is true with political conflicts that resemble disaster scenarios such as the riots in Vietnam against Chinese nationals, the civil strife in Thailand, insurgency in southern Philippines, and the minority issues in Myanmar where political solutions become imperative.

Nuclear disasters such as Chernobyl and Fukushima may become a major issue in the future as countries such as Vietnam are planning four power reactors with the Philippines, Indonesia, Malaysia, Vietnam, and Thailand having research reactors (5). Obviously, a meltdown in China would directly affect the ASEAN. Oils spills, pollution, and poisoning of water systems from factories and mines, maritime piracy, and drug abuse should not be discounted.

Most of all, the impact of climate change should be seriously factored in as a transnational issue. As temperature increases sea level rises and drastic weather becomes more frequent resulting in more calamities (2). This trend is being observed in the region, which already showed massive effects on coastal populations, densely populated places, and the agricultural economy that populations are dependent on.

Yet, time and time again, we always see hope amid all this misery. Economies rebound and the populations recover despite the upheaval brought about by the havoc of calamities. Resilience is always a good story and is shown to be an enduring phenomenon. No matter how long the impact affects the populace, they become survivors and not victims; they pick up the pieces and move on with their lives. Yes, resilience is an area that is never focused on. Not even in the field of disaster research. This is why research becomes imperative and should be a major component of the approach in a unified disaster management framework. Documentation and research should be central in a supranational effort, as there is much learning that is yet to be discovered.

There are no arguments that counter the need for a strong and firm cooperative ASEAN effort in developing resilience against disasters – vulnerability is high, disasters are getting stronger and more frequent, and uniting small countries is prudent in pooling resources. An ASEAN disaster body with a strong mandate from the member governments in the context of an integrated ASEAN may be the impetus toward innovative and novel approaches in disaster preparedness and prevention and in cooperating to protect civilians. This might be the answer to the chaos that transpired during the Haiyan disaster in the Philippines. Hopefully, my grandmother would live to see the fruition of immediate and rapid responses during calamities. She already had enough with 87 years of disasters. And time is of the essence.

## References

[1] United Nations Office for Disaster Risk Reduction (2012). Synthesis report consultations on a post-2015 framework on disaster risk reduction (HFA2).

[2] United Nations Office for Disaster Risk Reduction (2010). Synthesis report on ten ASEAN countries disaster risk assessment.

[3] USAID (2013). Philippines – Typhoon Yolanda/Haiyan Fact Sheet No. 14.

[4] WHO (2013). Health response – Typhoon Haiyan (Yolanda).

[5] World Nuclear Association (2014). Asia's Nuclear Energy Growth. http://www.world-nuclear.org/info/country-profiles/others/asia-s-nuclear-energy-growth/.

# Sub article: Role of occupational health in managing non-communicable diseases in Brunei Darussalam

**DOI:** 10.3402/gha.v7.25594

**Published:** 2014-11-06

**Authors:** Pg Khalifah Pg Ismail, David Koh

**Affiliations:** 1 Department of Health Services, Ministry of Health, Bandar Seri Begawan, Brunei Darussalam; 2 Occupational Health and Medicine, Universiti Brunei Darussalam, Bandar Seri Begawan, Brunei Darussalam; 3 SSH School of Public Health, National University of Singapore, Singapore

**Keywords:** non-communicable diseases, occupational health

## Abstract

Like most ASEAN countries, Brunei faces an epidemic of non-communicable diseases. To deal with the complexity of NCDs prevention, all perspectives - be it social, familial or occupational – need to be considered. In Brunei Darussalam, occupational health services (OHS) offered by its Ministry of Health, among others, provide screening and management of NCDs at various points of service. The OHS does not only issue fitness to work certificates, but is a significant partner in co-managing patients’ health conditions, with the advantage of further management at the workplace. Holistic approach of NCD management in the occupational setting is strengthened with both employer and employee education and participation, targeting several approaches including risk management and advocating healthy lifestyles as part of a healthy workplace programme.

Brunei Darussalam, with a population of over 400,000, currently faces an increasing burden of non-communicable diseases (NCDs) such as diabetes, heart disease, and cancer. NCDs account for over half of all deaths locally (1), with the four leading causes being cancer, heart diseases, diabetes mellitus, and cerebrovascular diseases. This is similar to other Southeast Asian countries (2), where chronic NCDs account for 60% of deaths. The underlying causes for this include smoking, unhealthy diets, and inadequate physical activity. Preliminary results of a recent national survey on health and nutrition (3) showed the number of obese adults almost doubling since the last study in 1998.

As a Member Country of the Association of South East Asian Nations (ASEAN) and World Health Organization (WHO), Brunei Darussalam actively participates and contributes in WHO and ASEAN health-related meetings. The country's commitment to tackle NCDs was reflected when the Bandar Seri Begawan Declaration on NCDs in ASEAN was successfully adopted during the 23rd ASEAN Summit in 2013 (4). His Majesty the Sultan of Brunei Darussalam has repeatedly stressed the importance of the nation's health, including the impact of NCDs and called for sustainable actions in adhering to healthier lifestyles in tackling NCDs^1^

To deal with the complexity of NCDs, all perspectives – be it social, familial, or occupational – need to be considered. NCD prevention and control need a holistic, integrated, and multilevel approach requiring strong and sustained commitment and actions from all sectors to address NCDs. Within the working population, work and workplace risk factors can cause or exacerbate NCDs.

With this in mind, occupational health services (OHS) offered by the Brunei Darussalam's Ministry of Health, among others, provide screening and management of NCDs at various points of service – particularly at entry and at periodic intervals for specific jobs as well as walk-in clinics and referrals from other specialties. These clinical activities complement other essential OHS activities such as workplace surveillance, workplace health advice and promotion, as well as workmen's compensation issues. Thus, a holistic approach to management of workers’ health status, workplace environment as well as policy change can be implemented.

This approach is in line with WHO recommendations of workplaces as healthy settings to promote health, including interventions for NCDs. It is also in accord with the WHO Global Plan of Action on Workers Health 2008–2017, which was adopted at the 60th World Health Assembly (5). Risk factors and hazards at the workplace – for example, sedentary work, work stress, exposure to carcinogens, shift work, unsupportive environments, such as provision of unhealthy food at workplaces – which can contribute to NCDs can be effectively addressed through these initiatives.

OHS in Brunei Darussalam is still relatively new as compared to other well-established health services such as community health. Workers in Brunei Darussalam are exposed to a plethora of risks and hazards at the workplace. This is no different from other workers in the same job description in other parts of the world; but to varying degrees. This could result in a multitude of health outcomes, including NCDs. As an example, sedentary work, coupled with physical inactivity and unhealthy diets predispose to overweight and obesity.

The Integrated Health Screening and Health Promotion Programme among Civil Servants (IHSHP) 2007–2011, which was spearheaded by the Health Promotion Centre in Brunei, screened 21,437 civil servants from 12 government ministries. The population screened were females (55%), with varying age groups – 20–29 (15%), 30–39 (29%), 40–49 (35%), 50–59 (20%), and above 60 (1%). Results showed that 38% of civil servants were overweight and another 28% obese. Thirty-eight per cent of those screened had high blood pressure, 11% had high fasting blood glucose, and 25% high fasting blood cholesterol levels (6). The prevalence of self-reported stress was 49.5%, with females significantly more stressed (54.1%) compared to males (44.7%). Also, 23% of those screened reported not doing any exercise at all.

Data on specific parameters obtained from government OHS clinic activities also give cause for concern. In these OHS clinics, clients were exclusively from various government sectors which mandate a pre-employment medical fitness examination, as well as from private sectors requiring or requesting such assessment. These occupations include commercial airline staff, seafarers, construction, and mining workers. For certain jobs such as physicians and nurses in healthcare, police, fire and rescue, and aviation personnel, coverage for such examinations is 100%. For other sectors, such as manufacturing, periodic medical fitness to work examinations may be mandatory for high-risk jobs, but not required for low-risk jobs.

In 2012 (7), among approximately 3,500 clients assessed, 75% (compared to 68% in 2011) had undesirable BMI, 42% (compared to 39% in 2011) had elevated blood cholesterol levels, and 13% had raised blood pressure. Clients were referred to relevant specialties if further medical management was indicated. Workplace assessments were conducted when required, for example, workplace assessment of stress coupled with peer counselling and psychiatrist referral for a client with poor superior – employee relationship.

With efficient co-management of NCDs with primary healthcare doctors and other specialists, the OHS can serve as a gatekeeper for NCD screening at an earlier stage. This is not just for younger persons, for example, at the pre-employment stage, but also for older workers who are not concerned about their health status unless warranted by their employers through mandatory health screenings.

It is important that these screening programmes are not meant to be discriminatory against the employee. This applies equally to a prospective employee for certification of fitness for employment, or to someone in an existing job for termination or medical downgrading. Rather, the screening is meant for secondary prevention and also for the purpose of educating the employee and employer on the importance of health in the workplace. It also provides means and ways for an amicable win–win solution to be achieved should any adverse situation arise.

Thus, the OHS in Brunei Darussalam does not only issue fitness to work certificates, but is a significant partner in co-managing patients’ health conditions, with the advantage of further management at the workplace. The holistic approach of NCD management in the occupational setting is strengthened by employer and employee education and participation, targeting several approaches including risk management and advocating healthy lifestyles as part of a healthy workplace programme.

Several health guidelines and policies in Brunei Darussalam have been prepared with the involvement of the OHS (8). The OHS plays a pivotal role and contributes to the Ministry's and national vision and goal of addressing NCDs in alignment with the objectives of the Brunei National Multisectoral Action Plan for Prevention and Control of Non-Communicable Diseases (BruMAP-NCD) (8).

Nevertheless, challenges remain in providing a comprehensive OHS service to the working population. While Brunei Darussalam has a doctor and nurse density per 1,000 population of 1.1 and 6.1, respectively, which is among the highest in ASEAN (just behind Singapore and the Philippines) (9), occupational health is still considered a ‘unique specialty’ with less than 10 trained occupational health physicians in the government service at any one time. Coverage by the government service is estimated to be less than 15% of the entire working population. Furthermore, comprehensive data from OHS specifically pertaining to NCDs is very basic, with only available retrievable data on absolute numbers and percentage of clients within the job category assessed with specific NCDs.

The lack of availability of trained personnel and low coverage provided by the government sector is however partially offset by the presence of occupational health personnel and OHS in the private sector, for example, in the oil and gas industry, and appointed occupational health physicians in the private sector. Advocacy, increased awareness among all stakeholders, improved data collection, and management with opportunities for research as well as collaboration with relevant sectors will contribute to a more integrated approach in the efforts by OHS to address NCDs within the working population.

Despite these challenges, the government is committed in striving for better and ultimately optimal health for the Bruneian population, through various initiatives and policy directions such as the adoption of Health in All Policies approach. There is increased awareness from various sectors reflected by the rising demand for healthy workplace initiatives and programmes, following efforts in dissemination of statistics and dangers of NCDs and results of the IHSHP. Additionally, the OHS will continue to contribute to the ASEAN cause, such as through activities coordinated by Brunei Darussalam within the ASEAN Task Force on NCDs in the development of key indicators for healthy lifestyles and the monitoring and evaluation processes.

## Footnotes

1 Titah of His Majesty Sultan Haji Hassanal Bolkiah Mu'izzaddin Waddaulah ibni Al-Marhum Sultan Haji Omar ‘Ali Saifuddien Sa'adul Khairi Waddien, Sultan and Yang Di-Pertuan of Brunei Darussalam, on the occasion of the New Year's Day 2012, on the occasion of His Majesty's 66th Birthday 2012 and on the occasion of the Knowledge Convention (Majlis Ilmu) 2012.

## References

[1] Health Information Booklet (2012). Ministry of Health, Brunei Darussalam. http://ghdx.healthdata.org/record/brunei-darussalam-health-information-booklet-2012.

[2] Dans A, Ng N, Varghese C, Tai ES, Firestone R, Bonita R (2011). The rise of chronic non-communicable diseases in Southeast Asia: time for action. Lancet.

[3] Ministry of Health, Brunei Darussalam (2012). 2nd National Health and Nutritional Status Survey 2009–2011.

[4] Bandar Seri Begawan Declaration on Non-Communicable Diseases in ASEAN (2013). http://www.asean.org/asean/asean-structure/item/twentythird-aseansummit-bandar-seri-begawan-brunei-darussalam-9-10-october-2013.

[5] (2013). WHO Global Plan of Action on Workers Health 2008–2017. http://www.who.int/occupational_health/who_workers_health_web.pdf.

[6] Ministry of Health (2012). Annual Report, Occupational Health Division, Department of Health Services.

[7] Ministry of Health (2012). Integrated Health Screening & Health Promotion Programme among Civil Servants (IHSHP) 2007–2011.

[8] Health Promotion Blueprint 2011–2015 Ministry of Health, National Physical Activity Guidelines for Brunei Darussalam, Ministry of Health and the Brunei National Multisectoral Action Plan for Prevention and Control of Non Communicable Diseases (BruMAP-NCD) 2013–2018, Ministry of Health. http://ftp://ftp.wpro.who.int/scratch/NHP/NCD/NCD-policiesWPR/BRN/FINALBRUMAPBOOK.pdf.

[9] Kanchanachitra C, Lindelow M, Johnston T, Hanvoravongchai P, Lorenzo FM, Huong NL (2011). Human resources for health in Southeast Asia: shortages, distributional challenges, and international trade in health services. Lancet.
